# Supplementary material for: Genome-Wide and Comprehensive Analysis of the Multiple Stress-Related CAF1 (CCR4-Associated Factor 1) Family and Its Expression in Poplar
Source: Plants (Basel). 2021 May 14;10(5):981. doi: 10.3390/plants10050981 (PMC8155972; doi:10.3390/plants10050981)
Supplement: Supplementary file 1 [file plants-10-00981-s001.zip › Supplementary figure.pdf]

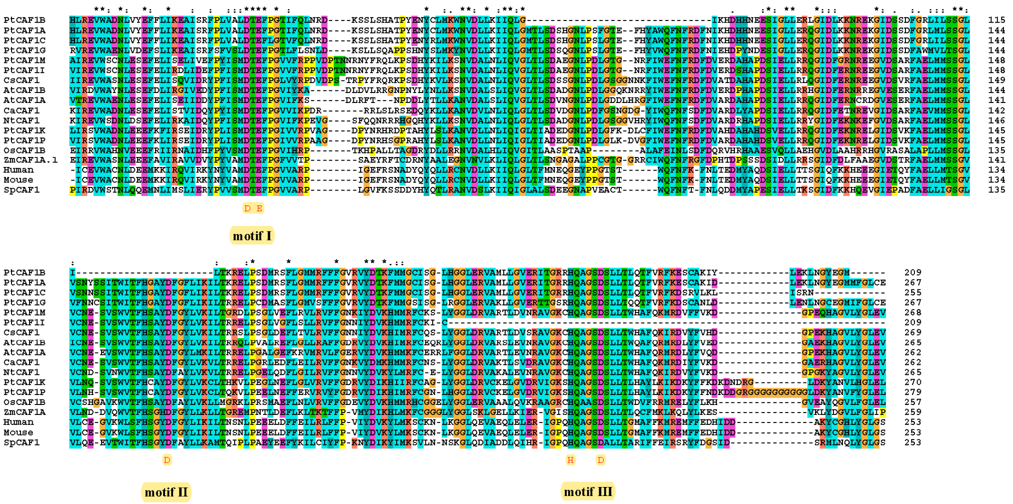

Supplementary figure 1. ClustalX2 generated alignment of CAF1 protein sequences from A. thaliana, O.sativa, Capsicum annuum, Citrus sinensis, Nicotiana tabacum, yeast (SpCAF1), human (HsCNOT7), mouse, and Zea mays (ZmCAF1). Conserved RNase D-domain residues are mark below the sequence.

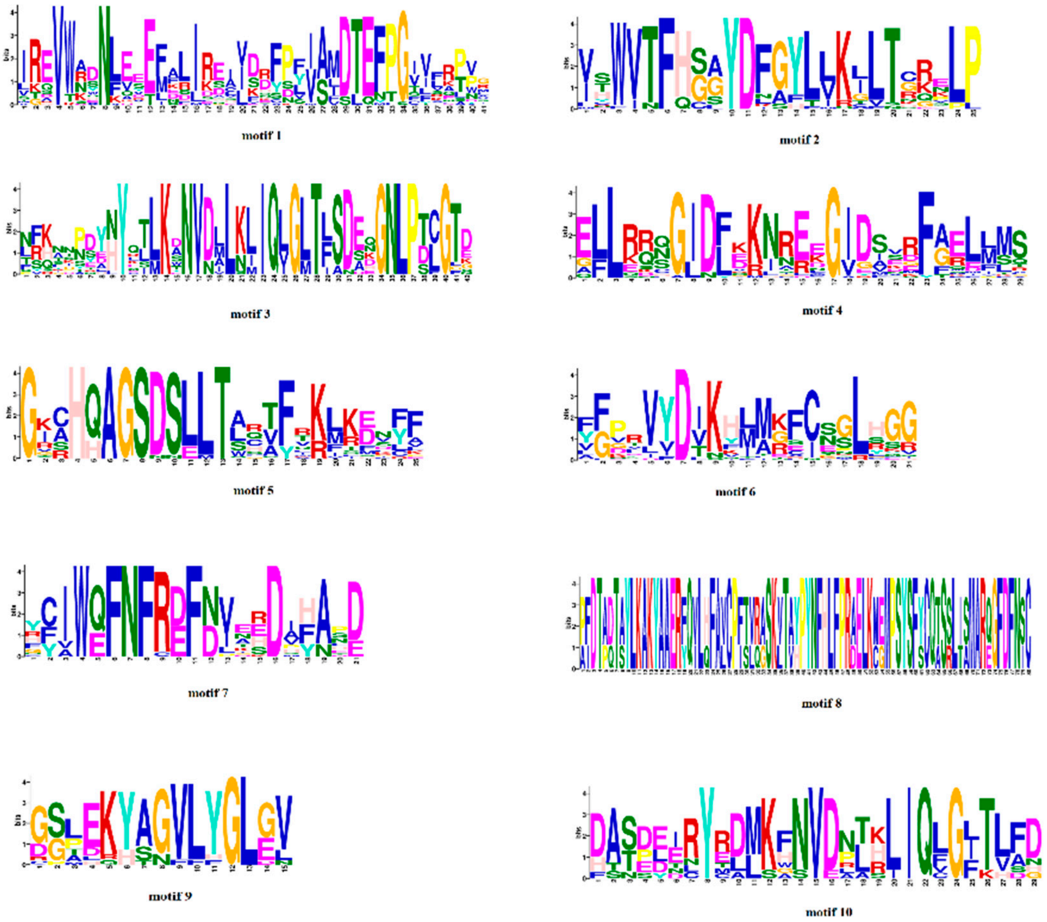

Supplementary figure 2. A list of the motifs from motif1 to motif10 in CAF1 gene family by MEME analysis. The alphabet stands for amino acid. The size of alphabet indicates conservatism. The larger the alphabet, the more conservative the amino acid.

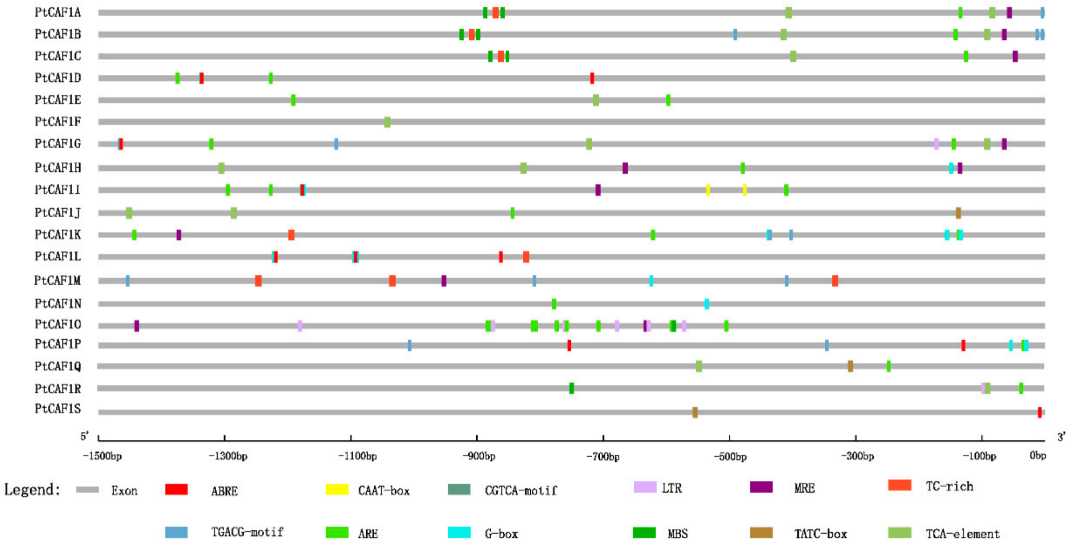

Supplementary figure 3. Prediction of the cis-regulatory elements in the promoters. Upstream 1500 bp sequences of each gene promoter were analyzed in the PlantCARE server. Stress-related cis-regulatory elements are spotted in different colors.
