# Supplementary material for: Genome-Wide and Comprehensive Analysis of the Multiple Stress-Related CAF1 (CCR4-Associated Factor 1) Family and Its Expression in Poplar
Source: Plants (Basel). 2021 May 14;10(5):981. doi: 10.3390/plants10050981 (PMC8155972; doi:10.3390/plants10050981)
Supplement: Supplementary file 1 [file plants-10-00981-s001.zip › Supplementary table.pdf]

Table S1: Cis-acting elements of *PtCAF1* in promoter regions.

gene ID	Cis-acting elements	sequence	species	Specific information
PNS9 2197	TATA-box	TATA	Arabidopsis thaliana	core promoter element around -30 of transcription start
PNS9 2197	TATA-box	ATATAT	Brassica napus	core promoter element around -30 of transcription start
PNS9 2197	TATA-box	TATA	Arabidopsis thaliana	core promoter element around -30 of transcription start
PNS9 2197	TATA-box	TATTTAAA	Arabidopsis thaliana	core promoter element around -30 of transcription start
PNS9 2197	TATA-box	ATATAT	Brassica napus	core promoter element around -30 of transcription start
PNS9 2197	TATA-box	TATA	Arabidopsis thaliana	core promoter element around -30 of transcription start
PNS9 2197	TATA-box	TATAA	Arabidopsis thaliana	core promoter element around -30 of transcription start
PNS9 2197	TATA-box	TATA	Arabidopsis thaliana	core promoter element around -30 of transcription start
PNS9 2197	TATA-box	ATTATA	Brassica napus	core promoter element around -30 of transcription start
PNS9 2197	TATA-box	TATAA	Arabidopsis thaliana	core promoter element around -30 of transcription start
PNS9 2197	TATA-box	TATA	Arabidopsis thaliana	core promoter element around -30 of transcription start
PNS9 2197	TATA-box	TATAAAA	Pisum sativum	core promoter element around -30 of transcription start
PNS9 2197	TATA-box	TATAAA	Helianthus annuus	core promoter element around -30 of transcription start
PNS9 2197	TATA-box	TATAA	Arabidopsis thaliana	core promoter element around -30 of transcription start
PNS9 2197	TATA-box	TATA	Arabidopsis thaliana	core promoter element around -30 of transcription start
PNS9 2197	TATA-box	TATTTAAA	Arabidopsis thaliana	core promoter element around -30 of transcription start
PNS9 2197	TATA-box	ATTATA	Brassica napus	core promoter element around -30 of transcription start
PNS9 2197	TATA-box	TATAA	Arabidopsis thaliana	core promoter element around -30 of transcription start
PNS9 2197	TATA-box	TATA	Arabidopsis thaliana	core promoter element around -30 of transcription start
PNS9 2197	TATA-box	TATA	Arabidopsis thaliana	core promoter element around -30 of transcription start

PNS9 2197	TATA-box	TATATTTATATT T	Avena sativa	core promoter element around -30 of transcription start
PNS9 2197	TATA-box	ATATAT	Brassica napus	core promoter element around -30 of transcription start
PNS9 2197	TATA-box	TATATA	Arabidopsis thaliana	core promoter element around -30 of transcription start
PNS9 2197	TATA-box	ATATAT	Brassica napus	core promoter element around -30 of transcription start
PNS9 2197	TATA-box	TATA	Arabidopsis thaliana	core promoter element around -30 of transcription start
PNS9 2197	TATA-box	ATATAT	Brassica napus	core promoter element around -30 of transcription start
PNS9 2197	TATA-box	TATA	Arabidopsis thaliana	core promoter element around -30 of transcription start
PNS9 2197	TATA-box	TATAAATA	Daucus carota	core promoter element around -30 of transcription start
PNS9 2197	TATA-box	TATAAAT	Brassica juncea	core promoter element around -30 of transcription start
PNS9 2197	TATA-box	TATAAA	Helianthus annuus	core promoter element around -30 of transcription start
PNS9 2197	TATA-box	TATAA	Arabidopsis thaliana	core promoter element around -30 of transcription start
PNS9 2197	TATA-box	TATA	Arabidopsis thaliana	core promoter element around -30 of transcription start
PNS9 2197	TATA-box	TATA	Arabidopsis thaliana	core promoter element around -30 of transcription start
PNS9 2197	TATA-box	ccTATAAAaa	Arabidopsis thaliana	core promoter element around -30 of transcription start
PNS9 2197	TATA-box	TATATA	Arabidopsis thaliana	core promoter element around -30 of transcription start
PNS9 2197	TATA-box	TATA	Arabidopsis thaliana	core promoter element around -30 of transcription start
PNS9 2197	TCA-element	CCATCTTTTT	Nicotiana tabacum	cis-acting element involved in salicylic acid responsiveness
PNS9 2197	STRE	AGGGG	Arabidopsis thaliana	
PNS9 2197	STRE	AGGGG	Arabidopsis thaliana	
PNS9 2197	AT-rich element	ATAGAAATCAA	Glycine max	binding site of AT-rich DNA binding protein (ATBP-1)
PNS9 2197	O2-site	GATGATGTGG	Zea mays	cis-acting regulatory element involved in zein metabolism regulation
PNS9 2197	Myc	TCTCTTA	Arabidopsis thaliana	

PNS9 2197	TATC-box	TATCCCA	Oryza sativa	cis-acting element involved in gibberellin-responsiveness
PNS9 2197	W box	TTGACC	Arabidopsis thaliana	
PNS9 2197	W box	TTGACC	Arabidopsis thaliana	
PNS9 2197	W box	TTGACC	Arabidopsis thaliana	
PNS9 2197	ARE	AAACCA	Zea mays	cis-acting regulatory element essential for the anaerobic induction
PNS9 2197	CAAT-box	CAAAT	Pisum sativum	common cis-acting element in promoter and enhancer regions
PNS9 2197	CAAT-box	CAAT	Nicotiana glutinosa	
PNS9 2197	CAAT-box	CAAT	Nicotiana glutinosa	
PNS9 2197	CAAT-box	CAAT	Nicotiana glutinosa	
PNS9 2197	CAAT-box	CCAAT	Arabidopsis thaliana	common cis-acting element in promoter and enhancer regions
PNS9 2197	CAAT-box	CAAT	Nicotiana glutinosa	
PNS9 2197	CAAT-box	CCAAT	Arabidopsis thaliana	common cis-acting element in promoter and enhancer regions
PNS9 2197	CAAT-box	CAAT	Nicotiana glutinosa	
PNS9 2197	CAAT-box	CAAT	Nicotiana glutinosa	
PNS9 2197	CAAT-box	CAAT	Nicotiana glutinosa	
PNS9 2197	CAAT-box	CAAAT	Pisum sativum	common cis-acting element in promoter and enhancer regions
PNS9 2197	CAAT-box	CCAAT	Arabidopsis thaliana	common cis-acting element in promoter and enhancer regions
PNS9 2197	CAAT-box	CAAT	Nicotiana glutinosa	
PNS9 2197	CAAT-box	CAAT	Nicotiana glutinosa	
PNS9 2197	CAAT-box	CAAT	Nicotiana glutinosa	
PNS9 2197	CAAT-box	CAAAT	Pisum sativum	common cis-acting element in promoter and enhancer regions
PNS9 2197	CAAT-box	CAAT	Nicotiana glutinosa	

PNS9 2197	CAAT-box	CAAT	Nicotiana glutinosa	
PNS9 2197	CAAT-box	CAAAT	Pisum sativum	common cis-acting element in promoter and enhancer regions
PNS9 2197	CAAT-box	CAAT	Nicotiana glutinosa	
PNS9 2197	CAAT-box	CAAAT	Pisum sativum	common cis-acting element in promoter and enhancer regions
PNS9 2197	CAAT-box	CAAAT	Pisum sativum	common cis-acting element in promoter and enhancer regions
PNS9 2197	CAAT-box	CAAT	Nicotiana glutinosa	
PNS9 2197	CAAT-box	CCAAT	Arabidopsis thaliana	common cis-acting element in promoter and enhancer regions
PNS9 2197	CAAT-box	CAAAT	Pisum sativum	common cis-acting element in promoter and enhancer regions
PNS9 2197	CAAT-box	CAAT	Nicotiana glutinosa	
PNS9 2197	WUN-motif	AAATTACTA	Nicotiana glutinosa	
PNS9 2197	AAGAA-motif	GAAAGAA	Avena sativa	
PNS9 2197	AAGAA-motif	GAAAGAA	Avena sativa	
PNS9 2197	AAGAA-motif	gGTAAAGAAA	Avena sativa	
PNS9 2197	chs-CMA1a	TTACTTAA	Daucus carota	part of a light responsive element
PNS9 2197	AT1-motif	AATTATTTTTTA TT	Solanum tuberosum	part of a light responsive module
PNS9 2197	MYC	CAATTG	Arabidopsis thaliana	
PNS9 2197	MYC	CATGTG	Arabidopsis thaliana	
PNS9 2197	Unnamed__4	CTCC	Petroselinum hortense	
PNS9 2197	Unnamed__4	CTCC	Petroselinum hortense	
PNS9 2197	Unnamed__4	CTCC	Petroselinum hortense	
PNS9 2197	chs-CMA2a	TCACTTGA	Petroselinum crispum	part of a light responsive element
PNS9 2197	AT~TATA-box	TATATA	Arabidopsis thaliana	

PNS9			Arabidopsis	
2197	AT~TATA-box	TATATA	thaliana	
PNS9			Arabidopsis	
2197	MYB	CAACCA	thaliana	
PNS9			Arabidopsis	
2197	MYB	CAACCA	thaliana	
PNS9			Arabidopsis	
2197	MYB	CAACCA	thaliana	
PNS9			Nicotiana	
2197	ERE	ATTTCATA	glutinos	
PNS9		motif_sequen		
2197		ce	organism	short_function
PNS9		motif_sequen		
2197		ce	organism	short_function
PNS9		motif_sequen		
2197		ce	organism	short_function
PNS9		motif_sequen		
2197		ce	organism	short_function
PNS9		motif_sequen		
2197		ce	organism	short_function
PNS9		motif_sequen		
2197		ce	organism	short_function
PNS9			Petroselinum	part of a conserved DNA module involved
2197	Box 4	ATTAAT	crispum	in light responsiveness
PNS9			Petroselinum	part of a conserved DNA module involved
2197	Box 4	ATTAAT	crispum	in light responsiveness
PNS9				cis-regulatory element involved in
2197	GCN4_motif	TGAGTCA	Oryza sativa	endosperm expression
PNS9			Arabidopsis	
2197	TATA	TATAAAAT	thaliana	
PNS9			Arabidopsis	
2197	TATA	TATAAAAT	thaliana	
PNS9				
2197	WRE3	CCACCT	Pisum sativum	
PNS9				
2197	WRE3	CCACCT	Pisum sativum	
PNS9				
2197	Unnamed__6	taTAAATATct	Zea mays	
PNS9				core promoter element around -30 of
2495	TATA-box	TATAAAA	Pisum sativum	transcription start
PNS9			Helianthus	core promoter element around -30 of
2495	TATA-box	TATAAA	annuus	transcription start
PNS9			Arabidopsis	core promoter element around -30 of
2495	TATA-box	TATAA	thaliana	transcription start

PNS9 2495	TATA-box	TATA	Arabidopsis thaliana	core promoter element around -30 of transcription start
PNS9 2495	TATA-box	TATAAAA	Pisum sativum	core promoter element around -30 of transcription start
PNS9 2495	TATA-box	TATAAA	Helianthus annuus	core promoter element around -30 of transcription start
PNS9 2495	TATA-box	TATAA	Arabidopsis thaliana	core promoter element around -30 of transcription start
PNS9 2495	TATA-box	TATA	Arabidopsis thaliana	core promoter element around -30 of transcription start
PNS9 2495	TATA-box	TATAAAT	Brassica juncea	core promoter element around -30 of transcription start
PNS9 2495	TATA-box	TATAAA	Helianthus annuus	core promoter element around -30 of transcription start
PNS9 2495	TATA-box	TATAA	Arabidopsis thaliana	core promoter element around -30 of transcription start
PNS9 2495	TATA-box	TATA	Arabidopsis thaliana	core promoter element around -30 of transcription start
PNS9 2495	TATA-box	TATAAATA	Daucus carota	core promoter element around -30 of transcription start
PNS9 2495	TATA-box	TATAAAT	Brassica juncea	core promoter element around -30 of transcription start
PNS9 2495	TATA-box	TATAAA	Helianthus annuus	core promoter element around -30 of transcription start
PNS9 2495	TATA-box	TATAA	Arabidopsis thaliana	core promoter element around -30 of transcription start
PNS9 2495	TATA-box	TATA	Arabidopsis thaliana	core promoter element around -30 of transcription start
PNS9 2495	TATA-box	ATTATA	Brassica napus	core promoter element around -30 of transcription start
PNS9 2495	TATA-box	TATAA	Arabidopsis thaliana	core promoter element around -30 of transcription start
PNS9 2495	TATA-box	TATA	Arabidopsis thaliana	core promoter element around -30 of transcription start
PNS9 2495	TATA-box	TATAAAA	Pisum sativum	core promoter element around -30 of transcription start
PNS9 2495	TATA-box	TATAAA	Helianthus annuus	core promoter element around -30 of transcription start
PNS9 2495	TATA-box	TATAA	Arabidopsis thaliana	core promoter element around -30 of transcription start
PNS9 2495	TATA-box	TATA	Arabidopsis thaliana	core promoter element around -30 of transcription start
PNS9 2495	TATA-box	ATATAA	Brassica oleracea	core promoter element around -30 of transcription start

PNS9 2495	TATA-box	TATA	Arabidopsis thaliana	core promoter element around -30 of transcription start
PNS9 2495	TATA-box	ATATAA	Brassica oleracea	core promoter element around -30 of transcription start
PNS9 2495	TATA-box	TATA	Arabidopsis thaliana	core promoter element around -30 of transcription start
PNS9 2495	TATA-box	TATAAAA	Pisum sativum	core promoter element around -30 of transcription start
PNS9 2495	TATA-box	TATAAA	Helianthus annuus	core promoter element around -30 of transcription start
PNS9 2495	TATA-box	TATAA	Arabidopsis thaliana	core promoter element around -30 of transcription start
PNS9 2495	TATA-box	TATA	Arabidopsis thaliana	core promoter element around -30 of transcription start
PNS9 2495	TATA-box	TATTTAAA	Arabidopsis thaliana	core promoter element around -30 of transcription start
PNS9 2495	TATA-box	TATATTTATATTT	Avena sativa	core promoter element around -30 of transcription start
PNS9 2495	TATA-box	ATATAT	Brassica napus	core promoter element around -30 of transcription start
PNS9 2495	TATA-box	TATA	Arabidopsis thaliana	core promoter element around -30 of transcription start
PNS9 2495	TATA-box	ATATAT	Brassica napus	core promoter element around -30 of transcription start
PNS9 2495	TATA-box	TATA	Arabidopsis thaliana	core promoter element around -30 of transcription start
PNS9 2495	TATA-box	taTATAAAtc	Arabidopsis thaliana	core promoter element around -30 of transcription start
PNS9 2495	TATA-box	TATA	Arabidopsis thaliana	core promoter element around -30 of transcription start
PNS9 2495	TATA-box	TATATTTATATTT	Avena sativa	core promoter element around -30 of transcription start
PNS9 2495	TATA-box	TATTTAAA	Arabidopsis thaliana	core promoter element around -30 of transcription start
PNS9 2495	TATA-box	TACAAAA	Oryza sativa	core promoter element around -30 of transcription start
PNS9 2495	TATA-box	TATACA	Helianthus annuus	core promoter element around -30 of transcription start
PNS9 2495	TATA-box	TATATA	Arabidopsis thaliana	core promoter element around -30 of transcription start
PNS9 2495	TATA-box	TATA	Arabidopsis thaliana	core promoter element around -30 of transcription start
PNS9 2495	TATA-box	TATA	Arabidopsis thaliana	core promoter element around -30 of transcription start

PNS9 2495	TATA-box	TATA	Arabidopsis thaliana	core promoter element around -30 of transcription start
PNS9 2495	TCA-element	CCATCTTTTT	Nicotiana tabacum	cis-acting element involved in salicylic acid responsiveness
PNS9 2495	O2-site	GATGA(C/T)(A/G)TG(A/G)	Zea mays	cis-acting regulatory element involved in zein metabolism regulation
PNS9 2495	STRE	AGGGG	Arabidopsis thaliana	
PNS9 2495	AT-rich element	ATAGAAATCAA	Glycine max	binding site of AT-rich DNA binding protein (ATBP-1)
PNS9 2495	LTR	CCGAAA	Hordeum vulgare	cis-acting element involved in low-temperature responsiveness
PNS9 2495	ARE	AAACCA	Zea mays	cis-acting regulatory element essential for the anaerobic induction
PNS9 2495	AE-box	AGAAACAA	Arabidopsis thaliana	part of a module for light response
PNS9 2495	AAGAA-motif	GAAAGAA	Avena sativa	
PNS9 2495	AAGAA-motif	gGTAAAGAAA	Avena sativa	
PNS9 2495	CAAT-box	CAAT	Nicotiana glutinosa	
PNS9 2495	CAAT-box	CAAT	Nicotiana glutinosa	
PNS9 2495	CAAT-box	CAAT	Nicotiana glutinosa	
PNS9 2495	CAAT-box	CAAAT	Pisum sativum	common cis-acting element in promoter and enhancer regions
PNS9 2495	CAAT-box	CAAAT	Pisum sativum	common cis-acting element in promoter and enhancer regions
PNS9 2495	CAAT-box	CAAAT	Pisum sativum	common cis-acting element in promoter and enhancer regions
PNS9 2495	CAAT-box	CAAAT	Pisum sativum	common cis-acting element in promoter and enhancer regions
PNS9 2495	CAAT-box	CAAAT	Pisum sativum	common cis-acting element in promoter and enhancer regions
PNS9 2495	CAAT-box	CAAAT	Pisum sativum	common cis-acting element in promoter and enhancer regions
PNS9 2495	CAAT-box	CAAT	Nicotiana glutinosa	
PNS9 2495	CAAT-box	CAAAT	Pisum sativum	common cis-acting element in promoter and enhancer regions
PNS9 2495	CAAT-box	CCAAT	Arabidopsis thaliana	common cis-acting element in promoter and enhancer regions

PNS9 2495	CAAT-box	CAAT	Nicotiana glutinosa	
PNS9 2495	CAAT-box	CAAT	Nicotiana glutinosa	
PNS9 2495	CAAT-box	CAAAT	Pisum sativum	common cis-acting element in promoter and enhancer regions
PNS9 2495	CAAT-box	CAAT	Nicotiana glutinosa	
PNS9 2495	CAAT-box	CAAT	Nicotiana glutinosa	
PNS9 2495	CAAT-box	CAAT	Nicotiana glutinosa	
PNS9 2495	CAAT-box	CAAT	Nicotiana glutinosa	
PNS9 2495	CAAT-box	CAAT	Nicotiana glutinosa	
PNS9 2495	CAAT-box	CAAT	Nicotiana glutinosa	
PNS9 2495	CAAT-box	CCAAT	Arabidopsis thaliana	common cis-acting element in promoter and enhancer regions
PNS9 2495	CAAT-box	CAAT	Nicotiana glutinosa	
PNS9 2495	Myb-binding site	CAACAG	Nicotiana tabacum	
PNS9 2495	TCA	TCATCTTCAT	Pisum sativum	
PNS9 2495	Unnamed__4	CTCC	Petroselinum hortense	
PNS9 2495	Unnamed__4	CTCC	Petroselinum hortense	
PNS9 2495	Unnamed__4	CTCC	Petroselinum hortense	
PNS9 2495	Unnamed__4	CTCC	Petroselinum hortense	
PNS9 2495	AT1-motif	AATTATTTTTTATT	Solanum tuberosum	part of a light responsive module
PNS9 2495	MBS	CAACTG	Arabidopsis thaliana	MYB binding site involved in drought-inducibility
PNS9 2495	Myb	TAACTG	Arabidopsis thaliana	
PNS9 2495	Myb	TAACTG	Arabidopsis thaliana	
PNS9 2495	Myb	CAACTG	Arabidopsis thaliana	

PNS9 2495	H-box	CCTACCNNNNNNNCTNNNNA	Phaseolus vulgaris	
PNS9 2495	AT~TATA-box	TATATA	Arabidopsis thaliana	
PNS9 2495	MYB	CAACCA	Arabidopsis thaliana	
PNS9 2495	MYB	TAACCA	Arabidopsis thaliana	
PNS9 2495	MYB	TAACCA	Arabidopsis thaliana	
PNS9 2495	MYB	CAACAG	Arabidopsis thaliana	
PNS9 2495	Box 4	ATTAAT	Petroselinum crispum	part of a conserved DNA module involved in light responsiveness
PNS9 2495	Box 4	ATTAAT	Petroselinum crispum	part of a conserved DNA module involved in light responsiveness
PNS9 2495	Box 4	ATTAAT	Petroselinum crispum	part of a conserved DNA module involved in light responsiveness
PNS9 2495	Box 4	ATTAAT	Petroselinum crispum	part of a conserved DNA module involved in light responsiveness
PNS9 2495	Box 4	ATTAAT	Petroselinum crispum	part of a conserved DNA module involved in light responsiveness
PNS9 2495	Box 4	ATTAAT	Petroselinum crispum	part of a conserved DNA module involved in light responsiveness
PNS9 2495	ERE	ATTTTAAA	Nicotiana glutinos	
PNS9 2495	ERE	ATTTCATA	Nicotiana glutinos	
PNS9 2495	ERE	ATTTTAAA	Nicotiana glutinos	
PNS9 2495		motif_sequence	organism	short_function
PNS9 2495		motif_sequence	organism	short_function
PNS9 2495		motif_sequence	organism	short_function
PNS9 2495		motif_sequence	organism	short_function
PNS9 2495		motif_sequence	organism	short_function
PNS9 2495		motif_sequence	organism	short_function
PNS9 2495		motif_sequence	organism	short_function

PNS9 2495		motif_sequence	organism	short_function
PNS9 2495	TATA	TATAAAAT	Arabidopsis thaliana	
PNS9 2495	TATA	TATAAAAT	Arabidopsis thaliana	
PNS9 2495	TATA	TATAAAAT	Arabidopsis thaliana	
PNS9 2495	MYB-like sequence	TAACCA	Arabidopsis thaliana	
PNS9 2495	MYB-like sequence	TAACCA	Arabidopsis thaliana	
PNS9 2495	AP-1	TGAGTTAG	Arabidopsis thaliana	
PNS9 2540	Myb	TAACTG	Arabidopsis thaliana	
PNS9 2540	Myb	TAACTG	Arabidopsis thaliana	
PNS9 2540	chs-CMA2a	TCACTTGA	Petroselinum crispum	part of a light responsive element
PNS9 2540	AT~TATA-box	TATATA	Arabidopsis thaliana	
PNS9 2540	AT~TATA-box	TATATA	Arabidopsis thaliana	
PNS9 2540	AT~TATA-box	TATATA	Arabidopsis thaliana	
PNS9 2540	AT~TATA-box	TATATA	Arabidopsis thaliana	
PNS9 2540	AT~TATA-box	TATATA	Arabidopsis thaliana	
PNS9 2540	AT~TATA-box	TATATA	Arabidopsis thaliana	
PNS9 2540	Sp1	GGGCGG	Oryza sativa	light responsive element
PNS9 2540	TCCC-motif	TCTCCCT	Spinacia oleracea	part of a light responsive element
PNS9 2540	MYB	TAACCA	Arabidopsis thaliana	
PNS9 2540	MYB	CAACAG	Arabidopsis thaliana	
PNS9 2540	GCN4_motif	TGAGTCA	Oryza sativa	cis-regulatory element involved in endosperm expression
PNS9 2540	GCN4_motif	TGAGTCA	Oryza sativa	cis-regulatory element involved in endosperm expression

PNS92540	Box 4	ATTAAT	Petroselinum crispum	part of a conserved DNA module involved in light responsiveness
PNS92540	Box 4	ATTAAT	Petroselinum crispum	part of a conserved DNA module involved in light responsiveness
PNS92540	Box 4	ATTAAT	Petroselinum crispum	part of a conserved DNA module involved in light responsiveness
PNS92540	Box 4	ATTAAT	Petroselinum crispum	part of a conserved DNA module involved in light responsiveness
PNS92540	Box 4	ATTAAT	Petroselinum crispum	part of a conserved DNA module involved in light responsiveness
PNS92540	Box 4	ATTAAT	Petroselinum crispum	part of a conserved DNA module involved in light responsiveness
PNS92540	Box 4	ATTAAT	Petroselinum crispum	part of a conserved DNA module involved in light responsiveness
PNS92540	Box 4	ATTAAT	Petroselinum crispum	part of a conserved DNA module involved in light responsiveness
PNS92540	ERE	ATTTTAAA	Nicotiana glutinos	
PNS92540	ERE	ATTTTAAA	Nicotiana glutinos	
PNS92540		motif_sequence	organism	short_function
PNS92540		motif_sequence	organism	short_function
PNS92540		motif_sequence	organism	short_function
PNS92540		motif_sequence	organism	short_function
PNS92540		motif_sequence	organism	short_function
PNS92540	TATA	TATAAAAT	Arabidopsis thaliana	
PNS92540	MYB-like sequence	TAACCA	Arabidopsis thaliana	
PNS92540	TATA-box	TATATA	Arabidopsis thaliana	core promoter element around -30 of transcription start
PNS92540	TATA-box	TATA	Arabidopsis thaliana	core promoter element around -30 of transcription start
PNS92540	TATA-box	TATAAGAA	Zea mays	core promoter element around -30 of transcription start
PNS92540	TATA-box	TATATAA	Arabidopsis thaliana	core promoter element around -30 of transcription start
PNS92540	TATA-box	TATATA	Arabidopsis thaliana	core promoter element around -30 of transcription start

PNS9 2540	TATA-box	TATA	Arabidopsis thaliana	core promoter element around -30 of transcription start
PNS9 2540	TATA-box	TATATTTATATT T	Avena sativa	core promoter element around -30 of transcription start
PNS9 2540	TATA-box	TATAAA	Helianthus annuus	core promoter element around -30 of transcription start
PNS9 2540	TATA-box	TATAA	Arabidopsis thaliana	core promoter element around -30 of transcription start
PNS9 2540	TATA-box	TATA	Arabidopsis thaliana	core promoter element around -30 of transcription start
PNS9 2540	TATA-box	TATA	Arabidopsis thaliana	core promoter element around -30 of transcription start
PNS9 2540	TATA-box	TATAA	Arabidopsis thaliana	core promoter element around -30 of transcription start
PNS9 2540	TATA-box	TATA	Arabidopsis thaliana	core promoter element around -30 of transcription start
PNS9 2540	TATA-box	TATACA	Helianthus annuus	core promoter element around -30 of transcription start
PNS9 2540	TATA-box	TATA	Arabidopsis thaliana	core promoter element around -30 of transcription start
PNS9 2540	TATA-box	ATTATA	Brassica napus	core promoter element around -30 of transcription start
PNS9 2540	TATA-box	TATAA	Arabidopsis thaliana	core promoter element around -30 of transcription start
PNS9 2540	TATA-box	TATA	Arabidopsis thaliana	core promoter element around -30 of transcription start
PNS9 2540	TATA-box	ATTATA	Brassica napus	core promoter element around -30 of transcription start
PNS9 2540	TATA-box	TATAA	Arabidopsis thaliana	core promoter element around -30 of transcription start
PNS9 2540	TATA-box	TATA	Arabidopsis thaliana	core promoter element around -30 of transcription start
PNS9 2540	TATA-box	ccTATAAAaa	Arabidopsis thaliana	core promoter element around -30 of transcription start
PNS9 2540	TATA-box	ATATAA	Brassica oleracea	core promoter element around -30 of transcription start
PNS9 2540	TATA-box	TATA	Arabidopsis thaliana	core promoter element around -30 of transcription start
PNS9 2540	TATA-box	TATAAAA	Pisum sativum	core promoter element around -30 of transcription start
PNS9 2540	TATA-box	TATAAA	Helianthus annuus	core promoter element around -30 of transcription start
PNS9 2540	TATA-box	TATAA	Arabidopsis thaliana	core promoter element around -30 of transcription start

PNS9 2540	TATA-box	TATA	Arabidopsis thaliana	core promoter element around -30 of transcription start
PNS9 2540	TATA-box	ccTATAAAaa	Arabidopsis thaliana	core promoter element around -30 of transcription start
PNS9 2540	TATA-box	TATA	Arabidopsis thaliana	core promoter element around -30 of transcription start
PNS9 2540	TATA-box	TATATTTATATT T	Avena sativa	core promoter element around -30 of transcription start
PNS9 2540	TATA-box	ATATAA	Brassica oleracea	core promoter element around -30 of transcription start
PNS9 2540	TATA-box	TATA	Arabidopsis thaliana	core promoter element around -30 of transcription start
PNS9 2540	TATA-box	ATATAA	Brassica oleracea	core promoter element around -30 of transcription start
PNS9 2540	TATA-box	TATA	Arabidopsis thaliana	core promoter element around -30 of transcription start
PNS9 2540	TATA-box	ATATAT	Brassica napus	core promoter element around -30 of transcription start
PNS9 2540	TATA-box	TATATA	Arabidopsis thaliana	core promoter element around -30 of transcription start
PNS9 2540	TATA-box	ATATAT	Brassica napus	core promoter element around -30 of transcription start
PNS9 2540	TATA-box	TATATA	Arabidopsis thaliana	core promoter element around -30 of transcription start
PNS9 2540	TATA-box	TATA	Arabidopsis thaliana	core promoter element around -30 of transcription start
PNS9 2540	TATA-box	TATA	Arabidopsis thaliana	core promoter element around -30 of transcription start
PNS9 2540	TATA-box	TATATAA	Arabidopsis thaliana	core promoter element around -30 of transcription start
PNS9 2540	TATA-box	TATATA	Arabidopsis thaliana	core promoter element around -30 of transcription start
PNS9 2540	TATA-box	TATA	Arabidopsis thaliana	core promoter element around -30 of transcription start
PNS9 2540	TATA-box	ATATAT	Brassica napus	core promoter element around -30 of transcription start
PNS9 2540	TATA-box	TATATA	Arabidopsis thaliana	core promoter element around -30 of transcription start
PNS9 2540	TATA-box	TATA	Arabidopsis thaliana	core promoter element around -30 of transcription start
PNS9 2540	TATA-box	ATTATA	Brassica napus	core promoter element around -30 of transcription start
PNS9 2540	TATA-box	TATAA	Arabidopsis thaliana	core promoter element around -30 of transcription start

PNS9 2540	TATA-box	TATA	Arabidopsis thaliana	core promoter element around -30 of transcription start
PNS9 2540	TATA-box	TATAA	Arabidopsis thaliana	core promoter element around -30 of transcription start
PNS9 2540	TATA-box	TATA	Arabidopsis thaliana	core promoter element around -30 of transcription start
PNS9 2540	TATA-box	TATA	Arabidopsis thaliana	core promoter element around -30 of transcription start
PNS9 2540	TCT-motif	TCTTAC	Arabidopsis thaliana	part of a light responsive element
PNS9 2540	STRE	AGGGG	Arabidopsis thaliana	
PNS9 2540	STRE	AGGGG	Arabidopsis thaliana	
PNS9 2540	3-AF1 binding site	TAAGAGAGGAA	Solanum tuberosum	light responsive element
PNS9 2540	AT-rich element	ATAGAAATCAA	Glycine max	binding site of AT-rich DNA binding protein (ATBP-1)
PNS9 2540	AT-rich element	ATAGAAATCAA	Glycine max	binding site of AT-rich DNA binding protein (ATBP-1)
PNS9 2540	GARE-motif	TCTGTTG	Brassica oleracea	gibberellin-responsive element
PNS9 2540	MBSI	TTTTTACGGTTA	Petunia hybrida	MYB binding site involved in flavonoid biosynthetic genes regulation
PNS9 2540	G-Box	CACGTT	Pisum sativum	cis-acting regulatory element involved in light responsiveness
PNS9 2540	TATC-box	TATCCCA	Oryza sativa	cis-acting element involved in gibberellin-responsiveness
PNS9 2540	AE-box	AGAAACAA	Arabidopsis thaliana	part of a module for light response
PNS9 2540	AAGAA-motif	GAAAGAA	Avena sativa	
PNS9 2540	Myb-binding site	CAACAG	Nicotiana tabacum	
PNS9 2540	CAAT-box	CAAAT	Pisum sativum	common cis-acting element in promoter and enhancer regions
PNS9 2540	CAAT-box	CAAAT	Pisum sativum	common cis-acting element in promoter and enhancer regions
PNS9 2540	CAAT-box	CAAT	Nicotiana glutinosa	
PNS9 2540	CAAT-box	CAAT	Nicotiana glutinosa	

PNS9 2540	CAAT-box	CAAT	Nicotiana glutinosa	
PNS9 2540	CAAT-box	CAAT	Nicotiana glutinosa	
PNS9 2540	CAAT-box	CAAT	Nicotiana glutinosa	
PNS9 2540	CAAT-box	CAAAT	Pisum sativum	common cis-acting element in promoter and enhancer regions
PNS9 2540	CAAT-box	CAAT	Nicotiana glutinosa	
PNS9 2540	CAAT-box	CAAT	Nicotiana glutinosa	
PNS9 2540	CAAT-box	CAAT	Nicotiana glutinosa	
PNS9 2540	CAAT-box	CAAAT	Pisum sativum	common cis-acting element in promoter and enhancer regions
PNS9 2540	CAAT-box	CAAAT	Pisum sativum	common cis-acting element in promoter and enhancer regions
PNS9 2540	CAAT-box	CCAAT	Arabidopsis thaliana	common cis-acting element in promoter and enhancer regions
PNS9 2540	CAAT-box	CAAT	Nicotiana glutinosa	
PNS9 2540	CAAT-box	CAAAT	Pisum sativum	common cis-acting element in promoter and enhancer regions
PNS9 2540	CAAT-box	CCAAT	Arabidopsis thaliana	common cis-acting element in promoter and enhancer regions
PNS9 2540	CAAT-box	CAAT	Nicotiana glutinosa	
PNS9 2540	CAAT-box	CCAAT	Arabidopsis thaliana	common cis-acting element in promoter and enhancer regions
PNS9 2540	CAAT-box	CAAT	Nicotiana glutinosa	
PNS9 2540	CAAT-box	CAAT	Nicotiana glutinosa	
PNS9 2540	CAAT-box	CCAAT	Arabidopsis thaliana	common cis-acting element in promoter and enhancer regions
PNS9 2540	CAAT-box	CAAT	Nicotiana glutinosa	
PNS9 2540	CAAT-box	CAAT	Nicotiana glutinosa	
PNS9 2540	CAAT-box	CAAAT	Pisum sativum	common cis-acting element in promoter and enhancer regions
PNS9 2540	CAAT-box	CCAAT	Arabidopsis thaliana	common cis-acting element in promoter and enhancer regions

PNS92540	CAAT-box	CAAT	Nicotiana glutinosa	
PNS92540	WUN-motif	AAATTACTA	Nicotiana glutinosa	
PNS92540	WUN-motif	AAATTACT	Nicotiana glutinosa	
PNS92540	ABRE	ACGTG	Arabidopsis thaliana	cis-acting element involved in the abscisic acid responsiveness
PNS92540	MYC	CAATTG	Arabidopsis thaliana	
PNS92540	MYC	CATGTG	Arabidopsis thaliana	
PNS92540	MYC	CATTTG	Arabidopsis thaliana	
PNS92540	Unnamed__4	CTCC	Petroselinum hortense	
PNS92540	Unnamed__4	CTCC	Petroselinum hortense	
PNS92540	Unnamed__4	CTCC	Petroselinum hortense	
PNS92540	Unnamed__4	CTCC	Petroselinum hortense	
PNS92540	Unnamed__4	CTCC	Petroselinum hortense	
PNS92540	Unnamed__4	CTCC	Petroselinum hortense	
PNS92540	Unnamed__4	CTCC	Petroselinum hortense	
PNS92540	Unnamed__4	CTCC	Petroselinum hortense	
PNS92540	Unnamed__4	CTCC	Petroselinum hortense	
PNS92540	AT1-motif	AATTATTTTTTATT	Solanum tuberosum	part of a light responsive module
PNS92540	TCA	TCATCTTCAT	Pisum sativum	
PNS92540	TCA	TCATCTTCAT	Pisum sativum	
PNS98364	G-Box	CACGTT	Pisum sativum	cis-acting regulatory element involved in light responsiveness
PNS98364	G-Box	CACGTT	Pisum sativum	cis-acting regulatory element involved in light responsiveness
PNS98364	ARE	AAACCA	Zea mays	cis-acting regulatory element essential for the anaerobic induction

PNS9 8364	Myb-binding site	CAACAG	Nicotiana tabacum	
PNS9 8364	CAAT-box	CAAT	Nicotiana glutinosa	
PNS9 8364	CAAT-box	CCAAT	Arabidopsis thaliana	common cis-acting element in promoter and enhancer regions
PNS9 8364	CAAT-box	CCAAT	Arabidopsis thaliana	common cis-acting element in promoter and enhancer regions
PNS9 8364	CAAT-box	CAAT	Nicotiana glutinosa	
PNS9 8364	CAAT-box	CCAAT	Arabidopsis thaliana	common cis-acting element in promoter and enhancer regions
PNS9 8364	CAAT-box	CCAAT	Arabidopsis thaliana	common cis-acting element in promoter and enhancer regions
PNS9 8364	CAAT-box	CAAT	Nicotiana glutinosa	
PNS9 8364	CAAT-box	CAAAT	Pisum sativum	common cis-acting element in promoter and enhancer regions
PNS9 8364	CAAT-box	CCAAT	Arabidopsis thaliana	common cis-acting element in promoter and enhancer regions
PNS9 8364	CAAT-box	CAAT	Nicotiana glutinosa	
PNS9 8364	CAAT-box	CAAT	Nicotiana glutinosa	
PNS9 8364	CAAT-box	CAAT	Nicotiana glutinosa	
PNS9 8364	CAAT-box	CAAAT	Pisum sativum	common cis-acting element in promoter and enhancer regions
PNS9 8364	CAAT-box	CAAAT	Pisum sativum	common cis-acting element in promoter and enhancer regions
PNS9 8364	CAAT-box	CAAT	Nicotiana glutinosa	
PNS9 8364	CAAT-box	CAAT	Nicotiana glutinosa	
PNS9 8364	CAAT-box	CCAAT	Arabidopsis thaliana	common cis-acting element in promoter and enhancer regions
PNS9 8364	CAAT-box	CAAAT	Pisum sativum	common cis-acting element in promoter and enhancer regions
PNS9 8364	CAAT-box	CAAAT	Pisum sativum	common cis-acting element in promoter and enhancer regions
PNS9 8364	CAAT-box	CAAAT	Pisum sativum	common cis-acting element in promoter and enhancer regions
PNS9 8364	CAAT-box	CAAAT	Pisum sativum	common cis-acting element in promoter and enhancer regions

PNS98364	CAAT-box	CAAAT	Pisum sativum	common cis-acting element in promoter and enhancer regions
PNS98364	CAAT-box	CAAT	Nicotiana glutinosa	
PNS98364	CAAT-box	CCAAT	Arabidopsis thaliana	common cis-acting element in promoter and enhancer regions
PNS98364	CAAT-box	CAAT	Nicotiana glutinosa	
PNS98364	CAAT-box	CCAAT	Arabidopsis thaliana	common cis-acting element in promoter and enhancer regions
PNS98364	CAAT-box	CAAT	Nicotiana glutinosa	
PNS98364	CAAT-box	CAAAT	Pisum sativum	common cis-acting element in promoter and enhancer regions
PNS98364	AT1-motif	AATTATTTTTTATT	Solanum tuberosum	part of a light responsive module
PNS98364	Unnamed__4	CTCC	Petroselinum hortense	
PNS98364	Unnamed__4	CTCC	Petroselinum hortense	
PNS98364	Unnamed__4	CTCC	Petroselinum hortense	
PNS98364	Unnamed__4	CTCC	Petroselinum hortense	
PNS98364	Unnamed__4	CTCC	Petroselinum hortense	
PNS98364	Unnamed__4	CTCC	Petroselinum hortense	
PNS98364	Unnamed__4	CTCC	Petroselinum hortense	
PNS98364	MYC	CATGTG	Arabidopsis thaliana	
PNS98364	MYC	CAATTG	Arabidopsis thaliana	
PNS98364	ABRE	ACGTG	Arabidopsis thaliana	cis-acting element involved in the abscisic acid responsiveness
PNS98364	ABRE	ACGTG	Arabidopsis thaliana	cis-acting element involved in the abscisic acid responsiveness
PNS98364	ABRE	ACGTG	Arabidopsis thaliana	cis-acting element involved in the abscisic acid responsiveness
PNS98364	AuxRR-core	GGTCCAT	Nicotiana tabacum	cis-acting regulatory element involved in auxin responsiveness
PNS98364	TATA-box	ATATAT	Brassica napus	core promoter element around -30 of transcription start

PNS98364	TATA-box	TATA	Arabidopsis thaliana	core promoter element around -30 of transcription start
PNS98364	TATA-box	TATA	Arabidopsis thaliana	core promoter element around -30 of transcription start
PNS98364	TATA-box	TACAAAA	Oryza sativa	core promoter element around -30 of transcription start
PNS98364	TATA-box	ATATAA	Brassica oleracea	core promoter element around -30 of transcription start
PNS98364	TATA-box	TATA	Arabidopsis thaliana	core promoter element around -30 of transcription start
PNS98364	TATA-box	TAAAGATT	Arabidopsis thaliana	core promoter element around -30 of transcription start
PNS98364	TATA-box	ATATAA	Brassica oleracea	core promoter element around -30 of transcription start
PNS98364	TATA-box	TATA	Arabidopsis thaliana	core promoter element around -30 of transcription start
PNS98364	TATA-box	TATA	Arabidopsis thaliana	core promoter element around -30 of transcription start
PNS98364	TATA-box	taTATAAAtc	Arabidopsis thaliana	core promoter element around -30 of transcription start
PNS98364	TATA-box	TATAAAT	Brassica juncea	core promoter element around -30 of transcription start
PNS98364	TATA-box	TATAAA	Helianthus annuus	core promoter element around -30 of transcription start
PNS98364	TATA-box	TATATAA	Arabidopsis thaliana	core promoter element around -30 of transcription start
PNS98364	TATA-box	TATATA	Arabidopsis thaliana	core promoter element around -30 of transcription start
PNS98364	TATA-box	ATATAA	Brassica oleracea	core promoter element around -30 of transcription start
PNS98364	TATA-box	TATA	Arabidopsis thaliana	core promoter element around -30 of transcription start
PNS98364	TATA-box	ATTATA	Brassica napus	core promoter element around -30 of transcription start
PNS98364	TATA-box	TATATAA	Arabidopsis thaliana	core promoter element around -30 of transcription start
PNS98364	TATA-box	TATATA	Arabidopsis thaliana	core promoter element around -30 of transcription start
PNS98364	TATA-box	TATA	Arabidopsis thaliana	core promoter element around -30 of transcription start
PNS98364	TATA-box	ATTATA	Brassica napus	core promoter element around -30 of transcription start
PNS98364	TATA-box	TATAA	Arabidopsis thaliana	core promoter element around -30 of transcription start

PNS9 8364	TATA-box	TATA	Arabidopsis thaliana	core promoter element around -30 of transcription start
PNS9 8364	TATA-box	TATA	Arabidopsis thaliana	core promoter element around -30 of transcription start
PNS9 8364	TATA-box	ATATAA	Brassica oleracea	core promoter element around -30 of transcription start
PNS9 8364	TATA-box	TATA	Arabidopsis thaliana	core promoter element around -30 of transcription start
PNS9 8364	TATA-box	TATAAA	Helianthus annuus	core promoter element around -30 of transcription start
PNS9 8364	TATA-box	TATAA	Arabidopsis thaliana	core promoter element around -30 of transcription start
PNS9 8364	TATA-box	TATA	Arabidopsis thaliana	core promoter element around -30 of transcription start
PNS9 8364	TATA-box	TATA	Arabidopsis thaliana	core promoter element around -30 of transcription start
PNS9 8364	TATA-box	ATATAA	Brassica oleracea	core promoter element around -30 of transcription start
PNS9 8364	TATA-box	TATA	Arabidopsis thaliana	core promoter element around -30 of transcription start
PNS9 8364	TATA-box	ATATAA	Brassica oleracea	core promoter element around -30 of transcription start
PNS9 8364	TATA-box	TATA	Arabidopsis thaliana	core promoter element around -30 of transcription start
PNS9 8364	TATA-box	ATATAA	Brassica oleracea	core promoter element around -30 of transcription start
PNS9 8364	TATA-box	TATA	Arabidopsis thaliana	core promoter element around -30 of transcription start
PNS9 8364	TATA-box	TATA	Arabidopsis thaliana	core promoter element around -30 of transcription start
PNS9 8364	TATA-box	TATAAAA	Pisum sativum	core promoter element around -30 of transcription start
PNS9 8364	TATA-box	TATAAA	Helianthus annuus	core promoter element around -30 of transcription start
PNS9 8364	TATA-box	TATAA	Arabidopsis thaliana	core promoter element around -30 of transcription start
PNS9 8364	TATA-box	TATA	Arabidopsis thaliana	core promoter element around -30 of transcription start
PNS9 8364	TATA-box	ATATAA	Brassica oleracea	core promoter element around -30 of transcription start
PNS9 8364	TATA-box	TATA	Arabidopsis thaliana	core promoter element around -30 of transcription start
PNS9 8364	TATA-box	ATATAT	Brassica napus	core promoter element around -30 of transcription start

PNS9 8364	TATA-box	TATATA	Arabidopsis thaliana	core promoter element around -30 of transcription start
PNS9 8364	TATA-box	TATA	Arabidopsis thaliana	core promoter element around -30 of transcription start
PNS9 8364	TATA-box	TATAA	Arabidopsis thaliana	core promoter element around -30 of transcription start
PNS9 8364	TATA-box	TATA	Arabidopsis thaliana	core promoter element around -30 of transcription start
PNS9 8364	TATA-box	ATATAT	Brassica napus	core promoter element around -30 of transcription start
PNS9 8364	TATA-box	TATATTTATATT T	Avena sativa	core promoter element around -30 of transcription start
PNS9 8364	TATA-box	ATTATA	Brassica napus	core promoter element around -30 of transcription start
PNS9 8364	TATA-box	TATAA	Arabidopsis thaliana	core promoter element around -30 of transcription start
PNS9 8364	TATA-box	TATA	Arabidopsis thaliana	core promoter element around -30 of transcription start
PNS9 8364	TATA-box	ATTATA	Brassica napus	core promoter element around -30 of transcription start
PNS9 8364	TATA-box	TATAA	Arabidopsis thaliana	core promoter element around -30 of transcription start
PNS9 8364	TATA-box	TATA	Arabidopsis thaliana	core promoter element around -30 of transcription start
PNS9 8364	TATA-box	ccTATAAAaa	Arabidopsis thaliana	core promoter element around -30 of transcription start
PNS9 8364	TATA-box	TATA	Arabidopsis thaliana	core promoter element around -30 of transcription start
PNS9 8364	Unnamed__1	CGTGG	Zea mays	
PNS9 8364	Unnamed__1	CGTGG	Zea mays	
PNS9 8364	Unnamed__1	CGTGG	Zea mays	
PNS9 8364	CGTCA-motif	CGTCA	Hordeum vulgare	cis-acting regulatory element involved in the MeJA-responsiveness
PNS9 8364	CGTCA-motif	CGTCA	Hordeum vulgare	cis-acting regulatory element involved in the MeJA-responsiveness
PNS9 8364	as-1	TGACG	Arabidopsis thaliana	
PNS9 8364	as-1	TGACG	Arabidopsis thaliana	
PNS9 8364	TCCC-motif	TCTCCCT	Spinacia oleracea	part of a light responsive element

PNS98364	MYB	CAACCA	Arabidopsis thaliana	
PNS98364	MYB	CAACAG	Arabidopsis thaliana	
PNS98364	MYB	TAACCA	Arabidopsis thaliana	
PNS98364	MYB	TAACCA	Arabidopsis thaliana	
PNS98364	MYB	CAACCA	Arabidopsis thaliana	
PNS98364	ERE	ATTTCATA	Nicotiana glutinos	
PNS98364	ERE	ATTTTAAA	Nicotiana glutinos	
PNS98364	ERE	ATTTTAAA	Nicotiana glutinos	
PNS98364	ERE	ATTTTAAA	Nicotiana glutinos	
PNS98364		motif_sequence	organism	short_function
PNS98364		motif_sequence	organism	short_function
PNS98364		motif_sequence	organism	short_function
PNS98364		motif_sequence	organism	short_function
PNS98364		motif_sequence	organism	short_function
PNS98364	TGACG-motif	TGACG	Hordeum vulgare	cis-acting regulatory element involved in the MeJA-responsiveness
PNS98364	TGACG-motif	TGACG	Hordeum vulgare	cis-acting regulatory element involved in the MeJA-responsiveness
PNS98364	TGA-element	AACGAC	Brassica oleracea	auxin-responsive element
PNS98364	TATA	TATAAAAT	Arabidopsis thaliana	
PNS98364	TATA	TATAAAAT	Arabidopsis thaliana	
PNS98364	WRE3	CCACCT	Pisum sativum	
PNS98364	MYB-like sequence	TAACCA	Arabidopsis thaliana	
PNS98364	MYB-like sequence	TAACCA	Arabidopsis thaliana	

PNS98364	G-box	CACGTC	Zea mays	cis-acting regulatory element involved in light responsiveness
PNS98364	G-box	CACGAC	Zea mays	cis-acting regulatory element involved in light responsiveness
PNS98364	I-box	cCATATCCAAT	Flaveria trinervia	part of a light responsive element
PNS98364	GT1-motif	GGTTAAT	Avena sativa	light responsive element
PNS98364	GT1-motif	GGTTAA	Arabidopsis thaliana	light responsive element
PNS98364	AT~TATA-box	TATATAAA	Arabidopsis thaliana	
PNS98364	AT~TATA-box	TATATA	Arabidopsis thaliana	
PNS98364	AT~TATA-box	TATATA	Arabidopsis thaliana	
PNS98364	AT~TATA-box	TATATA	Arabidopsis thaliana	
PNT02530	dOCT	CTCGGATC	Zea mays	
PNT02530	Myb	TAACTG	Arabidopsis thaliana	
PNT02530	Myb	TAACTG	Arabidopsis thaliana	
PNT02530	P-box	CCTTTTG	Oryza sativa	gibberellin-responsive element
PNT02530	HD-Zip 1	CAAT(A/T)ATTG	Arabidopsis thaliana	element involved in differentiation of the palisade mesophyll cells
PNT02530	GT1-motif	GGTTAA	Arabidopsis thaliana	light responsive element
PNT02530	AT~TATA-box	TATATA	Arabidopsis thaliana	
PNT02530	AT~TATA-box	TATATA	Arabidopsis thaliana	
PNT02530	AT~TATA-box	TATATA	Arabidopsis thaliana	
PNT02530	AT~TATA-box	TATATA	Arabidopsis thaliana	
PNT02530	MYB	CAACAG	Arabidopsis thaliana	
PNT02530	ERE	ATTTTAAA	Nicotiana glutinos	
PNT02530	ERE	ATTTTAAA	Nicotiana glutinos	

PNT02530		motif_sequence	organism	short_function
PNT02530		motif_sequence	organism	short_function
PNT02530		motif_sequence	organism	short_function
PNT02530		motif_sequence	organism	short_function
PNT02530		motif_sequence	organism	short_function
PNT02530		motif_sequence	organism	short_function
PNT02530		motif_sequence	organism	short_function
PNT02530		motif_sequence	organism	short_function
PNT02530		motif_sequence	organism	short_function
PNT02530	Box 4	ATTAAT	Petroselinum crispum	part of a conserved DNA module involved in light responsiveness
PNT02530	WRE3	CCACCT	Pisum sativum	
PNT02530	G-box	CACGAC	Zea mays	cis-acting regulatory element involved in light responsiveness
PNT02530	TATA-box	TATTTAAA	Arabidopsis thaliana	core promoter element around -30 of transcription start
PNT02530	TATA-box	TATAAA	Helianthus annuus	core promoter element around -30 of transcription start
PNT02530	TATA-box	TATAA	Arabidopsis thaliana	core promoter element around -30 of transcription start
PNT02530	TATA-box	TATA	Arabidopsis thaliana	core promoter element around -30 of transcription start
PNT02530	TATA-box	TATATA	Arabidopsis thaliana	core promoter element around -30 of transcription start
PNT02530	TATA-box	ATATAT	Brassica napus	core promoter element around -30 of transcription start
PNT02530	TATA-box	TATATA	Arabidopsis thaliana	core promoter element around -30 of transcription start
PNT02530	TATA-box	ATATAT	Brassica napus	core promoter element around -30 of transcription start
PNT02530	TATA-box	TATATA	Arabidopsis thaliana	core promoter element around -30 of transcription start
PNT02530	TATA-box	ATATAT	Brassica napus	core promoter element around -30 of transcription start

PNT0 2530	TATA-box	TATA	Arabidopsis thaliana	core promoter element around -30 of transcription start
PNT0 2530	TATA-box	TACAAAA	Oryza sativa	core promoter element around -30 of transcription start
PNT0 2530	TATA-box	ATATAA	Brassica oleracea	core promoter element around -30 of transcription start
PNT0 2530	TATA-box	TATA	Arabidopsis thaliana	core promoter element around -30 of transcription start
PNT0 2530	TATA-box	ATATAT	Brassica napus	core promoter element around -30 of transcription start
PNT0 2530	TATA-box	TATA	Arabidopsis thaliana	core promoter element around -30 of transcription start
PNT0 2530	TATA-box	ATATAT	Brassica napus	core promoter element around -30 of transcription start
PNT0 2530	TATA-box	TATATA	Arabidopsis thaliana	core promoter element around -30 of transcription start
PNT0 2530	TATA-box	TATA	Arabidopsis thaliana	core promoter element around -30 of transcription start
PNT0 2530	TATA-box	TACAAAA	Oryza sativa	core promoter element around -30 of transcription start
PNT0 2530	TCT-motif	TCTTAC	Arabidopsis thaliana	part of a light responsive element
PNT0 2530	STRE	AGGGG	Arabidopsis thaliana	
PNT0 2530	O2-site	GATGATGTGG	Zea mays	cis-acting regulatory element involved in zein metabolism regulation
PNT0 2530	GARE-motif	TCTGTTG	Brassica oleracea	gibberellin-responsive element
PNT0 2530	AE-box	AGAAACAA	Arabidopsis thaliana	part of a module for light response
PNT0 2530	ARE	AAACCA	Zea mays	cis-acting regulatory element essential for the anaerobic induction
PNT0 2530	CAAT-box	CCAAT	Arabidopsis thaliana	common cis-acting element in promoter and enhancer regions
PNT0 2530	CAAT-box	CAAT	Nicotiana glutinosa	
PNT0 2530	CAAT-box	CAAT	Nicotiana glutinosa	
PNT0 2530	CAAT-box	CCAAT	Arabidopsis thaliana	common cis-acting element in promoter and enhancer regions
PNT0 2530	CAAT-box	CAAT	Nicotiana glutinosa	
PNT0 2530	CAAT-box	CAAAT	Pisum sativum	common cis-acting element in promoter and enhancer regions

PNTO 2530	CAAT-box	CAAT	Nicotiana glutinosa	
PNTO 2530	CAAT-box	CAAT	Nicotiana glutinosa	
PNTO 2530	CAAT-box	CAAT	Nicotiana glutinosa	
PNTO 2530	CAAT-box	CAAT	Nicotiana glutinosa	
PNTO 2530	CAAT-box	CCAAT	Arabidopsis thaliana	common cis-acting element in promoter and enhancer regions
PNTO 2530	CAAT-box	CAAT	Nicotiana glutinosa	
PNTO 2530	CAAT-box	CCAAT	Arabidopsis thaliana	common cis-acting element in promoter and enhancer regions
PNTO 2530	CAAT-box	CAAT	Nicotiana glutinosa	
PNTO 2530	CAAT-box	CAAT	Nicotiana glutinosa	
PNTO 2530	CAAT-box	CAAT	Nicotiana glutinosa	
PNTO 2530	CAAT-box	CAAT	Nicotiana glutinosa	
PNTO 2530	CAAT-box	CAAAT	Pisum sativum	common cis-acting element in promoter and enhancer regions
PNTO 2530	CAAT-box	CCAAT	Arabidopsis thaliana	common cis-acting element in promoter and enhancer regions
PNTO 2530	CAAT-box	CAAAT	Pisum sativum	common cis-acting element in promoter and enhancer regions
PNTO 2530	CAAT-box	CCAAT	Arabidopsis thaliana	common cis-acting element in promoter and enhancer regions
PNTO 2530	CAAT-box	CAAT	Nicotiana glutinosa	
PNTO 2530	CAAT-box	CAAT	Nicotiana glutinosa	
PNTO 2530	CAAT-box	CAAAT	Pisum sativum	common cis-acting element in promoter and enhancer regions
PNTO 2530	CAAT-box	CAAT	Nicotiana glutinosa	
PNTO 2530	CAAT-box	CAAAT	Pisum sativum	common cis-acting element in promoter and enhancer regions
PNTO 2530	CAAT-box	CAAAT	Pisum sativum	common cis-acting element in promoter and enhancer regions
PNTO 2530	CAAT-box	CAAT	Nicotiana glutinosa	

PNTO 2530	CAAT-box	CAAAT	Pisum sativum	common cis-acting element in promoter and enhancer regions
PNTO 2530	CAAT-box	CAAT	Nicotiana glutinosa	
PNTO 2530	CAAT-box	CAAAT	Pisum sativum	common cis-acting element in promoter and enhancer regions
PNTO 2530	CAAT-box	CAAT	Nicotiana glutinosa	
PNTO 2530	CAAT-box	CAAT	Nicotiana glutinosa	
PNTO 2530	CAAT-box	CAAT	Nicotiana glutinosa	
PNTO 2530	CAAT-box	CCCAATTT	Arabidopsis thaliana	common cis-acting element in promoter and enhancer regions
PNTO 2530	CAAT-box	CCAAT	Arabidopsis thaliana	common cis-acting element in promoter and enhancer regions
PNTO 2530	CAAT-box	CAAT	Nicotiana glutinosa	
PNTO 2530	CAAT-box	CAAT	Nicotiana glutinosa	
PNTO 2530	Myb-binding site	CAACAG	Nicotiana tabacum	
PNTO 2530	WUN-motif	CAATTACAT	Nicotiana glutinosa	
PNTO 2530	WUN-motif	AAATTACTA	Nicotiana glutinosa	
PNTO 2530	Unnamed__4	CTCC	Petroselinum hortense	
PNTO 2530	Unnamed__4	CTCC	Petroselinum hortense	
PNTO 2530	Unnamed__4	CTCC	Petroselinum hortense	
PNTO 2530	MYC	CAATTG	Arabidopsis thaliana	
PNTO 2530	AT1-motif	AATTATTTTTTATT	Solanum tuberosum	part of a light responsive module
PNTO 5527	MRE	AACCTAA	Petroselinum crispum	MYB binding site involved in light responsiveness
PNTO 5527	MRE	AACCTAA	Petroselinum crispum	MYB binding site involved in light responsiveness
PNTO 5527	Unnamed__2	AACCTAACCT	Petroselinum hortense	
PNTO 5527	Unnamed__2	AACCTAACCT	Petroselinum hortense	

PNTO5527	ARE	AAACCA	Zea mays	cis-acting regulatory element essential for the anaerobic induction
PNTO5527	ARE	AAACCA	Zea mays	cis-acting regulatory element essential for the anaerobic induction
PNTO5527	ARE	AAACCA	Zea mays	cis-acting regulatory element essential for the anaerobic induction
PNTO5527	ARE	AAACCA	Zea mays	cis-acting regulatory element essential for the anaerobic induction
PNTO5527	ARE	AAACCA	Zea mays	cis-acting regulatory element essential for the anaerobic induction
PNTO5527	ARE	AAACCA	Zea mays	cis-acting regulatory element essential for the anaerobic induction
PNTO5527	ARE	AAACCA	Zea mays	cis-acting regulatory element essential for the anaerobic induction
PNTO5527	ARE	AAACCA	Zea mays	cis-acting regulatory element essential for the anaerobic induction
PNTO5527	ARE	AAACCA	Zea mays	cis-acting regulatory element essential for the anaerobic induction
PNTO5527	MYC	CAATTG	Arabidopsis thaliana	
PNTO5527	TCA	TCATCTTCAT	Pisum sativum	
PNTO5527	Unnamed__4	CTCC	Petroselinum hortense	
PNTO5527	WUN-motif	AAATTACTA	Nicotiana glutinosa	
PNTO5527	CAAT-box	CAAAT	Pisum sativum	common cis-acting element in promoter and enhancer regions
PNTO5527	CAAT-box	CAAAT	Pisum sativum	common cis-acting element in promoter and enhancer regions
PNTO5527	CAAT-box	CAAAT	Pisum sativum	common cis-acting element in promoter and enhancer regions
PNTO5527	CAAT-box	CAAT	Nicotiana glutinosa	
PNTO5527	CAAT-box	CAAT	Nicotiana glutinosa	
PNTO5527	CAAT-box	CAAAT	Pisum sativum	common cis-acting element in promoter and enhancer regions
PNTO5527	CAAT-box	CAAT	Nicotiana glutinosa	
PNTO5527	CAAT-box	CCAAT	Arabidopsis thaliana	common cis-acting element in promoter and enhancer regions
PNTO5527	CAAT-box	CAAT	Nicotiana glutinosa	

PNTO 5527	CAAT-box	CAAT	Nicotiana glutinosa	
PNTO 5527	CAAT-box	CCAAT	Arabidopsis thaliana	common cis-acting element in promoter and enhancer regions
PNTO 5527	CAAT-box	CAAT	Nicotiana glutinosa	
PNTO 5527	CAAT-box	CAAT	Nicotiana glutinosa	
PNTO 5527	CAAT-box	CAAAT	Pisum sativum	common cis-acting element in promoter and enhancer regions
PNTO 5527	CAAT-box	CAAAT	Pisum sativum	common cis-acting element in promoter and enhancer regions
PNTO 5527	CAAT-box	CAAT	Nicotiana glutinosa	
PNTO 5527	CAAT-box	CAAT	Nicotiana glutinosa	
PNTO 5527	CAAT-box	CAAT	Nicotiana glutinosa	
PNTO 5527	CAAT-box	CAAAT	Pisum sativum	common cis-acting element in promoter and enhancer regions
PNTO 5527	CAAT-box	CAAT	Nicotiana glutinosa	
PNTO 5527	CAAT-box	CAAT	Nicotiana glutinosa	
PNTO 5527	CAAT-box	CAAT	Nicotiana glutinosa	
PNTO 5527	CAAT-box	CAAT	Nicotiana glutinosa	
PNTO 5527	CAAT-box	CAAT	Nicotiana glutinosa	
PNTO 5527	CAAT-box	CAAAT	Pisum sativum	common cis-acting element in promoter and enhancer regions
PNTO 5527	CAAT-box	CAAAT	Pisum sativum	common cis-acting element in promoter and enhancer regions
PNTO 5527	CAAT-box	CAAT	Nicotiana glutinosa	
PNTO 5527	CAAT-box	CAAT	Nicotiana glutinosa	
PNTO 5527	CAAT-box	CAAT	Nicotiana glutinosa	
PNTO 5527	CAAT-box	CCAAT	Arabidopsis thaliana	common cis-acting element in promoter and enhancer regions
PNTO 5527	CAAT-box	CAAAT	Pisum sativum	common cis-acting element in promoter and enhancer regions

PNT0 5527	AAGAA-motif	gGTAAAGAAA	Avena sativa	
PNT0 5527	Unnamed__1	CGTGG	Zea mays	
PNT0 5527	Unnamed__1	GAATTTAATTAA	Glycine max	60K protein binding site
PNT0 5527	MYB recognition site	CCGTTG	Arabidopsis thaliana	
PNT0 5527	TATA-box	TATAAA	Helianthus annuus	core promoter element around -30 of transcription start
PNT0 5527	TATA-box	TATAA	Arabidopsis thaliana	core promoter element around -30 of transcription start
PNT0 5527	TATA-box	TATA	Arabidopsis thaliana	core promoter element around -30 of transcription start
PNT0 5527	TATA-box	TATTTAAA	Arabidopsis thaliana	core promoter element around -30 of transcription start
PNT0 5527	TATA-box	ATTATA	Brassica napus	core promoter element around -30 of transcription start
PNT0 5527	TATA-box	TATAA	Arabidopsis thaliana	core promoter element around -30 of transcription start
PNT0 5527	TATA-box	TATA	Arabidopsis thaliana	core promoter element around -30 of transcription start
PNT0 5527	TATA-box	TATAAAT	Brassica juncea	core promoter element around -30 of transcription start
PNT0 5527	TATA-box	TATAAA	Helianthus annuus	core promoter element around -30 of transcription start
PNT0 5527	TATA-box	TATAA	Arabidopsis thaliana	core promoter element around -30 of transcription start
PNT0 5527	TATA-box	TATA	Arabidopsis thaliana	core promoter element around -30 of transcription start
PNT0 5527	TATA-box	taTATAAAtc	Arabidopsis thaliana	core promoter element around -30 of transcription start
PNT0 5527	TATA-box	TATAAAT	Brassica juncea	core promoter element around -30 of transcription start
PNT0 5527	TATA-box	TATAAA	Helianthus annuus	core promoter element around -30 of transcription start
PNT0 5527	TATA-box	TATATAA	Arabidopsis thaliana	core promoter element around -30 of transcription start
PNT0 5527	TATA-box	TATATA	Arabidopsis thaliana	core promoter element around -30 of transcription start
PNT0 5527	TATA-box	ATATAT	Brassica napus	core promoter element around -30 of transcription start

PNTO5527	TATA-box	TATATA	Arabidopsis thaliana	core promoter element around -30 of transcription start
PNTO5527	TATA-box	ATATAT	Brassica napus	core promoter element around -30 of transcription start
PNTO5527	TATA-box	TATATA	Arabidopsis thaliana	core promoter element around -30 of transcription start
PNTO5527	TATA-box	ATATAT	Brassica napus	core promoter element around -30 of transcription start
PNTO5527	TATA-box	TATATA	Arabidopsis thaliana	core promoter element around -30 of transcription start
PNTO5527	TATA-box	ATATAT	Brassica napus	core promoter element around -30 of transcription start
PNTO5527	TATA-box	TATATA	Arabidopsis thaliana	core promoter element around -30 of transcription start
PNTO5527	TATA-box	ATATAT	Brassica napus	core promoter element around -30 of transcription start
PNTO5527	TATA-box	TATATA	Arabidopsis thaliana	core promoter element around -30 of transcription start
PNTO5527	TATA-box	ATATAT	Brassica napus	core promoter element around -30 of transcription start
PNTO5527	TATA-box	TATA	Arabidopsis thaliana	core promoter element around -30 of transcription start
PNTO5527	TATA-box	ATATAA	Brassica oleracea	core promoter element around -30 of transcription start
PNTO5527	TATA-box	TATA	Arabidopsis thaliana	core promoter element around -30 of transcription start
PNTO5527	TATA-box	TACAAAA	Oryza sativa	core promoter element around -30 of transcription start
PNTO5527	TATA-box	TATAAAA	Pisum sativum	core promoter element around -30 of transcription start
PNTO5527	TATA-box	TATAAA	Helianthus annuus	core promoter element around -30 of transcription start
PNTO5527	TATA-box	TATAA	Arabidopsis thaliana	core promoter element around -30 of transcription start
PNTO5527	TATA-box	TATA	Arabidopsis thaliana	core promoter element around -30 of transcription start
PNTO5527	TATA-box	TATA	Arabidopsis thaliana	core promoter element around -30 of transcription start
PNTO5527	TATA-box	ATATAA	Brassica oleracea	core promoter element around -30 of transcription start
PNTO5527	TATA-box	TATA	Arabidopsis thaliana	core promoter element around -30 of transcription start
PNTO5527	TATA-box	ATATAA	Brassica oleracea	core promoter element around -30 of transcription start

PNTO 5527	TATA-box	TATA	Arabidopsis thaliana	core promoter element around -30 of transcription start
PNTO 5527	TATA-box	TATAAAA	Pisum sativum	core promoter element around -30 of transcription start
PNTO 5527	TATA-box	TATAAA	Helianthus annuus	core promoter element around -30 of transcription start
PNTO 5527	TATA-box	TATAA	Arabidopsis thaliana	core promoter element around -30 of transcription start
PNTO 5527	TATA-box	TATA	Arabidopsis thaliana	core promoter element around -30 of transcription start
PNTO 5527	TATA-box	ccTATAAAaa	Arabidopsis thaliana	core promoter element around -30 of transcription start
PNTO 5527	TATA-box	TATAAAA	Pisum sativum	core promoter element around -30 of transcription start
PNTO 5527	TATA-box	TATAAA	Helianthus annuus	core promoter element around -30 of transcription start
PNTO 5527	TATA-box	TATAA	Arabidopsis thaliana	core promoter element around -30 of transcription start
PNTO 5527	TATA-box	TATA	Arabidopsis thaliana	core promoter element around -30 of transcription start
PNTO 5527	TATA-box	taTATAAAtc	Arabidopsis thaliana	core promoter element around -30 of transcription start
PNTO 5527	TATA-box	ATATAT	Brassica napus	core promoter element around -30 of transcription start
PNTO 5527	TATA-box	TATATA	Arabidopsis thaliana	core promoter element around -30 of transcription start
PNTO 5527	TATA-box	ATATAT	Brassica napus	core promoter element around -30 of transcription start
PNTO 5527	TATA-box	TATATA	Arabidopsis thaliana	core promoter element around -30 of transcription start
PNTO 5527	TATA-box	ATATAT	Brassica napus	core promoter element around -30 of transcription start
PNTO 5527	TATA-box	TATATA	Arabidopsis thaliana	core promoter element around -30 of transcription start
PNTO 5527	TATA-box	ATATAT	Brassica napus	core promoter element around -30 of transcription start
PNTO 5527	TATA-box	TATATA	Arabidopsis thaliana	core promoter element around -30 of transcription start
PNTO 5527	TATA-box	ATATAT	Brassica napus	core promoter element around -30 of transcription start
PNTO 5527	TATA-box	TATATA	Arabidopsis thaliana	core promoter element around -30 of transcription start
PNTO 5527	TATA-box	ATATAT	Brassica napus	core promoter element around -30 of transcription start

PNTO5527	TATA-box	TATATA	Arabidopsis thaliana	core promoter element around -30 of transcription start
PNTO5527	TATA-box	ATATAT	Brassica napus	core promoter element around -30 of transcription start
PNTO5527	TATA-box	TATATA	Arabidopsis thaliana	core promoter element around -30 of transcription start
PNTO5527	TATA-box	ATATAT	Brassica napus	core promoter element around -30 of transcription start
PNTO5527	TATA-box	TATATA	Arabidopsis thaliana	core promoter element around -30 of transcription start
PNTO5527	TATA-box	ATATAT	Brassica napus	core promoter element around -30 of transcription start
PNTO5527	TATA-box	TATATA	Arabidopsis thaliana	core promoter element around -30 of transcription start
PNTO5527	TATA-box	ATATAT	Brassica napus	core promoter element around -30 of transcription start
PNTO5527	TATA-box	TATA	Arabidopsis thaliana	core promoter element around -30 of transcription start
PNTO5527	TATA-box	TATAAAA	Pisum sativum	core promoter element around -30 of transcription start
PNTO5527	TATA-box	TATAAA	Helianthus annuus	core promoter element around -30 of transcription start
PNTO5527	TATA-box	TATAA	Arabidopsis thaliana	core promoter element around -30 of transcription start
PNTO5527	TATA-box	TATA	Arabidopsis thaliana	core promoter element around -30 of transcription start
PNTO5527	TATA-box	TATTTAAA	Arabidopsis thaliana	core promoter element around -30 of transcription start
PNTO5527	TATA-box	TATATTTATATTT	Avena sativa	core promoter element around -30 of transcription start
PNTO5527	TATA-box	TATAAAA	Pisum sativum	core promoter element around -30 of transcription start
PNTO5527	TATA-box	TATAAA	Helianthus annuus	core promoter element around -30 of transcription start
PNTO5527	TATA-box	TATAA	Arabidopsis thaliana	core promoter element around -30 of transcription start
PNTO5527	TATA-box	TATA	Arabidopsis thaliana	core promoter element around -30 of transcription start
PNTO5527	TATA-box	TATTTAAA	Arabidopsis thaliana	core promoter element around -30 of transcription start
PNTO5527	TATA-box	TATTTAAA	Arabidopsis thaliana	core promoter element around -30 of transcription start
PNTO5527	TATA-box	ATATAT	Brassica napus	core promoter element around -30 of transcription start

PNT05527	TATA-box	TATA	Arabidopsis thaliana	core promoter element around -30 of transcription start
PNT05527	TATA-box	TATTTAAA	Arabidopsis thaliana	core promoter element around -30 of transcription start
PNT05527	TATA-box	TACAAAA	Oryza sativa	core promoter element around -30 of transcription start
PNT05527	LTR	CCGAAA	Hordeum vulgare	cis-acting element involved in low-temperature responsiveness
PNT05527	LTR	CCGAAA	Hordeum vulgare	cis-acting element involved in low-temperature responsiveness
PNT05527	LTR	CCGAAA	Hordeum vulgare	cis-acting element involved in low-temperature responsiveness
PNT05527	LTR	CCGAAA	Hordeum vulgare	cis-acting element involved in low-temperature responsiveness
PNT05527	LTR	CCGAAA	Hordeum vulgare	cis-acting element involved in low-temperature responsiveness
PNT05527	LTR	CCGAAA	Hordeum vulgare	cis-acting element involved in low-temperature responsiveness
PNT05527	STRE	AGGGG	Arabidopsis thaliana	
PNT05527	STRE	AGGGG	Arabidopsis thaliana	
PNT05527	STRE	AGGGG	Arabidopsis thaliana	
PNT05527	O2-site	GATGACATGG	Zea mays	cis-acting regulatory element involved in zein metabolism regulation
PNT05527		motif_sequence	organism	short_function
PNT05527		motif_sequence	organism	short_function
PNT05527		motif_sequence	organism	short_function
PNT05527		motif_sequence	organism	short_function
PNT05527	ERE	ATTTTAAA	Nicotiana glutinos	
PNT05527	ERE	ATTTTAAA	Nicotiana glutinos	
PNT05527	ERE	ATTTTAAA	Nicotiana glutinos	
PNT05527	ERE	ATTTTAAA	Nicotiana glutinos	
PNT05527	Box 4	ATTAAT	Petroselinum crispum	part of a conserved DNA module involved in light responsiveness

PNT05527	Box 4	ATTAAT	Petroselinum crispum	part of a conserved DNA module involved in light responsiveness
PNT05527	MYB	CAACCA	Arabidopsis thaliana	
PNT05527	MYB	TAACCA	Arabidopsis thaliana	
PNT05527	MYB	CAACCA	Arabidopsis thaliana	
PNT05527	MYB	CAACCA	Arabidopsis thaliana	
PNT05527	CCAAT-box	CAACGG	Hordeum vulgare	MYBHv1 binding site
PNT05527	MYB-like sequence	TAACCA	Arabidopsis thaliana	
PNT05527	TATA	TATAAAAT	Arabidopsis thaliana	
PNT05527	TATA	TATAAAAT	Arabidopsis thaliana	
PNT05527	TATA	TATAAAAT	Arabidopsis thaliana	
PNT05527	P-box	CCTTTTG	Oryza sativa	gibberellin-responsive element
PNT05527	Myb	TAACTG	Arabidopsis thaliana	
PNT05527	Myb	CAACTG	Arabidopsis thaliana	
PNT05527	Myb	TAACTG	Arabidopsis thaliana	
PNT05527	MBS	CAACTG	Arabidopsis thaliana	MYB binding site involved in drought-inducibility
PNT05527	GT1-motif	GGTTAAT	Avena sativa	light responsive element
PNT05527	GT1-motif	GGTTAA	Arabidopsis thaliana	light responsive element
PNT05527	AT~TATA-box	TATATAAA	Arabidopsis thaliana	
PNT05527	AT~TATA-box	TATATA	Arabidopsis thaliana	
PNT05527	AT~TATA-box	TATATA	Arabidopsis thaliana	
PNT05527	AT~TATA-box	TATATA	Arabidopsis thaliana	
PNT05527	AT~TATA-box	TATATA	Arabidopsis thaliana	

PNT0<br>5527	AT~TATA-box	TATATA	Arabidopsis<br>thaliana	
PNT0<br>5527	AT~TATA-box	TATATA	Arabidopsis<br>thaliana	
PNT0<br>5527	AT~TATA-box	TATATA	Arabidopsis<br>thaliana	
PNT0<br>5527	AT~TATA-box	TATATA	Arabidopsis<br>thaliana	
PNT0<br>5527	AT~TATA-box	TATATA	Arabidopsis<br>thaliana	
PNT0<br>5527	AT~TATA-box	TATATA	Arabidopsis<br>thaliana	
PNT0<br>5527	AT~TATA-box	TATATA	Arabidopsis<br>thaliana	
PNT0<br>5527	AT~TATA-box	TATATA	Arabidopsis<br>thaliana	
PNT0<br>5527	AT~TATA-box	TATATA	Arabidopsis<br>thaliana	
PNT0<br>5527	AT~TATA-box	TATATA	Arabidopsis<br>thaliana	
PNT2<br>1675	TATA-box	ATATAA	Brassica<br>oleracea	core promoter element around -30 of<br>transcription start
PNT2<br>1675	TATA-box	TATA	Arabidopsis<br>thaliana	core promoter element around -30 of<br>transcription start
PNT2<br>1675	TATA-box	TATA	Arabidopsis<br>thaliana	core promoter element around -30 of<br>transcription start
PNT2<br>1675	TATA-box	taTATAAAtc	Arabidopsis<br>thaliana	core promoter element around -30 of<br>transcription start
PNT2<br>1675	TATA-box	TATA	Arabidopsis<br>thaliana	core promoter element around -30 of<br>transcription start
PNT2<br>1675	TATA-box	ATATAA	Brassica<br>oleracea	core promoter element around -30 of<br>transcription start
PNT2<br>1675	TATA-box	TATA	Arabidopsis<br>thaliana	core promoter element around -30 of<br>transcription start
PNT2<br>1675	TATA-box	ATTATA	Brassica<br>napus	core promoter element around -30 of<br>transcription start
PNT2<br>1675	TATA-box	TATAA	Arabidopsis<br>thaliana	core promoter element around -30 of<br>transcription start
PNT2<br>1675	TATA-box	TATA	Arabidopsis<br>thaliana	core promoter element around -30 of<br>transcription start
PNT2<br>1675	TATA-box	ATATAT	Brassica<br>napus	core promoter element around -30 of<br>transcription start

PNT2 1675	TATA-box	TATA	Arabidopsis thaliana	core promoter element around -30 of transcription start
PNT2 1675	TATA-box	ATATAA	Brassica oleracea	core promoter element around -30 of transcription start
PNT2 1675	TATA-box	TATA	Arabidopsis thaliana	core promoter element around -30 of transcription start
PNT2 1675	ACE	CTAACGTATT	Petroselinum crispum	cis-acting element involved in light responsiveness
PNT2 1675	CGTCA-motif	CGTCA	Hordeum vulgare	cis-acting regulatory element involved in the MeJA-responsiveness
PNT2 1675	CGTCA-motif	CGTCA	Hordeum vulgare	cis-acting regulatory element involved in the MeJA-responsiveness
PNT2 1675	CGTCA-motif	CGTCA	Hordeum vulgare	cis-acting regulatory element involved in the MeJA-responsiveness
PNT2 1675	STRE	AGGGG	Arabidopsis thaliana	
PNT2 1675	STRE	AGGGG	Arabidopsis thaliana	
PNT2 1675	as-1	TGACG	Arabidopsis thaliana	
PNT2 1675	as-1	TGACG	Arabidopsis thaliana	
PNT2 1675	as-1	TGACG	Arabidopsis thaliana	
PNT2 1675	ABRE4	CACGTA	Zea mays	
PNT2 1675	Myc	TCTCTTA	Arabidopsis thaliana	
PNT2 1675	MRE	AACCTAA	Petroselinum crispum	MYB binding site involved in light responsiveness
PNT2 1675	W box	TTGACC	Arabidopsis thaliana	
PNT2 1675	W box	TTGACC	Arabidopsis thaliana	
PNT2 1675	CAAT-box	CAAT	Nicotiana glutinosa	
PNT2 1675	CAAT-box	CCAAT	Arabidopsis thaliana	common cis-acting element in promoter and enhancer regions
PNT2 1675	CAAT-box	CAAT	Nicotiana glutinosa	
PNT2 1675	CAAT-box	CAAT	Nicotiana glutinosa	
PNT2 1675	CAAT-box	CAAT	Nicotiana glutinosa	

PNT2 1675	CAAT-box	CAAT	Nicotiana glutinosa	
PNT2 1675	CAAT-box	CAAT	Nicotiana glutinosa	
PNT2 1675	CAAT-box	CAAAT	Pisum sativum	common cis-acting element in promoter and enhancer regions
PNT2 1675	CAAT-box	CCAAT	Arabidopsis thaliana	common cis-acting element in promoter and enhancer regions
PNT2 1675	CAAT-box	CAAT	Nicotiana glutinosa	
PNT2 1675	CAAT-box	CAAT	Nicotiana glutinosa	
PNT2 1675	CAAT-box	CAAT	Nicotiana glutinosa	
PNT2 1675	CAAT-box	CAAT	Nicotiana glutinosa	
PNT2 1675	CAAT-box	CAAT	Nicotiana glutinosa	
PNT2 1675	CAAT-box	CCAAT	Arabidopsis thaliana	common cis-acting element in promoter and enhancer regions
PNT2 1675	CAAT-box	CAAT	Nicotiana glutinosa	
PNT2 1675	CAAT-box	CAAT	Nicotiana glutinosa	
PNT2 1675	CAAT-box	CAAT	Nicotiana glutinosa	
PNT2 1675	CAAT-box	CAAT	Nicotiana glutinosa	
PNT2 1675	CAAT-box	CAAT	Nicotiana glutinosa	
PNT2 1675	CAAT-box	CAAT	Nicotiana glutinosa	
PNT2 1675	CAAT-box	CAAAT	Pisum sativum	common cis-acting element in promoter and enhancer regions
PNT2 1675	CAAT-box	CAAT	Nicotiana glutinosa	
PNT2 1675	CAAT-box	CAAT	Nicotiana glutinosa	
PNT2 1675	AAGAA-motif	GAAAGAA	Avena sativa	
PNT2 1675	TCA	TCATCTTCAT	Pisum sativum	
PNT2 1675	TC-rich repeats	ATTCTCTAAC	Nicotiana tabacum	cis-acting element involved in defense and stress responsiveness

PNT2 1675	TC-rich repeats	ATTCTCTAAC	Nicotiana tabacum	cis-acting element involved in defense and stress responsiveness
PNT2 1675	TC-rich repeats	ATTCTCTAAC	Nicotiana tabacum	cis-acting element involved in defense and stress responsiveness
PNT2 1675	MYC	CATGTG	Arabidopsis thaliana	
PNT2 1675	Unnamed__4	CTCC	Petroselinum hortense	
PNT2 1675	Unnamed__4	CTCC	Petroselinum hortense	
PNT2 1675	Unnamed__4	CTCC	Petroselinum hortense	
PNT2 1675	Unnamed__4	CTCC	Petroselinum hortense	
PNT2 1675	Unnamed__4	CTCC	Petroselinum hortense	
PNT2 1675	Unnamed__4	CTCC	Petroselinum hortense	
PNT2 1675	Unnamed__4	CTCC	Petroselinum hortense	
PNT2 1675	Unnamed__4	CTCC	Petroselinum hortense	
PNT2 1675	Unnamed__4	CTCC	Petroselinum hortense	
PNT2 1675	ABRE	ACGTG	Arabidopsis thaliana	cis-acting element involved in the abscisic acid responsiveness
PNT2 1675	Myb	TAACTG	Arabidopsis thaliana	
PNT2 1675	P-box	CCTTTTG	Oryza sativa	gibberellin-responsive element
PNT2 1675	GT1-motif	GGTTAAT	Avena sativa	light responsive element
PNT2 1675	GT1-motif	GGTTAA	Arabidopsis thaliana	light responsive element
PNT2 1675	GT1-motif	GGTTAA	Arabidopsis thaliana	light responsive element
PNT2 1675	CAT-box	GCCACT	Arabidopsis thaliana	cis-acting regulatory element related to meristem expression
PNT2 1675	GA-motif	ATAGATAA	Arabidopsis thaliana	part of a light responsive element
PNT2 1675	MYB	CAACCA	Arabidopsis thaliana	
PNT2 1675	MYB	TAACCA	Arabidopsis thaliana	

PNT2 1675	MYB	TAACCA	Arabidopsis thaliana	
PNT2 1675		motif_sequence	organism	short_function
PNT2 1675		motif_sequence	organism	short_function
PNT2 1675		motif_sequence	organism	short_function
PNT2 1675		motif_sequence	organism	short_function
PNT2 1675		motif_sequence	organism	short_function
PNT2 1675	ERE	ATTTTAAA	Nicotiana glutinos	
PNT2 1675	Box 4	ATTAAT	Petroselinum crispum	part of a conserved DNA module involved in light responsiveness
PNT2 1675	Box 4	ATTAAT	Petroselinum crispum	part of a conserved DNA module involved in light responsiveness
PNT2 1675	Box 4	ATTAAT	Petroselinum crispum	part of a conserved DNA module involved in light responsiveness
PNT2 1675	Box 4	ATTAAT	Petroselinum crispum	part of a conserved DNA module involved in light responsiveness
PNT2 1675	Box 4	ATTAAT	Petroselinum crispum	part of a conserved DNA module involved in light responsiveness
PNT2 1675	TGACG-motif	TGACG	Hordeum vulgare	cis-acting regulatory element involved in the MeJA-responsiveness
PNT2 1675	TGACG-motif	TGACG	Hordeum vulgare	cis-acting regulatory element involved in the MeJA-responsiveness
PNT2 1675	TGACG-motif	TGACG	Hordeum vulgare	cis-acting regulatory element involved in the MeJA-responsiveness
PNT2 1675	MYB-like sequence	TAACCA	Arabidopsis thaliana	
PNT2 1675	MYB-like sequence	TAACCA	Arabidopsis thaliana	
PNT2 1675	WRE3	CCACCT	Pisum sativum	
PNT2 1675	ABRE3a	TACGTG	Zea mays	
PNT2 1675	G-box	TACGTG	Arabidopsis thaliana	cis-acting regulatory element involved in light responsiveness
PNT3 2402	P-box	CCTTTTG	Oryza sativa	gibberellin-responsive element
PNT3 2402	AT~TATA-box	TATATA	Arabidopsis thaliana	

PNT3 2402	AT~TATA-box	TATATA	Arabidopsis thaliana	
PNT3 2402	AT~TATA-box	TATATA	Arabidopsis thaliana	
PNT3 2402	CAT-box	GCCACT	Arabidopsis thaliana	cis-acting regulatory element related to meristem expression
PNT3 2402	GT1-motif	GGTTAA	Arabidopsis thaliana	light responsive element
PNT3 2402	Box 4	ATTAAT	Petroselinum crispum	part of a conserved DNA module involved in light responsiveness
PNT3 2402	Box 4	ATTAAT	Petroselinum crispum	part of a conserved DNA module involved in light responsiveness
PNT3 2402	Box 4	ATTAAT	Petroselinum crispum	part of a conserved DNA module involved in light responsiveness
PNT3 2402		motif_sequence	organism	short_function
PNT3 2402		motif_sequence	organism	short_function
PNT3 2402		motif_sequence	organism	short_function
PNT3 2402		motif_sequence	organism	short_function
PNT3 2402		motif_sequence	organism	short_function
PNT3 2402		motif_sequence	organism	short_function
PNT3 2402		motif_sequence	organism	short_function
PNT3 2402		motif_sequence	organism	short_function
PNT3 2402		motif_sequence	organism	short_function
PNT3 2402		motif_sequence	organism	short_function
PNT3 2402	ERE	ATTTTAAA	Nicotiana glutinos	
PNT3 2402	ERE	ATTTTAAA	Nicotiana glutinos	
PNT3 2402	ERE	ATTTTAAA	Nicotiana glutinos	
PNT3 2402	ERE	ATTTTAAA	Nicotiana glutinos	
PNT3 2402	ERE	ATTTTAAA	Nicotiana glutinos	

PNT3 2402	ERE	ATTTTAAA	Nicotiana glutinos	
PNT3 2402	ERE	ATTTCATA	Nicotiana glutinos	
PNT3 2402	ERE	ATTTTAAA	Nicotiana glutinos	
PNT3 2402	MYB	CAACCA	Arabidopsis thaliana	
PNT3 2402	MYB	TAACCA	Arabidopsis thaliana	
PNT3 2402	TCCC-motif	TCTCCCT	Spinacia oleracea	part of a light responsive element
PNT3 2402	MYB-like sequence	TAACCA	Arabidopsis thaliana	
PNT3 2402	WRE3	CCACCT	Pisum sativum	
PNT3 2402	TCA-element	CCATCTTTTT	Nicotiana tabacum	cis-acting element involved in salicylic acid responsiveness
PNT3 2402	TCA-element	CCATCTTTTT	Nicotiana tabacum	cis-acting element involved in salicylic acid responsiveness
PNT3 2402	TATA-box	ATATAA	Brassica oleracea	core promoter element around -30 of transcription start
PNT3 2402	TATA-box	TATA	Arabidopsis thaliana	core promoter element around -30 of transcription start
PNT3 2402	TATA-box	TACAAAA	Oryza sativa	core promoter element around -30 of transcription start
PNT3 2402	TATA-box	TACAAAA	Oryza sativa	core promoter element around -30 of transcription start
PNT3 2402	TATA-box	TATACA	Helianthus annuus	core promoter element around -30 of transcription start
PNT3 2402	TATA-box	TATA	Arabidopsis thaliana	core promoter element around -30 of transcription start
PNT3 2402	TATA-box	TATTTAAA	Arabidopsis thaliana	core promoter element around -30 of transcription start
PNT3 2402	TATA-box	ATATAT	Brassica napus	core promoter element around -30 of transcription start
PNT3 2402	TATA-box	TATA	Arabidopsis thaliana	core promoter element around -30 of transcription start
PNT3 2402	TATA-box	TACAAAA	Oryza sativa	core promoter element around -30 of transcription start
PNT3 2402	TATA-box	TATA	Arabidopsis thaliana	core promoter element around -30 of transcription start
PNT3 2402	TATA-box	TACAAAA	Oryza sativa	core promoter element around -30 of transcription start

PNT3 2402	TATA-box	TATAA	Arabidopsis thaliana	core promoter element around -30 of transcription start
PNT3 2402	TATA-box	TATA	Arabidopsis thaliana	core promoter element around -30 of transcription start
PNT3 2402	TATA-box	TATTTAAA	Arabidopsis thaliana	core promoter element around -30 of transcription start
PNT3 2402	TATA-box	ATTATA	Brassica napus	core promoter element around -30 of transcription start
PNT3 2402	TATA-box	TATAA	Arabidopsis thaliana	core promoter element around -30 of transcription start
PNT3 2402	TATA-box	TATA	Arabidopsis thaliana	core promoter element around -30 of transcription start
PNT3 2402	TATA-box	TATAAAT	Brassica juncea	core promoter element around -30 of transcription start
PNT3 2402	TATA-box	TATAAA	Helianthus annuus	core promoter element around -30 of transcription start
PNT3 2402	TATA-box	TATAA	Arabidopsis thaliana	core promoter element around -30 of transcription start
PNT3 2402	TATA-box	TATA	Arabidopsis thaliana	core promoter element around -30 of transcription start
PNT3 2402	TATA-box	TATATAA	Arabidopsis thaliana	core promoter element around -30 of transcription start
PNT3 2402	TATA-box	TATATA	Arabidopsis thaliana	core promoter element around -30 of transcription start
PNT3 2402	TATA-box	ATATAA	Brassica oleracea	core promoter element around -30 of transcription start
PNT3 2402	TATA-box	TATA	Arabidopsis thaliana	core promoter element around -30 of transcription start
PNT3 2402	TATA-box	ATATAT	Brassica napus	core promoter element around -30 of transcription start
PNT3 2402	TATA-box	TATA	Arabidopsis thaliana	core promoter element around -30 of transcription start
PNT3 2402	TATA-box	TATTTAAA	Arabidopsis thaliana	core promoter element around -30 of transcription start
PNT3 2402	TATA-box	TATAAGAA	Zea mays	core promoter element around -30 of transcription start
PNT3 2402	TATA-box	TATAA	Arabidopsis thaliana	core promoter element around -30 of transcription start
PNT3 2402	TATA-box	TATA	Arabidopsis thaliana	core promoter element around -30 of transcription start
PNT3 2402	TATA-box	ATATAA	Brassica oleracea	core promoter element around -30 of transcription start
PNT3 2402	TATA-box	TATA	Arabidopsis thaliana	core promoter element around -30 of transcription start

PNT32402	TATA-box	TATATA	Arabidopsis thaliana	core promoter element around -30 of transcription start
PNT32402	TATA-box	ATATAT	Brassica napus	core promoter element around -30 of transcription start
PNT32402	TATA-box	TATATA	Arabidopsis thaliana	core promoter element around -30 of transcription start
PNT32402	TATA-box	TATA	Arabidopsis thaliana	core promoter element around -30 of transcription start
PNT32402	ARE	AAACCA	Zea mays	cis-acting regulatory element essential for the anaerobic induction
PNT32402	W box	TTGACC	Arabidopsis thaliana	
PNT32402	W box	TTGACC	Arabidopsis thaliana	
PNT32402	TATC-box	TATCCCA	Oryza sativa	cis-acting element involved in gibberellin-responsiveness
PNT32402	Myc	TCTCTTA	Arabidopsis thaliana	
PNT32402	Unnamed__4	CTCC	Petroselinum hortense	
PNT32402	Unnamed__4	CTCC	Petroselinum hortense	
PNT32402	Unnamed__4	CTCC	Petroselinum hortense	
PNT32402	Unnamed__4	CTCC	Petroselinum hortense	
PNT32402	Unnamed__4	CTCC	Petroselinum hortense	
PNT32402	Unnamed__4	CTCC	Petroselinum hortense	
PNT32402	Unnamed__4	CTCC	Petroselinum hortense	
PNT32402	Unnamed__4	CTCC	Petroselinum hortense	
PNT32402	Unnamed__4	CTCC	Petroselinum hortense	
PNT32402	AT1-motif	AATTATTTTTTATT	Solanum tuberosum	part of a light responsive module
PNT32402	LAMP-element	CTTTATCA	Pisum sativum	part of a light responsive element
PNT32402	MYC	CATTTG	Arabidopsis thaliana	
PNT32402	MYC	CAATTG	Arabidopsis thaliana	

PNT3 2402	AAGAA-motif	GAAAGAA	Avena sativa	
PNT3 2402	AAGAA-motif	GAAAGAA	Avena sativa	
PNT3 2402	CAAT-box	CAAT	Nicotiana glutinosa	
PNT3 2402	CAAT-box	CAAT	Nicotiana glutinosa	
PNT3 2402	CAAT-box	CAAAT	Pisum sativum	common cis-acting element in promoter and enhancer regions
PNT3 2402	CAAT-box	CAAT	Nicotiana glutinosa	
PNT3 2402	CAAT-box	CAAT	Nicotiana glutinosa	
PNT3 2402	CAAT-box	CCAAT	Arabidopsis thaliana	common cis-acting element in promoter and enhancer regions
PNT3 2402	CAAT-box	CAAT	Nicotiana glutinosa	
PNT3 2402	CAAT-box	CAAT	Nicotiana glutinosa	
PNT3 2402	CAAT-box	CAAT	Nicotiana glutinosa	
PNT3 2402	CAAT-box	CAAT	Nicotiana glutinosa	
PNT3 2402	CAAT-box	CAAT	Nicotiana glutinosa	
PNT3 2402	CAAT-box	CAAT	Nicotiana glutinosa	
PNT3 2402	CAAT-box	CAAT	Nicotiana glutinosa	
PNT3 2402	CAAT-box	CAAT	Nicotiana glutinosa	
PNT3 2402	CAAT-box	CAAT	Nicotiana glutinosa	
PNT3 2402	CAAT-box	CAAT	Nicotiana glutinosa	
PNT3 2402	CAAT-box	CAAAT	Pisum sativum	common cis-acting element in promoter and enhancer regions
PNT3 2402	CAAT-box	CAAT	Nicotiana glutinosa	
PNT3 2402	CAAT-box	CAAAT	Pisum sativum	common cis-acting element in promoter and enhancer regions
PNT3 2402	CAAT-box	CAAT	Nicotiana glutinosa	

PNT3 2402	CAAT-box	CAAT	Nicotiana glutinosa	
PNT3 2402	CAAT-box	CAAT	Nicotiana glutinosa	
PNT3 2402	CAAT-box	CAAT	Nicotiana glutinosa	
PNT3 2402	CAAT-box	CAAT	Nicotiana glutinosa	
PNT3 2402	CAAT-box	CAAAT	Pisum sativum	common cis-acting element in promoter and enhancer regions
PNT3 2402	CAAT-box	CAAT	Nicotiana glutinosa	
PNT3 2402	CAAT-box	CAAAT	Pisum sativum	common cis-acting element in promoter and enhancer regions
PNT3 2402	CAAT-box	CAAAT	Pisum sativum	common cis-acting element in promoter and enhancer regions
PNT3 2402	CAAT-box	CAAT	Nicotiana glutinosa	
PNT3 2402	CAAT-box	CAAT	Nicotiana glutinosa	
PNT3 2402	CAAT-box	CAAAT	Pisum sativum	common cis-acting element in promoter and enhancer regions
PNT3 2745	AT~TATA-box	TATATAAA	Arabidopsis thaliana	
PNT3 2745	AT~TATA-box	TATATA	Arabidopsis thaliana	
PNT3 2745	AT~TATA-box	TATATA	Arabidopsis thaliana	
PNT3 2745	AT~TATA-box	TATATA	Arabidopsis thaliana	
PNT3 2745	AT~TATA-box	TATATA	Arabidopsis thaliana	
PNT3 2745	GT1-motif	GGTTAAT	Avena sativa	light responsive element
PNT3 2745	GT1-motif	GGTTAA	Arabidopsis thaliana	light responsive element
PNT3 2745	GT1-motif	GGTTAA	Arabidopsis thaliana	light responsive element
PNT3 2745	GT1-motif	GGTTAA	Arabidopsis thaliana	light responsive element
PNT3 2745	TATA	TATAAAAT	Arabidopsis thaliana	
PNT3 2745	TATA	TATAAAAT	Arabidopsis thaliana	

PNT32745	WRE3	CCACCT	Pisum sativum	
PNT32745	G-box	CACGTC	Zea mays	cis-acting regulatory element involved in light responsiveness
PNT32745	G-box	CACGTC	Zea mays	cis-acting regulatory element involved in light responsiveness
PNT32745	G-box	CACGAC	Zea mays	cis-acting regulatory element involved in light responsiveness
PNT32745	MYB	CAACAG	Arabidopsis thaliana	
PNT32745	GCN4_motif	TGAGTCA	Oryza sativa	cis-regulatory element involved in endosperm expression
PNT32745	TGACG-motif	TGACG	Hordeum vulgare	cis-acting regulatory element involved in the MeJA-responsiveness
PNT32745	TGACG-motif	TGACG	Hordeum vulgare	cis-acting regulatory element involved in the MeJA-responsiveness
PNT32745		motif_sequence	organism	short_function
PNT32745		motif_sequence	organism	short_function
PNT32745	ERE	ATTTTAAA	Nicotiana glutinos	
PNT32745	ERE	ATTTTAAA	Nicotiana glutinos	
PNT32745	as-1	TGACG	Arabidopsis thaliana	
PNT32745	as-1	TGACG	Arabidopsis thaliana	
PNT32745	ATCT-motif	AATCTAATCC	Pisum sativum	part of a conserved DNA module involved in light responsiveness
PNT32745	ATCT-motif	AATCTAATCC	Pisum sativum	part of a conserved DNA module involved in light responsiveness
PNT32745	CGTCA-motif	CGTCA	Hordeum vulgare	cis-acting regulatory element involved in the MeJA-responsiveness
PNT32745	CGTCA-motif	CGTCA	Hordeum vulgare	cis-acting regulatory element involved in the MeJA-responsiveness
PNT32745	GARE-motif	TCTGTTG	Brassica oleracea	gibberellin-responsive element
PNT32745	TATA-box	ccTATAAAaa	Arabidopsis thaliana	core promoter element around -30 of transcription start
PNT32745	TATA-box	TATAAAA	Pisum sativum	core promoter element around -30 of transcription start
PNT32745	TATA-box	TATAAA	Helianthus annuus	core promoter element around -30 of transcription start

PNT3 2745	TATA-box	TATAA	Arabidopsis thaliana	core promoter element around -30 of transcription start
PNT3 2745	TATA-box	TATA	Arabidopsis thaliana	core promoter element around -30 of transcription start
PNT3 2745	TATA-box	TATAAAA	Pisum sativum	core promoter element around -30 of transcription start
PNT3 2745	TATA-box	TATAAA	Helianthus annuus	core promoter element around -30 of transcription start
PNT3 2745	TATA-box	TATAA	Arabidopsis thaliana	core promoter element around -30 of transcription start
PNT3 2745	TATA-box	TATA	Arabidopsis thaliana	core promoter element around -30 of transcription start
PNT3 2745	TATA-box	TATA	Arabidopsis thaliana	core promoter element around -30 of transcription start
PNT3 2745	TATA-box	TATAAGAA	Zea mays	core promoter element around -30 of transcription start
PNT3 2745	TATA-box	TATAA	Arabidopsis thaliana	core promoter element around -30 of transcription start
PNT3 2745	TATA-box	TATA	Arabidopsis thaliana	core promoter element around -30 of transcription start
PNT3 2745	TATA-box	TATTTAAA	Arabidopsis thaliana	core promoter element around -30 of transcription start
PNT3 2745	TATA-box	TATAAAA	Pisum sativum	core promoter element around -30 of transcription start
PNT3 2745	TATA-box	TATAAA	Helianthus annuus	core promoter element around -30 of transcription start
PNT3 2745	TATA-box	TATATAA	Arabidopsis thaliana	core promoter element around -30 of transcription start
PNT3 2745	TATA-box	TATATA	Arabidopsis thaliana	core promoter element around -30 of transcription start
PNT3 2745	TATA-box	ATATAT	Brassica napus	core promoter element around -30 of transcription start
PNT3 2745	TATA-box	TATATA	Arabidopsis thaliana	core promoter element around -30 of transcription start
PNT3 2745	TATA-box	ATATAT	Brassica napus	core promoter element around -30 of transcription start
PNT3 2745	TATA-box	TATATA	Arabidopsis thaliana	core promoter element around -30 of transcription start
PNT3 2745	TATA-box	ATATAA	Brassica oleracea	core promoter element around -30 of transcription start
PNT3 2745	TATA-box	TATA	Arabidopsis thaliana	core promoter element around -30 of transcription start
PNT3 2745	TATA-box	ATTATA	Brassica napus	core promoter element around -30 of transcription start

PNT3 2745	TATA-box	TATATAA	Arabidopsis thaliana	core promoter element around -30 of transcription start
PNT3 2745	TATA-box	TATATA	Arabidopsis thaliana	core promoter element around -30 of transcription start
PNT3 2745	TATA-box	TATA	Arabidopsis thaliana	core promoter element around -30 of transcription start
PNT3 2745	TATA-box	TATA	Arabidopsis thaliana	core promoter element around -30 of transcription start
PNT3 2745	TATA-box	ccTATAAAaa	Arabidopsis thaliana	core promoter element around -30 of transcription start
PNT3 2745	TATA-box	TATA	Arabidopsis thaliana	core promoter element around -30 of transcription start
PNT3 2745	TATA-box	TATAAAA	Pisum sativum	core promoter element around -30 of transcription start
PNT3 2745	TATA-box	TATAAA	Helianthus annuus	core promoter element around -30 of transcription start
PNT3 2745	TATA-box	TATAA	Arabidopsis thaliana	core promoter element around -30 of transcription start
PNT3 2745	TATA-box	TATA	Arabidopsis thaliana	core promoter element around -30 of transcription start
PNT3 2745	Unnamed__1	GAATTTAATTAA	Glycine max	60K protein binding site
PNT3 2745	Unnamed__1	CGTGG	Zea mays	
PNT3 2745	Unnamed__1	CGTGG	Zea mays	
PNT3 2745	AAGAA-motif	GAAAGAA	Avena sativa	
PNT3 2745	WUN-motif	AAATTTCTT	Nicotiana glutinosa	
PNT3 2745	CAAT-box	CAAT	Nicotiana glutinosa	
PNT3 2745	CAAT-box	CAAT	Nicotiana glutinosa	
PNT3 2745	CAAT-box	CAAT	Nicotiana glutinosa	
PNT3 2745	CAAT-box	CAAAT	Pisum sativum	common cis-acting element in promoter and enhancer regions
PNT3 2745	CAAT-box	CCAAT	Arabidopsis thaliana	common cis-acting element in promoter and enhancer regions
PNT3 2745	CAAT-box	CCAAT	Arabidopsis thaliana	common cis-acting element in promoter and enhancer regions
PNT3 2745	CAAT-box	CAAT	Nicotiana glutinosa	

PNT3 2745	CAAT-box	CAAT	Nicotiana glutinosa	
PNT3 2745	CAAT-box	CAAT	Nicotiana glutinosa	
PNT3 2745	CAAT-box	CAAT	Nicotiana glutinosa	
PNT3 2745	CAAT-box	CAAAT	Pisum sativum	common cis-acting element in promoter and enhancer regions
PNT3 2745	CAAT-box	CAAT	Nicotiana glutinosa	
PNT3 2745	CAAT-box	CAAT	Nicotiana glutinosa	
PNT3 2745	CAAT-box	CAAAT	Pisum sativum	common cis-acting element in promoter and enhancer regions
PNT3 2745	CAAT-box	AGCTCAATTTCA	Daucus carota	common cis-acting element in promoter and enhancer regions
PNT3 2745	CAAT-box	CAAT	Nicotiana glutinosa	
PNT3 2745	CAAT-box	CAAT	Nicotiana glutinosa	
PNT3 2745	CAAT-box	CAAAT	Pisum sativum	common cis-acting element in promoter and enhancer regions
PNT3 2745	CAAT-box	CAAT	Nicotiana glutinosa	
PNT3 2745	CAAT-box	CAAT	Nicotiana glutinosa	
PNT3 2745	CAAT-box	CAAAT	Pisum sativum	common cis-acting element in promoter and enhancer regions
PNT3 2745	CAAT-box	CCAAT	Arabidopsis thaliana	common cis-acting element in promoter and enhancer regions
PNT3 2745	CAAT-box	CAAT	Nicotiana glutinosa	
PNT3 2745	CAAT-box	CCAAT	Arabidopsis thaliana	common cis-acting element in promoter and enhancer regions
PNT3 2745	CAAT-box	CAAAT	Pisum sativum	common cis-acting element in promoter and enhancer regions
PNT3 2745	CAAT-box	CCAAT	Arabidopsis thaliana	common cis-acting element in promoter and enhancer regions
PNT3 2745	CAAT-box	CAAT	Nicotiana glutinosa	
PNT3 2745	CAAT-box	CAAAT	Pisum sativum	common cis-acting element in promoter and enhancer regions
PNT3 2745	CAAT-box	CAAT	Nicotiana glutinosa	

PNT3 2745	CAAT-box	CAAAT	Pisum sativum	common cis-acting element in promoter and enhancer regions
PNT3 2745	CAAT-box	CAAAT	Pisum sativum	common cis-acting element in promoter and enhancer regions
PNT3 2745	CAAT-box	CAAT	Nicotiana glutinosa	
PNT3 2745	CAAT-box	CAAT	Nicotiana glutinosa	
PNT3 2745	CAAT-box	CAAAT	Pisum sativum	common cis-acting element in promoter and enhancer regions
PNT3 2745	Myb-binding site	CAACAG	Nicotiana tabacum	
PNT3 2745	ABRE	ACGTG	Arabidopsis thaliana	cis-acting element involved in the abscisic acid responsiveness
PNT3 2745	ABRE	ACGTG	Arabidopsis thaliana	cis-acting element involved in the abscisic acid responsiveness
PNT3 2745	TC-rich repeats	ATTCTCTAAC	Nicotiana tabacum	cis-acting element involved in defense and stress responsiveness
PNT3 2745	TGA-box	TGACGTAA	Glycine max	part of an auxin-responsive element
PNT3 2745	MYC	CATTTG	Arabidopsis thaliana	
PNT3 2745	MYC	CAATTG	Arabidopsis thaliana	
PNT3 2745	MYC	CATGTG	Arabidopsis thaliana	
PNT3 2745	Unnamed__4	CTCC	Petroselinum hortense	
PNT3 2745	Unnamed__4	CTCC	Petroselinum hortense	
PNT3 2745	Unnamed__4	CTCC	Petroselinum hortense	
PNT3 2745	Unnamed__4	CTCC	Petroselinum hortense	
PNT3 2745	Unnamed__4	CTCC	Petroselinum hortense	
PNT3 2745	Unnamed__4	CTCC	Petroselinum hortense	
PNT3 2745	Unnamed__4	CTCC	Petroselinum hortense	
PNT3 2745	Unnamed__4	CTCC	Petroselinum hortense	
PNT3 2745	ARE	AAACCA	Zea mays	cis-acting regulatory element essential for the anaerobic induction

PNT3				cis-acting regulatory element essential
2745	ARE	AAACCA	Zea mays	for the anaerobic induction
PNT3				cis-acting regulatory element essential
2745	ARE	AAACCA	Zea mays	for the anaerobic induction
PNT3			Arabidopsis	
2745	W box	TTGACC	thaliana	
PNT3			Arabidopsis	
2745	AE-box	AGAAACAA	thaliana	part of a module for light response
PNT3			Petroselinum	MYB binding site involved in light
2745	MRE	AACCTAA	crispum	responsiveness
PNT3			Petroselinum	
2745	Unnamed__2	AACCTAACCT	hortense	
PNT3			Arabidopsis	
3885	GT1-motif	GGTTAA	thaliana	light responsive element
PNT3			Arabidopsis	
3885	AT~TATA-box	TATATA	thaliana	
PNT3			Arabidopsis	
3885	AT~TATA-box	TATATA	thaliana	
PNT3			Arabidopsis	
3885	AT~TATA-box	TATATA	thaliana	
PNT3				
3885	ABRE2	CCACGTGG	Zea mays	
PNT3				
3885	P-box	CCTTTTG	Oryza sativa	gibberellin-responsive element
PNT3			Brassica	cis-acting regulatory element involved
3885	G-box	ACACGTGGC	napus	in light responsiveness
PNT3			Arabidopsis	cis-acting regulatory element involved
3885	G-box	CACGTG	thaliana	in light responsiveness
PNT3			Arabidopsis	cis-acting regulatory element involved
3885	G-box	GCCACGTGGA	thaliana	in light responsiveness
PNT3			Arabidopsis	cis-acting regulatory element involved
3885	G-box	CACGTG	thaliana	in light responsiveness
PNT3				
3885	Unnamed__6	taTAAATATct	Zea mays	
PNT3			Arabidopsis	
3885	TATA	TATAAAAT	thaliana	
PNT3	MYB-like		Arabidopsis	
3885	sequence	TAACCA	thaliana	
PNT3			Nicotiana	
3885	ERE	ATTTTAAA	glutinos	
PNT3			Nicotiana	
3885	ERE	ATTTCATA	glutinos	
PNT3		motif_sequen		
3885		ce	organism	short_function

PNT33885		motif_sequence	organism	short_function
PNT33885		motif_sequence	organism	short_function
PNT33885		motif_sequence	organism	short_function
PNT33885	Box 4	ATTAAT	Petroselinum crispum	part of a conserved DNA module involved in light responsiveness
PNT33885	Box 4	ATTAAT	Petroselinum crispum	part of a conserved DNA module involved in light responsiveness
PNT33885	Box 4	ATTAAT	Petroselinum crispum	part of a conserved DNA module involved in light responsiveness
PNT33885	MYB	CAACCA	Arabidopsis thaliana	
PNT33885	MYB	TAACCA	Arabidopsis thaliana	
PNT33885	Box II	CCACGTGGC	Petroselinum crispum	part of a light responsive element
PNT33885	STRE	AGGGG	Arabidopsis thaliana	
PNT33885	ATCT-motif	AATCTAATCC	Pisum sativum	part of a conserved DNA module involved in light responsiveness
PNT33885	Unnamed__1	GCCACGTGGC	Petunia sp.	
PNT33885	Unnamed__1	CGTGG	Zea mays	
PNT33885	Unnamed__1	GCCACGTGGC	Petunia sp.	
PNT33885	Unnamed__1	CGTGG	Zea mays	
PNT33885	Unnamed__1	CGTGG	Zea mays	
PNT33885	Unnamed__1	CGTGG	Zea mays	
PNT33885	Unnamed__1	CGTGG	Zea mays	
PNT33885	TATA-box	ATATAT	Brassica napus	core promoter element around -30 of transcription start
PNT33885	TATA-box	TATA	Arabidopsis thaliana	core promoter element around -30 of transcription start
PNT33885	TATA-box	ccTATAAAaa	Arabidopsis thaliana	core promoter element around -30 of transcription start
PNT33885	TATA-box	ATATAA	Brassica oleracea	core promoter element around -30 of transcription start

PNT3 3885	TATA-box	TATA	Arabidopsis thaliana	core promoter element around -30 of transcription start
PNT3 3885	TATA-box	ATATAT	Brassica napus	core promoter element around -30 of transcription start
PNT3 3885	TATA-box	TATATA	Arabidopsis thaliana	core promoter element around -30 of transcription start
PNT3 3885	TATA-box	ATATAT	Brassica napus	core promoter element around -30 of transcription start
PNT3 3885	TATA-box	TATATA	Arabidopsis thaliana	core promoter element around -30 of transcription start
PNT3 3885	TATA-box	ATATAA	Brassica oleracea	core promoter element around -30 of transcription start
PNT3 3885	TATA-box	TATA	Arabidopsis thaliana	core promoter element around -30 of transcription start
PNT3 3885	TATA-box	ccTATAAAaa	Arabidopsis thaliana	core promoter element around -30 of transcription start
PNT3 3885	TATA-box	TATA	Arabidopsis thaliana	core promoter element around -30 of transcription start
PNT3 3885	TATA-box	TATA	Arabidopsis thaliana	core promoter element around -30 of transcription start
PNT3 3885	TATA-box	ATATAA	Brassica oleracea	core promoter element around -30 of transcription start
PNT3 3885	TATA-box	TATA	Arabidopsis thaliana	core promoter element around -30 of transcription start
PNT3 3885	TATA-box	TATA	Arabidopsis thaliana	core promoter element around -30 of transcription start
PNT3 3885	TATA-box	ATTATA	Brassica napus	core promoter element around -30 of transcription start
PNT3 3885	TATA-box	TATAA	Arabidopsis thaliana	core promoter element around -30 of transcription start
PNT3 3885	TATA-box	TATA	Arabidopsis thaliana	core promoter element around -30 of transcription start
PNT3 3885	TATA-box	TATAAAA	Pisum sativum	core promoter element around -30 of transcription start
PNT3 3885	TATA-box	TATAAA	Helianthus annuus	core promoter element around -30 of transcription start
PNT3 3885	TATA-box	TATAA	Arabidopsis thaliana	core promoter element around -30 of transcription start
PNT3 3885	TATA-box	TATA	Arabidopsis thaliana	core promoter element around -30 of transcription start
PNT3 3885	TATA-box	TATAA	Arabidopsis thaliana	core promoter element around -30 of transcription start
PNT3 3885	TATA-box	TATA	Arabidopsis thaliana	core promoter element around -30 of transcription start

PNT3 3885	TATA-box	ATATAT	Brassica napus	core promoter element around -30 of transcription start
PNT3 3885	TATA-box	TATATA	Arabidopsis thaliana	core promoter element around -30 of transcription start
PNT3 3885	TATA-box	ATATAA	Brassica oleracea	core promoter element around -30 of transcription start
PNT3 3885	TATA-box	TATA	Arabidopsis thaliana	core promoter element around -30 of transcription start
PNT3 3885	Unnamed__4	CTCC	Petroselinum hortense	
PNT3 3885	Unnamed__4	CTCC	Petroselinum hortense	
PNT3 3885	AT1-motif	AATTATTTTTTATT	Solanum tuberosum	part of a light responsive module
PNT3 3885	MYC	CATGTG	Arabidopsis thaliana	
PNT3 3885	MYC	CATGTG	Arabidopsis thaliana	
PNT3 3885	TC-rich repeats	ATTCTCTAAC	Nicotiana tabacum	cis-acting element involved in defense and stress responsiveness
PNT3 3885	ABRE	GCAACGTGTC	Hordeum vulgare	cis-acting element involved in the abscisic acid responsiveness
PNT3 3885	ABRE	CACGTG	Arabidopsis thaliana	cis-acting element involved in the abscisic acid responsiveness
PNT3 3885	ABRE	ACGTG	Arabidopsis thaliana	cis-acting element involved in the abscisic acid responsiveness
PNT3 3885	ABRE	CACGTG	Arabidopsis thaliana	cis-acting element involved in the abscisic acid responsiveness
PNT3 3885	ABRE	ACGTG	Arabidopsis thaliana	cis-acting element involved in the abscisic acid responsiveness
PNT3 3885	ABRE	ACGTG	Arabidopsis thaliana	cis-acting element involved in the abscisic acid responsiveness
PNT3 3885	CAAT-box	CAAT	Nicotiana glutinosa	
PNT3 3885	CAAT-box	CAAAT	Pisum sativum	common cis-acting element in promoter and enhancer regions
PNT3 3885	CAAT-box	CAAT	Nicotiana glutinosa	
PNT3 3885	CAAT-box	CAAAT	Pisum sativum	common cis-acting element in promoter and enhancer regions
PNT3 3885	CAAT-box	CAAT	Nicotiana glutinosa	
PNT3 3885	CAAT-box	CAAT	Nicotiana glutinosa	

PNT3 3885	CAAT-box	CAAAT	Pisum sativum	common cis-acting element in promoter and enhancer regions
PNT3 3885	CAAT-box	CAAAT	Pisum sativum	common cis-acting element in promoter and enhancer regions
PNT3 3885	CAAT-box	CAAT	Nicotiana glutinosa	
PNT3 3885	CAAT-box	CAAT	Nicotiana glutinosa	
PNT3 3885	CAAT-box	CAAAT	Pisum sativum	common cis-acting element in promoter and enhancer regions
PNT3 3885	CAAT-box	CAAAT	Pisum sativum	common cis-acting element in promoter and enhancer regions
PNT3 3885	CAAT-box	CAAT	Nicotiana glutinosa	
PNT3 3885	CAAT-box	CAAT	Nicotiana glutinosa	
PNT3 3885	CAAT-box	CAAT	Nicotiana glutinosa	
PNT3 3885	CAAT-box	CAAT	Nicotiana glutinosa	
PNT3 3885	CAAT-box	CAAAT	Pisum sativum	common cis-acting element in promoter and enhancer regions
PNT3 3885	CAAT-box	CAAAT	Pisum sativum	common cis-acting element in promoter and enhancer regions
PNT3 3885	CAAT-box	CAAT	Nicotiana glutinosa	
PNT3 3885	CAAT-box	CAAT	Nicotiana glutinosa	
PNT3 3885	CAAT-box	CCAAT	Arabidopsis thaliana	common cis-acting element in promoter and enhancer regions
PNT3 3885	CAAT-box	CAAT	Nicotiana glutinosa	
PNT3 3885	CAAT-box	CAAT	Nicotiana glutinosa	
PNT3 3885	CAAT-box	CAAAT	Pisum sativum	common cis-acting element in promoter and enhancer regions
PNT3 3885	AAGAA-motif	GAAAGAA	Avena sativa	
PNT3 3885	W box	TTGACC	Arabidopsis thaliana	
PNT3 3885	W box	TTGACC	Arabidopsis thaliana	
PNT3 3885	G-Box	CACGTG	Pisum sativum	cis-acting regulatory element involved in light responsiveness

PNT33885	G-Box	TCCACATGGCA	Triticum aestivum	cis-acting regulatory element involved in light responsiveness
PNT33885	G-Box	CACGTG	Pisum sativum	cis-acting regulatory element involved in light responsiveness
PNT33885	G-Box	CACGTT	Pisum sativum	cis-acting regulatory element involved in light responsiveness
PNT39610	MYB-like sequence	TAACCA	Arabidopsis thaliana	
PNT39610	WRE3	CCACCT	Pisum sativum	
PNT39610	AP-1	TGAGTTAG	Arabidopsis thaliana	
PNT39610	G-box	CACGTC	Zea mays	cis-acting regulatory element involved in light responsiveness
PNT39610	MYB	CAACAG	Arabidopsis thaliana	
PNT39610	MYB	TAACCA	Arabidopsis thaliana	
PNT39610	Box 4	ATTAAT	Petroselinum crispum	part of a conserved DNA module involved in light responsiveness
PNT39610	Box 4	ATTAAT	Petroselinum crispum	part of a conserved DNA module involved in light responsiveness
PNT39610	Box 4	ATTAAT	Petroselinum crispum	part of a conserved DNA module involved in light responsiveness
PNT39610		motif_sequence	organism	short_function
PNT39610		motif_sequence	organism	short_function
PNT39610		motif_sequence	organism	short_function
PNT39610		motif_sequence	organism	short_function
PNT39610		motif_sequence	organism	short_function
PNT39610		motif_sequence	organism	short_function
PNT39610		motif_sequence	organism	short_function
PNT39610		motif_sequence	organism	short_function
PNT39610		motif_sequence	organism	short_function
PNT39610		motif_sequence	organism	short_function

PNT39610		motif_sequence	organism	short_function
PNT39610	GT1-motif	GGTTAA	Arabidopsis thaliana	light responsive element
PNT39610	GT1-motif	GGTTAA	Arabidopsis thaliana	light responsive element
PNT39610	CAAT-box	CAAAT	Pisum sativum	common cis-acting element in promoter and enhancer regions
PNT39610	CAAT-box	CAAT	Nicotiana glutinosa	
PNT39610	CAAT-box	CAAAT	Pisum sativum	common cis-acting element in promoter and enhancer regions
PNT39610	CAAT-box	CCAAT	Arabidopsis thaliana	common cis-acting element in promoter and enhancer regions
PNT39610	CAAT-box	CAAAT	Pisum sativum	common cis-acting element in promoter and enhancer regions
PNT39610	CAAT-box	CAAAT	Pisum sativum	common cis-acting element in promoter and enhancer regions
PNT39610	CAAT-box	CAAAT	Pisum sativum	common cis-acting element in promoter and enhancer regions
PNT39610	CAAT-box	CAAT	Nicotiana glutinosa	
PNT39610	CAAT-box	CCAAT	Arabidopsis thaliana	common cis-acting element in promoter and enhancer regions
PNT39610	CAAT-box	CCAAT	Arabidopsis thaliana	common cis-acting element in promoter and enhancer regions
PNT39610	CAAT-box	CAAT	Nicotiana glutinosa	
PNT39610	CAAT-box	CAAT	Nicotiana glutinosa	
PNT39610	CAAT-box	CAAT	Nicotiana glutinosa	
PNT39610	CAAT-box	CAAAT	Pisum sativum	common cis-acting element in promoter and enhancer regions
PNT39610	CAAT-box	CAAT	Nicotiana glutinosa	
PNT39610	CAAT-box	CAAT	Nicotiana glutinosa	
PNT39610	CAAT-box	CAAT	Nicotiana glutinosa	
PNT39610	CAAT-box	CAAT	Nicotiana glutinosa	
PNT39610	CAAT-box	CAAT	Nicotiana glutinosa	

PNT3 9610	CAAT-box	CAAT	Nicotiana glutinosa	
PNT3 9610	CAAT-box	CAAAT	Pisum sativum	common cis-acting element in promoter and enhancer regions
PNT3 9610	CAAT-box	CAAT	Nicotiana glutinosa	
PNT3 9610	CAAT-box	CAAT	Nicotiana glutinosa	
PNT3 9610	CAAT-box	CCAAT	Arabidopsis thaliana	common cis-acting element in promoter and enhancer regions
PNT3 9610	CAAT-box	CAAT	Nicotiana glutinosa	
PNT3 9610	CAAT-box	CAAAT	Pisum sativum	common cis-acting element in promoter and enhancer regions
PNT3 9610	CAAT-box	CCAAT	Arabidopsis thaliana	common cis-acting element in promoter and enhancer regions
PNT3 9610	CAAT-box	CAAT	Nicotiana glutinosa	
PNT3 9610	CAAT-box	CAAT	Nicotiana glutinosa	
PNT3 9610	CAAT-box	CAAAT	Pisum sativum	common cis-acting element in promoter and enhancer regions
PNT3 9610	CAAT-box	CAAAT	Pisum sativum	common cis-acting element in promoter and enhancer regions
PNT3 9610	CAAT-box	CAAT	Nicotiana glutinosa	
PNT3 9610	CAAT-box	CAAT	Nicotiana glutinosa	
PNT3 9610	CAAT-box	CCAAT	Arabidopsis thaliana	common cis-acting element in promoter and enhancer regions
PNT3 9610	CAAT-box	CAAT	Nicotiana glutinosa	
PNT3 9610	Myb-binding site	CAACAG	Nicotiana tabacum	
PNT3 9610	ABRE	ACGTG	Arabidopsis thaliana	cis-acting element involved in the abscisic acid responsiveness
PNT3 9610	LAMP-element	CTTTATCA	Pisum sativum	part of a light responsive element
PNT3 9610	MYC	CATTTG	Arabidopsis thaliana	
PNT3 9610	TCA	TCATCTTCAT	Pisum sativum	
PNT3 9610	Unnamed__4	CTCC	Petroselinum hortense	

PNT3 9610	Unnamed__4	CTCC	Petroselinum hortense	
PNT3 9610	Unnamed__4	CTCC	Petroselinum hortense	
PNT3 9610	Unnamed__4	CTCC	Petroselinum hortense	
PNT3 9610	Unnamed__4	CTCC	Petroselinum hortense	
PNT3 9610	Unnamed__4	CTCC	Petroselinum hortense	
PNT3 9610	Unnamed__4	CTCC	Petroselinum hortense	
PNT3 9610	ARE	AAACCA	Zea mays	cis-acting regulatory element essential for the anaerobic induction
PNT3 9610	W box	TTGACC	Arabidopsis thaliana	
PNT3 9610	GATA-motif	AAGGATAAGG	Solanum tuberosum	part of a light responsive element
PNT3 9610	AE-box	AGAAACAA	Arabidopsis thaliana	part of a module for light response
PNT3 9610	MRE	AACCTAA	Petroselinum crispum	MYB binding site involved in light responsiveness
PNT3 9610	MRE	AACCTAA	Petroselinum crispum	MYB binding site involved in light responsiveness
PNT3 9610	ATCT-motif	AATCTAATCC	Pisum sativum	part of a conserved DNA module involved in light responsiveness
PNT3 9610	O2-site	GATGATGTGG	Zea mays	cis-acting regulatory element involved in zein metabolism regulation
PNT3 9610	TATA-box	TATA	Arabidopsis thaliana	core promoter element around -30 of transcription start
PNT3 9610	TATA-box	TATA	Arabidopsis thaliana	core promoter element around -30 of transcription start
PNT3 9610	TATA-box	TATA	Arabidopsis thaliana	core promoter element around -30 of transcription start
PNT3 9610	TATA-box	ATTATA	Brassica napus	core promoter element around -30 of transcription start
PNT3 9610	TATA-box	TATAA	Arabidopsis thaliana	core promoter element around -30 of transcription start
PNT3 9610	TATA-box	TATA	Arabidopsis thaliana	core promoter element around -30 of transcription start
PNT3 9610	TATA-box	TATA	Arabidopsis thaliana	core promoter element around -30 of transcription start
PNT3 9610	TATA-box	TATAA	Helianthus annuus	core promoter element around -30 of transcription start

PNT39610	TATA-box	TATAA	Arabidopsis thaliana	core promoter element around -30 of transcription start
PNT39610	TATA-box	TATA	Arabidopsis thaliana	core promoter element around -30 of transcription start
PNT39610	TATA-box	TATAAA	Helianthus annuus	core promoter element around -30 of transcription start
PNT39610	TATA-box	TATAA	Arabidopsis thaliana	core promoter element around -30 of transcription start
PNT39610	TATA-box	TATA	Arabidopsis thaliana	core promoter element around -30 of transcription start
PNT39610	TATA-box	TATA	Arabidopsis thaliana	core promoter element around -30 of transcription start
PNT39610	TATA-box	TATA	Arabidopsis thaliana	core promoter element around -30 of transcription start
PNT39610	TATA-box	TATA	Arabidopsis thaliana	core promoter element around -30 of transcription start
PNT39610	Unnamed__1	CGTGG	Zea mays	
PNT39610	Unnamed__1	CGTGG	Zea mays	
PNT39610	TCA-element	CCATCTTTTT	Nicotiana tabacum	cis-acting element involved in salicylic acid responsiveness
PNT39610	TCA-element	TCAGAAGAGG	Brassica oleracea	cis-acting element involved in salicylic acid responsiveness
PNT42179	TATA	TATAAAAT	Arabidopsis thaliana	
PNT42179	WRE3	CCACCT	Pisum sativum	
PNT42179	WRE3	CCACCT	Pisum sativum	
PNT42179	ABRE3a	TACGTG	Zea mays	
PNT42179	DRE core	GCCGAC	Arabidopsis thaliana	
PNT42179	Unnamed__10	TCCACGTAGA	Zea mays	
PNT42179	G-box	TAACACGTAG	Brassica oleracea	cis-acting regulatory element involved in light responsiveness
PNT42179	G-box	TACGTG	Arabidopsis thaliana	cis-acting regulatory element involved in light responsiveness
PNT42179	Box 4	ATTAAT	Petroselinum crispum	part of a conserved DNA module involved in light responsiveness
PNT42179	Box 4	ATTAAT	Petroselinum crispum	part of a conserved DNA module involved in light responsiveness

PNT42179	Box 4	ATTAAT	Petroselinum crispum	part of a conserved DNA module involved in light responsiveness
PNT42179	Box 4	ATTAAT	Petroselinum crispum	part of a conserved DNA module involved in light responsiveness
PNT42179		motif_sequence	organism	short_function
PNT42179		motif_sequence	organism	short_function
PNT42179		motif_sequence	organism	short_function
PNT42179		motif_sequence	organism	short_function
PNT42179		motif_sequence	organism	short_function
PNT42179		motif_sequence	organism	short_function
PNT42179		motif_sequence	organism	short_function
PNT42179		motif_sequence	organism	short_function
PNT42179		motif_sequence	organism	short_function
PNT42179		motif_sequence	organism	short_function
PNT42179		motif_sequence	organism	short_function
PNT42179		motif_sequence	organism	short_function
PNT42179		motif_sequence	organism	short_function
PNT42179	ERE	ATTTTAAA	Nicotiana glutinos	
PNT42179	I-box	gGATAAGGTG	Zea mays	part of a light responsive element
PNT42179	Unnamed__8	TCCACGTAGA	Zea mays	
PNT42179	AT~TATA-box	TATATA	Arabidopsis thaliana	
PNT42179	AT~TATA-box	TATATAAA	Arabidopsis thaliana	
PNT42179	AT~TATA-box	TATATA	Arabidopsis thaliana	
PNT42179	AT~TATA-box	TATATAAA	Arabidopsis thaliana	

PNT4 2179	AT~TATA-box	TATATA	Arabidopsis thaliana	
PNT4 2179	CAT-box	GCCACT	Arabidopsis thaliana	cis-acting regulatory element related to meristem expression
PNT4 2179	Unnamed__14	TCCACGTAGA	Zea mays	
PNT4 2179	AAGAA-motif	GAAAGAA	Avena sativa	
PNT4 2179	Unnamed__12	TCCACGTAGA	Zea mays	
PNT4 2179	CAAT-box	CAAT	Nicotiana glutinosa	
PNT4 2179	CAAT-box	CAAT	Nicotiana glutinosa	
PNT4 2179	CAAT-box	CAAT	Nicotiana glutinosa	
PNT4 2179	CAAT-box	CAAT	Nicotiana glutinosa	
PNT4 2179	CAAT-box	CAAT	Nicotiana glutinosa	
PNT4 2179	CAAT-box	CAAT	Nicotiana glutinosa	
PNT4 2179	CAAT-box	CCAAT	Arabidopsis thaliana	common cis-acting element in promoter and enhancer regions
PNT4 2179	CAAT-box	CAAT	Nicotiana glutinosa	
PNT4 2179	CAAT-box	CAAAT	Pisum sativum	common cis-acting element in promoter and enhancer regions
PNT4 2179	CAAT-box	CAAT	Nicotiana glutinosa	
PNT4 2179	CAAT-box	CAAT	Nicotiana glutinosa	
PNT4 2179	ABRE	ACGTG	Arabidopsis thaliana	cis-acting element involved in the abscisic acid responsiveness
PNT4 2179	Unnamed__4	CTCC	Petroselinum hortense	
PNT4 2179	Unnamed__4	CTCC	Petroselinum hortense	
PNT4 2179	Unnamed__4	CTCC	Petroselinum hortense	
PNT4 2179	Unnamed__4	CTCC	Petroselinum hortense	
PNT4 2179	AT1-motif	AATTATTTTTTATT	Solanum tuberosum	part of a light responsive module

PNT4 2179	TCA	TCATCTTCAT	Pisum sativum	
PNT4 2179	MYC	CATGTG	Arabidopsis thaliana	
PNT4 2179	ABRE4	CACGTA	Zea mays	
PNT4 2179	ARE	AAACCA	Zea mays	cis-acting regulatory element essential for the anaerobic induction
PNT4 2179	ARE	AAACCA	Zea mays	cis-acting regulatory element essential for the anaerobic induction
PNT4 2179	ARE	AAACCA	Zea mays	cis-acting regulatory element essential for the anaerobic induction
PNT4 2179	AE-box	AGAAACTT	Arabidopsis thaliana	part of a module for light response
PNT4 2179	MRE	AACCTAA	Petroselinum crispum	MYB binding site involved in light responsiveness
PNT4 2179	TCT-motif	TCTTAC	Arabidopsis thaliana	part of a light responsive element
PNT4 2179	TCT-motif	TCTTAC	Arabidopsis thaliana	part of a light responsive element
PNT4 2179	Box II	ACACGTAGA	Arabidopsis thaliana	part of a light responsive element
PNT4 2179	TATA-box	ATTATA	Brassica napus	core promoter element around -30 of transcription start
PNT4 2179	TATA-box	TATAA	Arabidopsis thaliana	core promoter element around -30 of transcription start
PNT4 2179	TATA-box	TATA	Arabidopsis thaliana	core promoter element around -30 of transcription start
PNT4 2179	TATA-box	TATTTAAA	Arabidopsis thaliana	core promoter element around -30 of transcription start
PNT4 2179	TATA-box	ATATAA	Brassica oleracea	core promoter element around -30 of transcription start
PNT4 2179	TATA-box	TATA	Arabidopsis thaliana	core promoter element around -30 of transcription start
PNT4 2179	TATA-box	ATTATA	Brassica napus	core promoter element around -30 of transcription start
PNT4 2179	TATA-box	TATAA	Arabidopsis thaliana	core promoter element around -30 of transcription start
PNT4 2179	TATA-box	TATA	Arabidopsis thaliana	core promoter element around -30 of transcription start
PNT4 2179	TATA-box	taTATAAAtc	Arabidopsis thaliana	core promoter element around -30 of transcription start
PNT4 2179	TATA-box	ATATAT	Brassica napus	core promoter element around -30 of transcription start

PNT4 2179	TATA-box	TATATA	Arabidopsis thaliana	core promoter element around -30 of transcription start
PNT4 2179	TATA-box	ATATAT	Brassica napus	core promoter element around -30 of transcription start
PNT4 2179	TATA-box	TATA	Arabidopsis thaliana	core promoter element around -30 of transcription start
PNT4 2179	TATA-box	TACATAAA	Oryza sativa	core promoter element around -30 of transcription start
PNT4 2179	TATA-box	ATTATA	Brassica napus	core promoter element around -30 of transcription start
PNT4 2179	TATA-box	TATAA	Arabidopsis thaliana	core promoter element around -30 of transcription start
PNT4 2179	TATA-box	TATA	Arabidopsis thaliana	core promoter element around -30 of transcription start
PNT4 2179	TATA-box	ATATAT	Brassica napus	core promoter element around -30 of transcription start
PNT4 2179	TATA-box	TATA	Arabidopsis thaliana	core promoter element around -30 of transcription start
PNT4 2179	TATA-box	TATATTTATATTT	Avena sativa	core promoter element around -30 of transcription start
PNT4 2179	TATA-box	TATA	Arabidopsis thaliana	core promoter element around -30 of transcription start
PNT4 2179	TATA-box	TATA	Arabidopsis thaliana	core promoter element around -30 of transcription start
PNT4 2179	TATA-box	ATTATA	Brassica napus	core promoter element around -30 of transcription start
PNT4 2179	TATA-box	TATAA	Arabidopsis thaliana	core promoter element around -30 of transcription start
PNT4 2179	TATA-box	TATA	Arabidopsis thaliana	core promoter element around -30 of transcription start
PNT4 2179	TATA-box	TATA	Arabidopsis thaliana	core promoter element around -30 of transcription start
PNT4 2179	TATA-box	TAAAGATT	Arabidopsis thaliana	core promoter element around -30 of transcription start
PNT4 2179	TATA-box	TATTTAAA	Arabidopsis thaliana	core promoter element around -30 of transcription start
PNT4 2179	TATA-box	TATAAAA	Pisum sativum	core promoter element around -30 of transcription start
PNT4 2179	TATA-box	TATAAA	Helianthus annuus	core promoter element around -30 of transcription start
PNT4 2179	TATA-box	TATATAA	Arabidopsis thaliana	core promoter element around -30 of transcription start
PNT4 2179	TATA-box	TATATA	Arabidopsis thaliana	core promoter element around -30 of transcription start

PNT4 2179	TATA-box	ATATAA	Brassica oleracea	core promoter element around -30 of transcription start
PNT4 2179	TATA-box	TATA	Arabidopsis thaliana	core promoter element around -30 of transcription start
PNT4 2179	TATA-box	taTATAAAtc	Arabidopsis thaliana	core promoter element around -30 of transcription start
PNT4 2179	TATA-box	TATAAAA	Pisum sativum	core promoter element around -30 of transcription start
PNT4 2179	TATA-box	TATAAA	Helianthus annuus	core promoter element around -30 of transcription start
PNT4 2179	TATA-box	TATATAA	Arabidopsis thaliana	core promoter element around -30 of transcription start
PNT4 2179	TATA-box	TATATA	Arabidopsis thaliana	core promoter element around -30 of transcription start
PNT4 2179	TATA-box	ATATAA	Brassica oleracea	core promoter element around -30 of transcription start
PNT4 2179	TATA-box	TATA	Arabidopsis thaliana	core promoter element around -30 of transcription start
PNT4 2179	TATA-box	ATATAT	Brassica napus	core promoter element around -30 of transcription start
PNT4 2179	TATA-box	TATA	Arabidopsis thaliana	core promoter element around -30 of transcription start
PNT4 2179	TATA-box	TATA	Arabidopsis thaliana	core promoter element around -30 of transcription start
PNT4 2179	TATA-box	TATAAATA	Daucus carota	core promoter element around -30 of transcription start
PNT4 2179	TATA-box	TATAAAT	Brassica juncea	core promoter element around -30 of transcription start
PNT4 2179	TATA-box	TATAAA	Helianthus annuus	core promoter element around -30 of transcription start
PNT4 2179	TATA-box	TATAA	Arabidopsis thaliana	core promoter element around -30 of transcription start
PNT4 2179	TATA-box	TATA	Arabidopsis thaliana	core promoter element around -30 of transcription start
PNT4 2179	TATA-box	TATAAA	Helianthus annuus	core promoter element around -30 of transcription start
PNT4 2179	TATA-box	TATAA	Arabidopsis thaliana	core promoter element around -30 of transcription start
PNT4 2179	TATA-box	TATA	Arabidopsis thaliana	core promoter element around -30 of transcription start
PNT4 6250	AT~TATA-box	TATATA	Arabidopsis thaliana	
PNT4 6250	AT~TATA-box	TATATA	Arabidopsis thaliana	

PNT4 6250	AT~TATA-box	TATATA	Arabidopsis thaliana	
PNT4 6250	AT~TATA-box	TATATA	Arabidopsis thaliana	
PNT4 6250	AT~TATA-box	TATATA	Arabidopsis thaliana	
PNT4 6250	AT~TATA-box	TATATA	Arabidopsis thaliana	
PNT4 6250	AT~TATA-box	TATATA	Arabidopsis thaliana	
PNT4 6250	AT~TATA-box	TATATA	Arabidopsis thaliana	
PNT4 6250	AT~TATA-box	TATATA	Arabidopsis thaliana	
PNT4 6250	Unnamed__1	GAATTTAATTAA	Glycine max	60K protein binding site
PNT4 6250	TCA-element	CCATCTTTTT	Nicotiana tabacum	cis-acting element involved in salicylic acid responsiveness
PNT4 6250	MYB recognition site	CCGTTG	Arabidopsis thaliana	
PNT4 6250	TATA-box	ccTATAAAaa	Arabidopsis thaliana	core promoter element around -30 of transcription start
PNT4 6250	TATA-box	ATTATA	Brassica napus	core promoter element around -30 of transcription start
PNT4 6250	TATA-box	TATAA	Arabidopsis thaliana	core promoter element around -30 of transcription start
PNT4 6250	TATA-box	TATA	Arabidopsis thaliana	core promoter element around -30 of transcription start
PNT4 6250	TATA-box	TATTTAAA	Arabidopsis thaliana	core promoter element around -30 of transcription start
PNT4 6250	TATA-box	TATAAAT	Brassica juncea	core promoter element around -30 of transcription start
PNT4 6250	TATA-box	TATAAA	Helianthus annuus	core promoter element around -30 of transcription start
PNT4 6250	TATA-box	TATAA	Arabidopsis thaliana	core promoter element around -30 of transcription start
PNT4 6250	TATA-box	TATA	Arabidopsis thaliana	core promoter element around -30 of transcription start
PNT4 6250	TATA-box	TATAAAA	Pisum sativum	core promoter element around -30 of transcription start
PNT4 6250	TATA-box	TATAAA	Helianthus annuus	core promoter element around -30 of transcription start

PNT4 6250	TATA-box	TATAA	Arabidopsis thaliana	core promoter element around -30 of transcription start
PNT4 6250	TATA-box	TATA	Arabidopsis thaliana	core promoter element around -30 of transcription start
PNT4 6250	TATA-box	TATAAAA	Pisum sativum	core promoter element around -30 of transcription start
PNT4 6250	TATA-box	TATAAA	Helianthus annuus	core promoter element around -30 of transcription start
PNT4 6250	TATA-box	TATAA	Arabidopsis thaliana	core promoter element around -30 of transcription start
PNT4 6250	TATA-box	TATA	Arabidopsis thaliana	core promoter element around -30 of transcription start
PNT4 6250	TATA-box	ATATAT	Brassica napus	core promoter element around -30 of transcription start
PNT4 6250	TATA-box	TATATA	Arabidopsis thaliana	core promoter element around -30 of transcription start
PNT4 6250	TATA-box	ATATAT	Brassica napus	core promoter element around -30 of transcription start
PNT4 6250	TATA-box	TATATA	Arabidopsis thaliana	core promoter element around -30 of transcription start
PNT4 6250	TATA-box	ATATAT	Brassica napus	core promoter element around -30 of transcription start
PNT4 6250	TATA-box	TATATA	Arabidopsis thaliana	core promoter element around -30 of transcription start
PNT4 6250	TATA-box	ATATAT	Brassica napus	core promoter element around -30 of transcription start
PNT4 6250	TATA-box	TATATA	Arabidopsis thaliana	core promoter element around -30 of transcription start
PNT4 6250	TATA-box	ATATAT	Brassica napus	core promoter element around -30 of transcription start
PNT4 6250	TATA-box	TATATA	Arabidopsis thaliana	core promoter element around -30 of transcription start
PNT4 6250	TATA-box	ATATAT	Brassica napus	core promoter element around -30 of transcription start
PNT4 6250	TATA-box	TATATA	Arabidopsis thaliana	core promoter element around -30 of transcription start
PNT4 6250	TATA-box	ATATAT	Brassica napus	core promoter element around -30 of transcription start
PNT4 6250	TATA-box	TATATA	Arabidopsis thaliana	core promoter element around -30 of transcription start
PNT4 6250	TATA-box	ATATAT	Brassica napus	core promoter element around -30 of transcription start
PNT4 6250	TATA-box	TATATA	Arabidopsis thaliana	core promoter element around -30 of transcription start

PNT46250	TATA-box	ATATAT	Brassica napus	core promoter element around -30 of transcription start
PNT46250	TATA-box	TATATA	Arabidopsis thaliana	core promoter element around -30 of transcription start
PNT46250	TATA-box	ATATAT	Brassica napus	core promoter element around -30 of transcription start
PNT46250	TATA-box	TATA	Arabidopsis thaliana	core promoter element around -30 of transcription start
PNT46250	TATA-box	TATAAAA	Pisum sativum	core promoter element around -30 of transcription start
PNT46250	TATA-box	TATAAA	Helianthus annuus	core promoter element around -30 of transcription start
PNT46250	TATA-box	TATAA	Arabidopsis thaliana	core promoter element around -30 of transcription start
PNT46250	TATA-box	TATA	Arabidopsis thaliana	core promoter element around -30 of transcription start
PNT46250	TATA-box	TATAAA	Helianthus annuus	core promoter element around -30 of transcription start
PNT46250	TATA-box	TATAA	Arabidopsis thaliana	core promoter element around -30 of transcription start
PNT46250	TATA-box	TATA	Arabidopsis thaliana	core promoter element around -30 of transcription start
PNT46250	TATA-box	ATATAT	Brassica napus	core promoter element around -30 of transcription start
PNT46250	TATA-box	TATA	Arabidopsis thaliana	core promoter element around -30 of transcription start
PNT46250	TATA-box	TATTTAAA	Arabidopsis thaliana	core promoter element around -30 of transcription start
PNT46250	TATA-box	ATATAA	Brassica oleracea	core promoter element around -30 of transcription start
PNT46250	TATA-box	TATA	Arabidopsis thaliana	core promoter element around -30 of transcription start
PNT46250	TATA-box	TATAAA	Helianthus annuus	core promoter element around -30 of transcription start
PNT46250	TATA-box	TATAA	Arabidopsis thaliana	core promoter element around -30 of transcription start
PNT46250	TATA-box	TATA	Arabidopsis thaliana	core promoter element around -30 of transcription start
PNT46250	AuxRR-core	GGTCCAT	Nicotiana tabacum	cis-acting regulatory element involved in auxin responsiveness
PNT46250	Unnamed__6	taTAAATATct	Zea mays	
PNT46250	CCAAT-box	CAACGG	Hordeum vulgare	MYBHv1 binding site

PNT4 6250	Unnamed__4	CTCC	Petroselinum hortense	
PNT4 6250	Unnamed__4	CTCC	Petroselinum hortense	
PNT4 6250	Unnamed__4	CTCC	Petroselinum hortense	
PNT4 6250	Unnamed__4	CTCC	Petroselinum hortense	
PNT4 6250	Unnamed__4	CTCC	Petroselinum hortense	
PNT4 6250	MYC	CATTTG	Arabidopsis thaliana	
PNT4 6250	MYC	CATGTG	Arabidopsis thaliana	
PNT4 6250	MYC	CAATTG	Arabidopsis thaliana	
PNT4 6250	AT1-motif	AATTATTTTTTATT	Solanum tuberosum	part of a light responsive module
PNT4 6250	TATA	TATAAAAT	Arabidopsis thaliana	
PNT4 6250	TATA	TATAAAAT	Arabidopsis thaliana	
PNT4 6250	TATA	TATAAAAT	Arabidopsis thaliana	
PNT4 6250	WUN-motif	AAATTACT	Nicotiana glutinosa	
PNT4 6250	CAAT-box	CAAT	Nicotiana glutinosa	
PNT4 6250	CAAT-box	CAAAT	Pisum sativum	common cis-acting element in promoter and enhancer regions
PNT4 6250	CAAT-box	CAAT	Nicotiana glutinosa	
PNT4 6250	CAAT-box	CAAAT	Pisum sativum	common cis-acting element in promoter and enhancer regions
PNT4 6250	CAAT-box	CAAT	Nicotiana glutinosa	
PNT4 6250	CAAT-box	CCAAT	Arabidopsis thaliana	common cis-acting element in promoter and enhancer regions
PNT4 6250	CAAT-box	CAAT	Nicotiana glutinosa	
PNT4 6250	CAAT-box	CAAT	Nicotiana glutinosa	

PNT4 6250	CAAT-box	CAAAT	Pisum sativum	common cis-acting element in promoter and enhancer regions
PNT4 6250	CAAT-box	CAAAT	Pisum sativum	common cis-acting element in promoter and enhancer regions
PNT4 6250	CAAT-box	CAAAT	Pisum sativum	common cis-acting element in promoter and enhancer regions
PNT4 6250	CAAT-box	CAAT	Nicotiana glutinosa	
PNT4 6250	CAAT-box	CAAAT	Pisum sativum	common cis-acting element in promoter and enhancer regions
PNT4 6250	CAAT-box	CAAT	Nicotiana glutinosa	
PNT4 6250	CAAT-box	CAAT	Nicotiana glutinosa	
PNT4 6250	CAAT-box	CAAAT	Pisum sativum	common cis-acting element in promoter and enhancer regions
PNT4 6250	CAAT-box	CAAT	Nicotiana glutinosa	
PNT4 6250	CAAT-box	CAAAT	Pisum sativum	common cis-acting element in promoter and enhancer regions
PNT4 6250	CAAT-box	CAAAT	Pisum sativum	common cis-acting element in promoter and enhancer regions
PNT4 6250	CAAT-box	CAAT	Nicotiana glutinosa	
PNT4 6250	CAAT-box	CAAT	Nicotiana glutinosa	
PNT4 6250	CAAT-box	CAAT	Nicotiana glutinosa	
PNT4 6250	CAAT-box	CAAT	Nicotiana glutinosa	
PNT4 6250	CAAT-box	CAAT	Nicotiana glutinosa	
PNT4 6250	CAAT-box	CAAT	Nicotiana glutinosa	
PNT4 6250	CAAT-box	CAAT	Nicotiana glutinosa	
PNT4 6250	CAAT-box	CAAAT	Pisum sativum	common cis-acting element in promoter and enhancer regions
PNT4 6250	CAAT-box	CAAT	Nicotiana glutinosa	
PNT4 6250	CAAT-box	CAAAT	Pisum sativum	common cis-acting element in promoter and enhancer regions
PNT4 6250	CAAT-box	CAAAT	Pisum sativum	common cis-acting element in promoter and enhancer regions

PNT46250	CAAT-box	CAAAT	Pisum sativum	common cis-acting element in promoter and enhancer regions
PNT46250	CAAT-box	CAAT	Nicotiana glutinosa	
PNT46250	CAAT-box	CAAT	Nicotiana glutinosa	
PNT46250	CAAT-box	CAAT	Nicotiana glutinosa	
PNT46250	Box 4	ATTAAT	Petroselinum crispum	part of a conserved DNA module involved in light responsiveness
PNT46250	Box 4	ATTAAT	Petroselinum crispum	part of a conserved DNA module involved in light responsiveness
PNT46250	Box 4	ATTAAT	Petroselinum crispum	part of a conserved DNA module involved in light responsiveness
PNT46250	W box	TTGACC	Arabidopsis thaliana	
PNT46250	W box	TTGACC	Arabidopsis thaliana	
PNT46250		motif_sequence	organism	short_function
PNT46250		motif_sequence	organism	short_function
PNT46250		motif_sequence	organism	short_function
PNT46250		motif_sequence	organism	short_function
PNT46250		motif_sequence	organism	short_function
PNT46250		motif_sequence	organism	short_function
PNT46250	AE-box	AGAAACTT	Arabidopsis thaliana	part of a module for light response
PNT46250	ERE	ATTTTAAA	Nicotiana glutinos	
PNT46340	TCA-element	TCAGAAGAGG	Brassica oleracea	cis-acting element involved in salicylic acid responsiveness
PNT46340	TCA-element	TCAGAAGAGG	Brassica oleracea	cis-acting element involved in salicylic acid responsiveness
PNT46340	TATA-box	ATATAT	Brassica napus	core promoter element around -30 of transcription start
PNT46340	TATA-box	TATATA	Arabidopsis thaliana	core promoter element around -30 of transcription start
PNT46340	TATA-box	ATATAA	Brassica oleracea	core promoter element around -30 of transcription start

PNT46340	TATA-box	TATA	Arabidopsis thaliana	core promoter element around -30 of transcription start
PNT46340	TATA-box	TATA	Arabidopsis thaliana	core promoter element around -30 of transcription start
PNT46340	TATA-box	TATA	Arabidopsis thaliana	core promoter element around -30 of transcription start
PNT46340	TATA-box	ATATAT	Brassica napus	core promoter element around -30 of transcription start
PNT46340	TATA-box	TATATA	Arabidopsis thaliana	core promoter element around -30 of transcription start
PNT46340	TATA-box	ATATAA	Brassica oleracea	core promoter element around -30 of transcription start
PNT46340	TATA-box	TATA	Arabidopsis thaliana	core promoter element around -30 of transcription start
PNT46340	TATA-box	TATTTAAA	Arabidopsis thaliana	core promoter element around -30 of transcription start
PNT46340	TATA-box	ATTATA	Brassica napus	core promoter element around -30 of transcription start
PNT46340	TATA-box	TATATAA	Arabidopsis thaliana	core promoter element around -30 of transcription start
PNT46340	TATA-box	TATATA	Arabidopsis thaliana	core promoter element around -30 of transcription start
PNT46340	TATA-box	ATATAT	Brassica napus	core promoter element around -30 of transcription start
PNT46340	TATA-box	TATA	Arabidopsis thaliana	core promoter element around -30 of transcription start
PNT46340	TATA-box	ATATAT	Brassica napus	core promoter element around -30 of transcription start
PNT46340	TATA-box	TATA	Arabidopsis thaliana	core promoter element around -30 of transcription start
PNT46340	TATA-box	TATTTAAA	Arabidopsis thaliana	core promoter element around -30 of transcription start
PNT46340	TATA-box	TATA	Arabidopsis thaliana	core promoter element around -30 of transcription start
PNT46340	TATA-box	TATATA	Arabidopsis thaliana	core promoter element around -30 of transcription start
PNT46340	TATA-box	ATATAT	Brassica napus	core promoter element around -30 of transcription start
PNT46340	TATA-box	TATATA	Arabidopsis thaliana	core promoter element around -30 of transcription start
PNT46340	TATA-box	ATATAA	Brassica oleracea	core promoter element around -30 of transcription start
PNT46340	TATA-box	TATA	Arabidopsis thaliana	core promoter element around -30 of transcription start

PNT4 6340	TATA-box	ATATAT	Brassica napus	core promoter element around -30 of transcription start
PNT4 6340	TATA-box	TATA	Arabidopsis thaliana	core promoter element around -30 of transcription start
PNT4 6340	TATA-box	TATAAAT	Brassica juncea	core promoter element around -30 of transcription start
PNT4 6340	TATA-box	TATAAA	Helianthus annuus	core promoter element around -30 of transcription start
PNT4 6340	TATA-box	TATAA	Arabidopsis thaliana	core promoter element around -30 of transcription start
PNT4 6340	TATA-box	TATA	Arabidopsis thaliana	core promoter element around -30 of transcription start
PNT4 6340	TATA-box	TATA	Arabidopsis thaliana	core promoter element around -30 of transcription start
PNT4 6340	TATA-box	ATTATA	Brassica napus	core promoter element around -30 of transcription start
PNT4 6340	TATA-box	TATAA	Arabidopsis thaliana	core promoter element around -30 of transcription start
PNT4 6340	TATA-box	TATA	Arabidopsis thaliana	core promoter element around -30 of transcription start
PNT4 6340	TATA-box	TATAA	Arabidopsis thaliana	core promoter element around -30 of transcription start
PNT4 6340	TATA-box	TATA	Arabidopsis thaliana	core promoter element around -30 of transcription start
PNT4 6340	TATA-box	TATTTAAA	Arabidopsis thaliana	core promoter element around -30 of transcription start
PNT4 6340	LTR	CCGAAA	Hordeum vulgare	cis-acting element involved in low-temperature responsiveness
PNT4 6340	GARE-motif	TCTGTTG	Brassica oleracea	gibberellin-responsive element
PNT4 6340	CGTCA-motif	CGTCA	Hordeum vulgare	cis-acting regulatory element involved in the MeJA-responsiveness
PNT4 6340	CGTCA-motif	CGTCA	Hordeum vulgare	cis-acting regulatory element involved in the MeJA-responsiveness
PNT4 6340	as-1	TGACG	Arabidopsis thaliana	
PNT4 6340	as-1	TGACG	Arabidopsis thaliana	
PNT4 6340	O2-site	GTTGACGTGA	Zea mays	cis-acting regulatory element involved in zein metabolism regulation
PNT4 6340	MRE	AACCTAA	Petroselinum crispum	MYB binding site involved in light responsiveness
PNT4 6340	W box	TTGACC	Arabidopsis thaliana	

PNT4				cis-acting regulatory element essential
6340	ARE	AAACCA	Zea mays	for the anaerobic induction
PNT4				cis-acting regulatory element essential
6340	ARE	AAACCA	Zea mays	for the anaerobic induction
PNT4			Arabidopsis	
6340	Myc	TCTCTTA	thaliana	
PNT4			Arabidopsis	
6340	MYC	CATTTG	thaliana	
PNT4			Petroselinum	
6340	Unnamed__4	CTCC	hortense	
PNT4			Petroselinum	
6340	Unnamed__4	CTCC	hortense	
PNT4		AATTATTTTTTA	Solanum	
6340	AT1-motif	TT	tuberosum	part of a light responsive module
PNT4			Arabidopsis	cis-acting element involved in the
6340	ABRE	ACGTG	thaliana	abscisic acid responsiveness
PNT4	Myb-binding		Nicotiana	
6340	site	CAACAG	tabacum	
PNT4				common cis-acting element in promoter
6340	CAAT-box	CAAAT	Pisum sativum	and enhancer regions
PNT4			Nicotiana	
6340	CAAT-box	CAAT	glutinosa	
PNT4				common cis-acting element in promoter
6340	CAAT-box	CAAAT	Pisum sativum	and enhancer regions
PNT4			Arabidopsis	common cis-acting element in promoter
6340	CAAT-box	CCAAT	thaliana	and enhancer regions
PNT4			Nicotiana	
6340	CAAT-box	CAAT	glutinosa	
PNT4				common cis-acting element in promoter
6340	CAAT-box	CAAAT	Pisum sativum	and enhancer regions
PNT4			Nicotiana	
6340	CAAT-box	CAAT	glutinosa	
PNT4				common cis-acting element in promoter
6340	CAAT-box	CAAAT	Pisum sativum	and enhancer regions
PNT4				common cis-acting element in promoter
6340	CAAT-box	CAAAT	Pisum sativum	and enhancer regions
PNT4			Nicotiana	
6340	CAAT-box	CAAT	glutinosa	
PNT4			Nicotiana	
6340	CAAT-box	CAAT	glutinosa	
PNT4			Nicotiana	
6340	CAAT-box	CAAT	glutinosa	
PNT4				common cis-acting element in promoter
6340	CAAT-box	CAAAT	Pisum sativum	and enhancer regions

PNT46340	CAAT-box	CAAAT	Pisum sativum	common cis-acting element in promoter and enhancer regions
PNT46340	CAAT-box	CAAT	Nicotiana glutinosa	
PNT46340	CAAT-box	CAAAT	Pisum sativum	common cis-acting element in promoter and enhancer regions
PNT46340	CAAT-box	CAAT	Nicotiana glutinosa	
PNT46340	CAAT-box	CAAAT	Pisum sativum	common cis-acting element in promoter and enhancer regions
PNT46340	CAAT-box	CAAT	Nicotiana glutinosa	
PNT46340	CAAT-box	CAAT	Nicotiana glutinosa	
PNT46340	CAAT-box	CAAT	Nicotiana glutinosa	
PNT46340	CAAT-box	CCAAT	Arabidopsis thaliana	common cis-acting element in promoter and enhancer regions
PNT46340	CAAT-box	CAAT	Nicotiana glutinosa	
PNT46340	CAAT-box	CAAT	Nicotiana glutinosa	
PNT46340	CAAT-box	CAAT	Nicotiana glutinosa	
PNT46340	CAAT-box	CAAAT	Pisum sativum	common cis-acting element in promoter and enhancer regions
PNT46340	CAAT-box	CAAT	Nicotiana glutinosa	
PNT46340	CAAT-box	CAAT	Nicotiana glutinosa	
PNT46340	AAGAA-motif	GAAAGAA	Avena sativa	
PNT46340	Myb	TAACTG	Arabidopsis thaliana	
PNT46340	circadian	CAAAGATATC	Lycopersicon esculentum	cis-acting regulatory element involved in circadian control
PNT46340	AT~TATA-box	TATATA	Arabidopsis thaliana	
PNT46340	AT~TATA-box	TATATA	Arabidopsis thaliana	
PNT46340	AT~TATA-box	TATATA	Arabidopsis thaliana	
PNT46340	AT~TATA-box	TATATA	Arabidopsis thaliana	

PNT46340	AT~TATA-box	TATATA	Arabidopsis thaliana	
PNT46340	ERE	ATTTTAAA	Nicotiana glutinos	
PNT46340	ERE	ATTTTAAA	Nicotiana glutinos	
PNT46340		motif_sequence	organism	short_function
PNT46340		motif_sequence	organism	short_function
PNT46340		motif_sequence	organism	short_function
PNT46340		motif_sequence	organism	short_function
PNT46340		motif_sequence	organism	short_function
PNT46340		motif_sequence	organism	short_function
PNT46340		motif_sequence	organism	short_function
PNT46340		motif_sequence	organism	short_function
PNT46340		motif_sequence	organism	short_function
PNT46340		motif_sequence	organism	short_function
PNT46340	TGACG-motif	TGACG	Hordeum vulgare	cis-acting regulatory element involved in the MeJA-responsiveness
PNT46340	TGACG-motif	TGACG	Hordeum vulgare	cis-acting regulatory element involved in the MeJA-responsiveness
PNT46340	Box 4	ATTAAT	Petroselinum crispum	part of a conserved DNA module involved in light responsiveness
PNT46340	Box 4	ATTAAT	Petroselinum crispum	part of a conserved DNA module involved in light responsiveness
PNT46340	MYB	CAACCA	Arabidopsis thaliana	
PNT46340	MYB	TAACCA	Arabidopsis thaliana	
PNT46340	MYB	TAACCA	Arabidopsis thaliana	
PNT46340	MYB	CAACAG	Arabidopsis thaliana	
PNT46340	G-box	CACGTC	Zea mays	cis-acting regulatory element involved in light responsiveness

PNT4 6340	MYB-like sequence	TAACCA	Arabidopsis thaliana	
PNT4 6340	MYB-like sequence	TAACCA	Arabidopsis thaliana	
PNT5 2564	AT~TATA-box	TATATA	Arabidopsis thaliana	
PNT5 2564	circadian	CAAAGATATC	Lycopersicon esculentum	cis-acting regulatory element involved in circadian control
PNT5 2564	circadian	CAAAGATATC	Lycopersicon esculentum	cis-acting regulatory element involved in circadian control
PNT5 2564	Myb	TAACTG	Arabidopsis thaliana	
PNT5 2564	Myb	CAACTG	Arabidopsis thaliana	
PNT5 2564	Myb	CAACTG	Arabidopsis thaliana	
PNT5 2564	Myb	TAACTG	Arabidopsis thaliana	
PNT5 2564	MBS	CAACTG	Arabidopsis thaliana	MYB binding site involved in drought-inducibility
PNT5 2564	MBS	CAACTG	Arabidopsis thaliana	MYB binding site involved in drought-inducibility
PNT5 2564	CTAG-motif	ACTAGCAGAA	Avena sativa	
PNT5 2564	MYB-like sequence	TAACCA	Arabidopsis thaliana	
PNT5 2564	MYB-like sequence	TAACCA	Arabidopsis thaliana	
PNT5 2564	WRE3	CCACCT	Pisum sativum	
PNT5 2564		motif_sequence	organism	short_function
PNT5 2564		motif_sequence	organism	short_function
PNT5 2564		motif_sequence	organism	short_function
PNT5 2564		motif_sequence	organism	short_function
PNT5 2564		motif_sequence	organism	short_function
PNT5 2564		motif_sequence	organism	short_function
PNT5 2564	ERE	ATTTTAAA	Nicotiana glutinos	

PNT5 2564	ERE	ATTTTAAA	Nicotiana glutinos	
PNT5 2564	Box 4	ATTAAT	Petroselinum crispum	part of a conserved DNA module involved in light responsiveness
PNT5 2564	Box 4	ATTAAT	Petroselinum crispum	part of a conserved DNA module involved in light responsiveness
PNT5 2564	TGACG-motif	TGACG	Hordeum vulgare	cis-acting regulatory element involved in the MeJA-responsiveness
PNT5 2564	MYB	TAACCA	Arabidopsis thaliana	
PNT5 2564	MYB	TAACCA	Arabidopsis thaliana	
PNT5 2564	MYB	CAACAG	Arabidopsis thaliana	
PNT5 2564	CGTCA-motif	CGTCA	Hordeum vulgare	cis-acting regulatory element involved in the MeJA-responsiveness
PNT5 2564	STRE	AGGGG	Arabidopsis thaliana	
PNT5 2564	STRE	AGGGG	Arabidopsis thaliana	
PNT5 2564	as-1	TGACG	Arabidopsis thaliana	
PNT5 2564	TCA-element	CCATCTTTTT	Nicotiana tabacum	cis-acting element involved in salicylic acid responsiveness
PNT5 2564	TCA-element	TCAGAAGAGG	Brassica oleracea	cis-acting element involved in salicylic acid responsiveness
PNT5 2564	TATA-box	TATA	Arabidopsis thaliana	core promoter element around -30 of transcription start
PNT5 2564	TATA-box	TATA	Arabidopsis thaliana	core promoter element around -30 of transcription start
PNT5 2564	TATA-box	TACAAAA	Oryza sativa	core promoter element around -30 of transcription start
PNT5 2564	TATA-box	TATAA	Arabidopsis thaliana	core promoter element around -30 of transcription start
PNT5 2564	TATA-box	TATA	Arabidopsis thaliana	core promoter element around -30 of transcription start
PNT5 2564	TATA-box	ATTATA	Brassica napus	core promoter element around -30 of transcription start
PNT5 2564	TATA-box	TATAA	Arabidopsis thaliana	core promoter element around -30 of transcription start
PNT5 2564	TATA-box	TATA	Arabidopsis thaliana	core promoter element around -30 of transcription start
PNT5 2564	TATA-box	ATTATA	Brassica napus	core promoter element around -30 of transcription start

PNT5 2564	TATA-box	TATAA	Arabidopsis thaliana	core promoter element around -30 of transcription start
PNT5 2564	TATA-box	TATA	Arabidopsis thaliana	core promoter element around -30 of transcription start
PNT5 2564	TATA-box	ATTATA	Brassica napus	core promoter element around -30 of transcription start
PNT5 2564	TATA-box	TATAA	Arabidopsis thaliana	core promoter element around -30 of transcription start
PNT5 2564	TATA-box	TATA	Arabidopsis thaliana	core promoter element around -30 of transcription start
PNT5 2564	TATA-box	ATTATA	Brassica napus	core promoter element around -30 of transcription start
PNT5 2564	TATA-box	TATAA	Arabidopsis thaliana	core promoter element around -30 of transcription start
PNT5 2564	TATA-box	TATA	Arabidopsis thaliana	core promoter element around -30 of transcription start
PNT5 2564	TATA-box	ATTATA	Brassica napus	core promoter element around -30 of transcription start
PNT5 2564	TATA-box	TATATAA	Arabidopsis thaliana	core promoter element around -30 of transcription start
PNT5 2564	TATA-box	TATATA	Arabidopsis thaliana	core promoter element around -30 of transcription start
PNT5 2564	TATA-box	ATATAA	Brassica oleracea	core promoter element around -30 of transcription start
PNT5 2564	TATA-box	TATA	Arabidopsis thaliana	core promoter element around -30 of transcription start
PNT5 2564	TATA-box	ATTATA	Brassica napus	core promoter element around -30 of transcription start
PNT5 2564	TATA-box	TATAA	Arabidopsis thaliana	core promoter element around -30 of transcription start
PNT5 2564	TATA-box	TATA	Arabidopsis thaliana	core promoter element around -30 of transcription start
PNT5 2564	TATA-box	TATA	Arabidopsis thaliana	core promoter element around -30 of transcription start
PNT5 2564	Unnamed__4	CTCC	Petroselinum hortense	
PNT5 2564	Unnamed__4	CTCC	Petroselinum hortense	
PNT5 2564	Unnamed__4	CTCC	Petroselinum hortense	
PNT5 2564	Unnamed__4	CTCC	Petroselinum hortense	
PNT5 2564	Unnamed__4	CTCC	Petroselinum hortense	

PNT5 2564	TC-rich repeats	GTTTTCTTAC	Nicotiana tabacum	cis-acting element involved in defense and stress responsiveness
PNT5 2564	MYC	CATTTG	Arabidopsis thaliana	
PNT5 2564	MYC	CAATTG	Arabidopsis thaliana	
PNT5 2564	MYC	CATTTG	Arabidopsis thaliana	
PNT5 2564	MYC	CATTTG	Arabidopsis thaliana	
PNT5 2564	box S	AGCCACC	Arabidopsis thaliana	
PNT5 2564	Myb-binding site	CAACAG	Nicotiana tabacum	
PNT5 2564	CAAT-box	CAAT	Nicotiana glutinosa	
PNT5 2564	CAAT-box	CAAT	Nicotiana glutinosa	
PNT5 2564	CAAT-box	CAAAT	Pisum sativum	common cis-acting element in promoter and enhancer regions
PNT5 2564	CAAT-box	CAAT	Nicotiana glutinosa	
PNT5 2564	CAAT-box	CAAAT	Pisum sativum	common cis-acting element in promoter and enhancer regions
PNT5 2564	CAAT-box	CAAT	Nicotiana glutinosa	
PNT5 2564	CAAT-box	CAAT	Nicotiana glutinosa	
PNT5 2564	CAAT-box	CAAT	Nicotiana glutinosa	
PNT5 2564	CAAT-box	CCAAT	Arabidopsis thaliana	common cis-acting element in promoter and enhancer regions
PNT5 2564	CAAT-box	CAAT	Nicotiana glutinosa	
PNT5 2564	CAAT-box	CCAAT	Arabidopsis thaliana	common cis-acting element in promoter and enhancer regions
PNT5 2564	CAAT-box	CAAT	Nicotiana glutinosa	
PNT5 2564	CAAT-box	CAAT	Nicotiana glutinosa	
PNT5 2564	CAAT-box	CAAT	Nicotiana glutinosa	
PNT5 2564	CAAT-box	CCAAT	Arabidopsis thaliana	common cis-acting element in promoter and enhancer regions

PNT5 2564	CAAT-box	CAAT	Nicotiana glutinosa	
PNT5 2564	CAAT-box	CAAT	Nicotiana glutinosa	
PNT5 2564	CAAT-box	CAAAT	Pisum sativum	common cis-acting element in promoter and enhancer regions
PNT5 2564	CAAT-box	CAAT	Nicotiana glutinosa	
PNT5 2564	CAAT-box	CAAT	Nicotiana glutinosa	
PNT5 2564	CAAT-box	CCCAATTT	Arabidopsis thaliana	common cis-acting element in promoter and enhancer regions
PNT5 2564	CAAT-box	CCAAT	Arabidopsis thaliana	common cis-acting element in promoter and enhancer regions
PNT5 2564	CAAT-box	CAAT	Nicotiana glutinosa	
PNT5 2564	CAAT-box	CAAT	Nicotiana glutinosa	
PNT5 2564	CAAT-box	CCAAT	Arabidopsis thaliana	common cis-acting element in promoter and enhancer regions
PNT5 2564	CAAT-box	CAAT	Nicotiana glutinosa	
PNT5 2564	CAAT-box	CAAT	Nicotiana glutinosa	
PNT5 2564	CAAT-box	CAAT	Nicotiana glutinosa	
PNT5 2564	CAAT-box	CAAAT	Pisum sativum	common cis-acting element in promoter and enhancer regions
PNT5 2564	CAAT-box	CAAAT	Pisum sativum	common cis-acting element in promoter and enhancer regions
PNT5 2564	CAAT-box	CAAT	Nicotiana glutinosa	
PNT5 2564	CAAT-box	CAAT	Nicotiana glutinosa	
PNT5 2564	CAAT-box	CAAAT	Pisum sativum	common cis-acting element in promoter and enhancer regions
PNT5 2564	CAAT-box	CAAAT	Pisum sativum	common cis-acting element in promoter and enhancer regions
PNT5 2564	CAAT-box	CAAT	Nicotiana glutinosa	
PNT5 2564	CAAT-box	CAAT	Nicotiana glutinosa	
PNT5 2564	CAAT-box	CAAAT	Pisum sativum	common cis-acting element in promoter and enhancer regions

PNT5 2564	CAAT-box	CAAAT	Pisum sativum	common cis-acting element in promoter and enhancer regions
PNT5 2564	CAAT-box	CAAT	Nicotiana glutinosa	
PNT5 2564	CAAT-box	CAAT	Nicotiana glutinosa	
PNT5 2564	CAAT-box	CAAT	Nicotiana glutinosa	
PNT5 2564	CAAT-box	CAAAT	Pisum sativum	common cis-acting element in promoter and enhancer regions
PNT5 2564	CAAT-box	CCAAT	Arabidopsis thaliana	common cis-acting element in promoter and enhancer regions
PNT5 2564	CAAT-box	CAAT	Nicotiana glutinosa	
PNT5 2564	CAAT-box	CAAT	Nicotiana glutinosa	
PNT5 2564	CAAT-box	CAAT	Nicotiana glutinosa	
PNT5 2564	CAAT-box	CAAT	Nicotiana glutinosa	
PNT5 2564	CAAT-box	CAAAT	Pisum sativum	common cis-acting element in promoter and enhancer regions
PNT5 2564	AE-box	AGAAACAA	Arabidopsis thaliana	part of a module for light response
PNT5 2564	MRE	AACCTAA	Petroselinum crispum	MYB binding site involved in light responsiveness
PNT5 2564	ARE	AAACCA	Zea mays	cis-acting regulatory element essential for the anaerobic induction
PNT5 2564	Myc	TCTCTTA	Arabidopsis thaliana	
PNT5 2569	TATA-box	TATA	Arabidopsis thaliana	core promoter element around -30 of transcription start
PNT5 2569	TATA-box	TATA	Arabidopsis thaliana	core promoter element around -30 of transcription start
PNT5 2569	TATA-box	TACAAAA	Oryza sativa	core promoter element around -30 of transcription start
PNT5 2569	TATA-box	TATAA	Arabidopsis thaliana	core promoter element around -30 of transcription start
PNT5 2569	TATA-box	TATA	Arabidopsis thaliana	core promoter element around -30 of transcription start
PNT5 2569	TATA-box	ATTATA	Brassica napus	core promoter element around -30 of transcription start
PNT5 2569	TATA-box	TATAA	Arabidopsis thaliana	core promoter element around -30 of transcription start

PNT5 2569	TATA-box	TATA	Arabidopsis thaliana	core promoter element around -30 of transcription start
PNT5 2569	TATA-box	ATTATA	Brassica napus	core promoter element around -30 of transcription start
PNT5 2569	TATA-box	TATAA	Arabidopsis thaliana	core promoter element around -30 of transcription start
PNT5 2569	TATA-box	TATA	Arabidopsis thaliana	core promoter element around -30 of transcription start
PNT5 2569	TATA-box	ATTATA	Brassica napus	core promoter element around -30 of transcription start
PNT5 2569	TATA-box	TATAA	Arabidopsis thaliana	core promoter element around -30 of transcription start
PNT5 2569	TATA-box	TATA	Arabidopsis thaliana	core promoter element around -30 of transcription start
PNT5 2569	TATA-box	ATTATA	Brassica napus	core promoter element around -30 of transcription start
PNT5 2569	TATA-box	TATAA	Arabidopsis thaliana	core promoter element around -30 of transcription start
PNT5 2569	TATA-box	TATA	Arabidopsis thaliana	core promoter element around -30 of transcription start
PNT5 2569	TATA-box	ATTATA	Brassica napus	core promoter element around -30 of transcription start
PNT5 2569	TATA-box	TATATAA	Arabidopsis thaliana	core promoter element around -30 of transcription start
PNT5 2569	TATA-box	TATATA	Arabidopsis thaliana	core promoter element around -30 of transcription start
PNT5 2569	TATA-box	ATATAA	Brassica oleracea	core promoter element around -30 of transcription start
PNT5 2569	TATA-box	TATA	Arabidopsis thaliana	core promoter element around -30 of transcription start
PNT5 2569	TATA-box	ATTATA	Brassica napus	core promoter element around -30 of transcription start
PNT5 2569	TATA-box	TATAA	Arabidopsis thaliana	core promoter element around -30 of transcription start
PNT5 2569	TATA-box	TATA	Arabidopsis thaliana	core promoter element around -30 of transcription start
PNT5 2569	TATA-box	TATA	Arabidopsis thaliana	core promoter element around -30 of transcription start
PNT5 2569	TCA-element	CCATCTTTTT	Nicotiana tabacum	cis-acting element involved in salicylic acid responsiveness
PNT5 2569	TCA-element	TCAGAAGAGG	Brassica oleracea	cis-acting element involved in salicylic acid responsiveness
PNT5 2569	as-1	TGACG	Arabidopsis thaliana	

PNT5 2569	as-1	TGACG	Arabidopsis thaliana	
PNT5 2569	as-1	TGACG	Arabidopsis thaliana	
PNT5 2569	CGTCA-motif	CGTCA	Hordeum vulgare	cis-acting regulatory element involved in the MeJA-responsiveness
PNT5 2569	CGTCA-motif	CGTCA	Hordeum vulgare	cis-acting regulatory element involved in the MeJA-responsiveness
PNT5 2569	CGTCA-motif	CGTCA	Hordeum vulgare	cis-acting regulatory element involved in the MeJA-responsiveness
PNT5 2569	STRE	AGGGG	Arabidopsis thaliana	
PNT5 2569	STRE	AGGGG	Arabidopsis thaliana	
PNT5 2569	Myc	TCTCTTA	Arabidopsis thaliana	
PNT5 2569	ARE	AAACCA	Zea mays	cis-acting regulatory element essential for the anaerobic induction
PNT5 2569	AE-box	AGAAACAA	Arabidopsis thaliana	part of a module for light response
PNT5 2569	MRE	AACCTAA	Petroselinum crispum	MYB binding site involved in light responsiveness
PNT5 2569	Unnamed__2	CCCCGG	Zea mays	
PNT5 2569	Myb-binding site	CAACAG	Nicotiana tabacum	
PNT5 2569	CAAT-box	CAAT	Nicotiana glutinosa	
PNT5 2569	CAAT-box	CAAAT	Pisum sativum	common cis-acting element in promoter and enhancer regions
PNT5 2569	CAAT-box	CAAT	Nicotiana glutinosa	
PNT5 2569	CAAT-box	CAAAT	Pisum sativum	common cis-acting element in promoter and enhancer regions
PNT5 2569	CAAT-box	CAAT	Nicotiana glutinosa	
PNT5 2569	CAAT-box	CAAT	Nicotiana glutinosa	
PNT5 2569	CAAT-box	CAAT	Nicotiana glutinosa	
PNT5 2569	CAAT-box	CCAAT	Arabidopsis thaliana	common cis-acting element in promoter and enhancer regions
PNT5 2569	CAAT-box	CAAT	Nicotiana glutinosa	

PNT5 2569	CAAT-box	CCAAT	Arabidopsis thaliana	common cis-acting element in promoter and enhancer regions
PNT5 2569	CAAT-box	CAAT	Nicotiana glutinosa	
PNT5 2569	CAAT-box	CAAT	Nicotiana glutinosa	
PNT5 2569	CAAT-box	CCAAT	Arabidopsis thaliana	common cis-acting element in promoter and enhancer regions
PNT5 2569	CAAT-box	CAAT	Nicotiana glutinosa	
PNT5 2569	CAAT-box	CAAT	Nicotiana glutinosa	
PNT5 2569	CAAT-box	CAAAT	Pisum sativum	common cis-acting element in promoter and enhancer regions
PNT5 2569	CAAT-box	CAAT	Nicotiana glutinosa	
PNT5 2569	CAAT-box	CAAAT	Pisum sativum	common cis-acting element in promoter and enhancer regions
PNT5 2569	CAAT-box	CAAT	Nicotiana glutinosa	
PNT5 2569	CAAT-box	CCCAATTT	Arabidopsis thaliana	common cis-acting element in promoter and enhancer regions
PNT5 2569	CAAT-box	CCAAT	Arabidopsis thaliana	common cis-acting element in promoter and enhancer regions
PNT5 2569	CAAT-box	CAAT	Nicotiana glutinosa	
PNT5 2569	CAAT-box	CAAT	Nicotiana glutinosa	
PNT5 2569	CAAT-box	CCAAT	Arabidopsis thaliana	common cis-acting element in promoter and enhancer regions
PNT5 2569	CAAT-box	CAAT	Nicotiana glutinosa	
PNT5 2569	CAAT-box	CAAT	Nicotiana glutinosa	
PNT5 2569	CAAT-box	CAAT	Nicotiana glutinosa	
PNT5 2569	CAAT-box	CAAAT	Pisum sativum	common cis-acting element in promoter and enhancer regions
PNT5 2569	CAAT-box	CAAAT	Pisum sativum	common cis-acting element in promoter and enhancer regions
PNT5 2569	CAAT-box	CAAT	Nicotiana glutinosa	
PNT5 2569	CAAT-box	CAAT	Nicotiana glutinosa	

PNT5 2569	CAAT-box	CAAAT	Pisum sativum	common cis-acting element in promoter and enhancer regions
PNT5 2569	CAAT-box	CAAAT	Pisum sativum	common cis-acting element in promoter and enhancer regions
PNT5 2569	CAAT-box	CAAT	Nicotiana glutinosa	
PNT5 2569	CAAT-box	CAAT	Nicotiana glutinosa	
PNT5 2569	CAAT-box	CAAAT	Pisum sativum	common cis-acting element in promoter and enhancer regions
PNT5 2569	CAAT-box	CAAT	Nicotiana glutinosa	
PNT5 2569	CAAT-box	CAAT	Nicotiana glutinosa	
PNT5 2569	CAAT-box	CAAT	Nicotiana glutinosa	
PNT5 2569	CAAT-box	CAAAT	Pisum sativum	common cis-acting element in promoter and enhancer regions
PNT5 2569	CAAT-box	CCAAT	Arabidopsis thaliana	common cis-acting element in promoter and enhancer regions
PNT5 2569	CAAT-box	CAAT	Nicotiana glutinosa	
PNT5 2569	CAAT-box	CAAT	Nicotiana glutinosa	
PNT5 2569	CAAT-box	CAAT	Nicotiana glutinosa	
PNT5 2569	CAAT-box	CAAT	Nicotiana glutinosa	
PNT5 2569	CAAT-box	CAAAT	Pisum sativum	common cis-acting element in promoter and enhancer regions
PNT5 2569	box S	AGCCACC	Arabidopsis thaliana	
PNT5 2569	MYC	CATTTG	Arabidopsis thaliana	
PNT5 2569	MYC	CAATTG	Arabidopsis thaliana	
PNT5 2569	MYC	CATTTG	Arabidopsis thaliana	
PNT5 2569	MYC	CATTTG	Arabidopsis thaliana	
PNT5 2569	Unnamed__4	CTCC	Petroselinum hortense	
PNT5 2569	Unnamed__4	CTCC	Petroselinum hortense	

PNT5 2569	Unnamed__4	CTCC	Petroselinum hortense	
PNT5 2569	Unnamed__4	CTCC	Petroselinum hortense	
PNT5 2569	Unnamed__4	CTCC	Petroselinum hortense	
PNT5 2569	TC-rich repeats	GTTTTCTTAC	Nicotiana tabacum	cis-acting element involved in defense and stress responsiveness
PNT5 2569	Myb	TAACTG	Arabidopsis thaliana	
PNT5 2569	Myb	CAACTG	Arabidopsis thaliana	
PNT5 2569	Myb	CAACTG	Arabidopsis thaliana	
PNT5 2569	Myb	TAACTG	Arabidopsis thaliana	
PNT5 2569	MBS	CAACTG	Arabidopsis thaliana	MYB binding site involved in drought-inducibility
PNT5 2569	MBS	CAACTG	Arabidopsis thaliana	MYB binding site involved in drought-inducibility
PNT5 2569	AT~TATA-box	TATATA	Arabidopsis thaliana	
PNT5 2569	circadian	CAAAGATATC	Lycopersicon esculentum	cis-acting regulatory element involved in circadian control
PNT5 2569	circadian	CAAAGATATC	Lycopersicon esculentum	cis-acting regulatory element involved in circadian control
PNT5 2569	MYB	TAACCA	Arabidopsis thaliana	
PNT5 2569	MYB	CAACAG	Arabidopsis thaliana	
PNT5 2569	Box 4	ATTAAT	Petroselinum crispum	part of a conserved DNA module involved in light responsiveness
PNT5 2569	Box 4	ATTAAT	Petroselinum crispum	part of a conserved DNA module involved in light responsiveness
PNT5 2569	Box 4	ATTAAT	Petroselinum crispum	part of a conserved DNA module involved in light responsiveness
PNT5 2569	Box 4	ATTAAT	Petroselinum crispum	part of a conserved DNA module involved in light responsiveness
PNT5 2569	Box 4	ATTAAT	Petroselinum crispum	part of a conserved DNA module involved in light responsiveness
PNT5 2569	TGACG-motif	TGACG	Hordeum vulgare	cis-acting regulatory element involved in the MeJA-responsiveness
PNT5 2569	TGACG-motif	TGACG	Hordeum vulgare	cis-acting regulatory element involved in the MeJA-responsiveness

PNT52569	TGACG-motif	TGACG	Hordeum vulgare	cis-acting regulatory element involved in the MeJA-responsiveness
PNT52569		motif_sequence	organism	short_function
PNT52569		motif_sequence	organism	short_function
PNT52569		motif_sequence	organism	short_function
PNT52569		motif_sequence	organism	short_function
PNT52569		motif_sequence	organism	short_function
PNT52569		motif_sequence	organism	short_function
PNT52569		motif_sequence	organism	short_function
PNT52569		motif_sequence	organism	short_function
PNT52569	ERE	ATTTTAAA	Nicotiana glutinos	
PNT52569	MYB-like sequence	TAACCA	Arabidopsis thaliana	
PNT52569	WRE3	CCACCT	Pisum sativum	
PNT52586	AE-box	AGAAACAA	Arabidopsis thaliana	part of a module for light response
PNT52586	MRE	AACCTAA	Petroselinum crispum	MYB binding site involved in light responsiveness
PNT52586	Unnamed__2	CCCCGG	Zea mays	
PNT52586	ARE	AAACCA	Zea mays	cis-acting regulatory element essential for the anaerobic induction
PNT52586	Myc	TCTCTTA	Arabidopsis thaliana	
PNT52586	MYC	CATTTG	Arabidopsis thaliana	
PNT52586	MYC	CAATTG	Arabidopsis thaliana	
PNT52586	MYC	CATTTG	Arabidopsis thaliana	
PNT52586	MYC	CATTTG	Arabidopsis thaliana	
PNT52586	TC-rich repeats	GTTTTCTTAC	Nicotiana tabacum	cis-acting element involved in defense and stress responsiveness

PNT5 2586	Unnamed__4	CTCC	Petroselinum hortense	
PNT5 2586	Unnamed__4	CTCC	Petroselinum hortense	
PNT5 2586	Unnamed__4	CTCC	Petroselinum hortense	
PNT5 2586	Unnamed__4	CTCC	Petroselinum hortense	
PNT5 2586	box S	AGCCACC	Arabidopsis thaliana	
PNT5 2586	CAAT-box	CAAT	Nicotiana glutinosa	
PNT5 2586	CAAT-box	CAAT	Nicotiana glutinosa	
PNT5 2586	CAAT-box	CAAT	Nicotiana glutinosa	
PNT5 2586	CAAT-box	CAAAT	Pisum sativum	common cis-acting element in promoter and enhancer regions
PNT5 2586	CAAT-box	CAAT	Nicotiana glutinosa	
PNT5 2586	CAAT-box	CAAAT	Pisum sativum	common cis-acting element in promoter and enhancer regions
PNT5 2586	CAAT-box	CAAT	Nicotiana glutinosa	
PNT5 2586	CAAT-box	CAAT	Nicotiana glutinosa	
PNT5 2586	CAAT-box	CAAT	Nicotiana glutinosa	
PNT5 2586	CAAT-box	CCAAT	Arabidopsis thaliana	common cis-acting element in promoter and enhancer regions
PNT5 2586	CAAT-box	CAAT	Nicotiana glutinosa	
PNT5 2586	CAAT-box	CAAT	Nicotiana glutinosa	
PNT5 2586	CAAT-box	CCAAT	Arabidopsis thaliana	common cis-acting element in promoter and enhancer regions
PNT5 2586	CAAT-box	CAAT	Nicotiana glutinosa	
PNT5 2586	CAAT-box	CAAT	Nicotiana glutinosa	
PNT5 2586	CAAT-box	CAAAT	Pisum sativum	common cis-acting element in promoter and enhancer regions

PNT5 2586	CAAT-box	CAAT	Nicotiana glutinosa	
PNT5 2586	CAAT-box	CAAT	Nicotiana glutinosa	
PNT5 2586	CAAT-box	CAAT	Nicotiana glutinosa	
PNT5 2586	CAAT-box	CAAT	Nicotiana glutinosa	
PNT5 2586	CAAT-box	CCAAT	Arabidopsis thaliana	common cis-acting element in promoter and enhancer regions
PNT5 2586	CAAT-box	CAAT	Nicotiana glutinosa	
PNT5 2586	CAAT-box	CAAT	Nicotiana glutinosa	
PNT5 2586	CAAT-box	CAAT	Nicotiana glutinosa	
PNT5 2586	CAAT-box	CAAAT	Pisum sativum	common cis-acting element in promoter and enhancer regions
PNT5 2586	CAAT-box	CAAAT	Pisum sativum	common cis-acting element in promoter and enhancer regions
PNT5 2586	CAAT-box	CAAT	Nicotiana glutinosa	
PNT5 2586	CAAT-box	CAAT	Nicotiana glutinosa	
PNT5 2586	CAAT-box	CAAAT	Pisum sativum	common cis-acting element in promoter and enhancer regions
PNT5 2586	CAAT-box	CAAAT	Pisum sativum	common cis-acting element in promoter and enhancer regions
PNT5 2586	CAAT-box	CAAT	Nicotiana glutinosa	
PNT5 2586	CAAT-box	CAAT	Nicotiana glutinosa	
PNT5 2586	CAAT-box	CAAAT	Pisum sativum	common cis-acting element in promoter and enhancer regions
PNT5 2586	CAAT-box	CAAAT	Pisum sativum	common cis-acting element in promoter and enhancer regions
PNT5 2586	CAAT-box	CAAT	Nicotiana glutinosa	
PNT5 2586	CAAT-box	CAAT	Nicotiana glutinosa	
PNT5 2586	CAAT-box	CAAT	Nicotiana glutinosa	
PNT5 2586	CAAT-box	CAAAT	Pisum sativum	common cis-acting element in promoter and enhancer regions

PNT5 2586	CAAT-box	CCAAT	Arabidopsis thaliana	common cis-acting element in promoter and enhancer regions
PNT5 2586	CAAT-box	CAAT	Nicotiana glutinosa	
PNT5 2586	CAAT-box	CAAT	Nicotiana glutinosa	
PNT5 2586	CAAT-box	CAAT	Nicotiana glutinosa	
PNT5 2586	CAAT-box	CAAT	Nicotiana glutinosa	
PNT5 2586	CAAT-box	CAAAT	Pisum sativum	common cis-acting element in promoter and enhancer regions
PNT5 2586	Unnamed__1	CGTGG	Zea mays	
PNT5 2586	TCA-element	CCATCTTTTT	Nicotiana tabacum	cis-acting element involved in salicylic acid responsiveness
PNT5 2586	TATA-box	TATAAAA	Pisum sativum	core promoter element around -30 of transcription start
PNT5 2586	TATA-box	TATAAA	Helianthus annuus	core promoter element around -30 of transcription start
PNT5 2586	TATA-box	TATAA	Arabidopsis thaliana	core promoter element around -30 of transcription start
PNT5 2586	TATA-box	TATA	Arabidopsis thaliana	core promoter element around -30 of transcription start
PNT5 2586	TATA-box	TATA	Arabidopsis thaliana	core promoter element around -30 of transcription start
PNT5 2586	TATA-box	TATA	Arabidopsis thaliana	core promoter element around -30 of transcription start
PNT5 2586	TATA-box	TACAAAA	Oryza sativa	core promoter element around -30 of transcription start
PNT5 2586	TATA-box	TATAA	Arabidopsis thaliana	core promoter element around -30 of transcription start
PNT5 2586	TATA-box	TATA	Arabidopsis thaliana	core promoter element around -30 of transcription start
PNT5 2586	TATA-box	ATTATA	Brassica napus	core promoter element around -30 of transcription start
PNT5 2586	TATA-box	TATAA	Arabidopsis thaliana	core promoter element around -30 of transcription start
PNT5 2586	TATA-box	TATA	Arabidopsis thaliana	core promoter element around -30 of transcription start
PNT5 2586	TATA-box	ATTATA	Brassica napus	core promoter element around -30 of transcription start
PNT5 2586	TATA-box	TATAA	Arabidopsis thaliana	core promoter element around -30 of transcription start

PNT5 2586	TATA-box	TATA	Arabidopsis thaliana	core promoter element around -30 of transcription start
PNT5 2586	TATA-box	ATTATA	Brassica napus	core promoter element around -30 of transcription start
PNT5 2586	TATA-box	TATAA	Arabidopsis thaliana	core promoter element around -30 of transcription start
PNT5 2586	TATA-box	TATA	Arabidopsis thaliana	core promoter element around -30 of transcription start
PNT5 2586	TATA-box	ATTATA	Brassica napus	core promoter element around -30 of transcription start
PNT5 2586	TATA-box	TATAA	Arabidopsis thaliana	core promoter element around -30 of transcription start
PNT5 2586	TATA-box	TATA	Arabidopsis thaliana	core promoter element around -30 of transcription start
PNT5 2586	TATA-box	ATTATA	Brassica napus	core promoter element around -30 of transcription start
PNT5 2586	TATA-box	TATATAA	Arabidopsis thaliana	core promoter element around -30 of transcription start
PNT5 2586	TATA-box	TATATA	Arabidopsis thaliana	core promoter element around -30 of transcription start
PNT5 2586	TATA-box	ATATAA	Brassica oleracea	core promoter element around -30 of transcription start
PNT5 2586	TATA-box	TATA	Arabidopsis thaliana	core promoter element around -30 of transcription start
PNT5 2586	TATA-box	ATTATA	Brassica napus	core promoter element around -30 of transcription start
PNT5 2586	TATA-box	TATAA	Arabidopsis thaliana	core promoter element around -30 of transcription start
PNT5 2586	TATA-box	TATA	Arabidopsis thaliana	core promoter element around -30 of transcription start
PNT5 2586	TATA-box	TATA	Arabidopsis thaliana	core promoter element around -30 of transcription start
PNT5 2586	STRE	AGGGG	Arabidopsis thaliana	
PNT5 2586		motif_sequence	organism	short_function
PNT5 2586		motif_sequence	organism	short_function
PNT5 2586		motif_sequence	organism	short_function
PNT5 2586		motif_sequence	organism	short_function
PNT5 2586		motif_sequence	organism	short_function

PNT52586		motif_sequence	organism	short_function
PNT52586		motif_sequence	organism	short_function
PNT52586		motif_sequence	organism	short_function
PNT52586	ERE	ATTTTAAA	Nicotiana glutinos	
PNT52586	Box 4	ATTAAT	Petroselinum crispum	part of a conserved DNA module involved in light responsiveness
PNT52586	Box 4	ATTAAT	Petroselinum crispum	part of a conserved DNA module involved in light responsiveness
PNT52586	DRE1	ACCGAGA	Zea mays	
PNT52586	MYB	TAACCA	Arabidopsis thaliana	
PNT52586	CTAG-motif	ACTAGCAGAA	Avena sativa	
PNT52586	MYB-like sequence	TAACCA	Arabidopsis thaliana	
PNT52586	WRE3	CCACCT	Pisum sativum	
PNT52586	Myb	TAACTG	Arabidopsis thaliana	
PNT52586	Myb	CAACTG	Arabidopsis thaliana	
PNT52586	Myb	CAACTG	Arabidopsis thaliana	
PNT52586	Myb	TAACTG	Arabidopsis thaliana	
PNT52586	MBS	CAACTG	Arabidopsis thaliana	MYB binding site involved in drought-inducibility
PNT52586	MBS	CAACTG	Arabidopsis thaliana	MYB binding site involved in drought-inducibility
PNT52586	Sp1	GGGCGG	Oryza sativa	light responsive element
PNT52586	AT~TATA-box	TATATA	Arabidopsis thaliana	
PNT52586	circadian	CAAAGATATC	Lycopersicon esculentum	cis-acting regulatory element involved in circadian control
PNT52586	circadian	CAAAGATATC	Lycopersicon esculentum	cis-acting regulatory element involved in circadian control
PNT52695	GARE-motif	TCTGTTG	Brassica oleracea	gibberellin-responsive element

PNT5 2695	O2-site	GATGATGTGG	Zea mays	cis-acting regulatory element involved in zein metabolism regulation
PNT5 2695	Unnamed__1	CGTGG	Zea mays	
PNT5 2695	Unnamed__1	CGTGG	Zea mays	
PNT5 2695	Unnamed__1	CGTGG	Zea mays	
PNT5 2695	Unnamed__1	CGTGG	Zea mays	
PNT5 2695	TATA-box	TATA	Arabidopsis thaliana	core promoter element around -30 of transcription start
PNT5 2695	TATA-box	TATAAAA	Pisum sativum	core promoter element around -30 of transcription start
PNT5 2695	TATA-box	TATAAA	Helianthus annuus	core promoter element around -30 of transcription start
PNT5 2695	TATA-box	TATATAA	Arabidopsis thaliana	core promoter element around -30 of transcription start
PNT5 2695	TATA-box	TATATA	Arabidopsis thaliana	core promoter element around -30 of transcription start
PNT5 2695	TATA-box	ATATAA	Brassica oleracea	core promoter element around -30 of transcription start
PNT5 2695	TATA-box	TATA	Arabidopsis thaliana	core promoter element around -30 of transcription start
PNT5 2695	TATA-box	TATA	Arabidopsis thaliana	core promoter element around -30 of transcription start
PNT5 2695	TATA-box	TATTTAAA	Arabidopsis thaliana	core promoter element around -30 of transcription start
PNT5 2695	TATA-box	ATATAT	Brassica napus	core promoter element around -30 of transcription start
PNT5 2695	TATA-box	TATATA	Arabidopsis thaliana	core promoter element around -30 of transcription start
PNT5 2695	TATA-box	ATATAT	Brassica napus	core promoter element around -30 of transcription start
PNT5 2695	TATA-box	TATA	Arabidopsis thaliana	core promoter element around -30 of transcription start
PNT5 2695	TATA-box	TATAAAA	Pisum sativum	core promoter element around -30 of transcription start
PNT5 2695	TATA-box	TATAAA	Helianthus annuus	core promoter element around -30 of transcription start
PNT5 2695	TATA-box	TATAA	Arabidopsis thaliana	core promoter element around -30 of transcription start
PNT5 2695	TATA-box	TATA	Arabidopsis thaliana	core promoter element around -30 of transcription start

PNT5 2695	TATA-box	ATATAT	Brassica napus	core promoter element around -30 of transcription start
PNT5 2695	TATA-box	TATATA	Arabidopsis thaliana	core promoter element around -30 of transcription start
PNT5 2695	TATA-box	ATATAA	Brassica oleracea	core promoter element around -30 of transcription start
PNT5 2695	TATA-box	TATA	Arabidopsis thaliana	core promoter element around -30 of transcription start
PNT5 2695	TATA-box	TATAAAA	Pisum sativum	core promoter element around -30 of transcription start
PNT5 2695	TATA-box	TATAAA	Helianthus annuus	core promoter element around -30 of transcription start
PNT5 2695	TATA-box	TATAA	Arabidopsis thaliana	core promoter element around -30 of transcription start
PNT5 2695	TATA-box	TATA	Arabidopsis thaliana	core promoter element around -30 of transcription start
PNT5 2695	TATA-box	ATATAT	Brassica napus	core promoter element around -30 of transcription start
PNT5 2695	TATA-box	TATA	Arabidopsis thaliana	core promoter element around -30 of transcription start
PNT5 2695	TATA-box	ATTATA	Brassica napus	core promoter element around -30 of transcription start
PNT5 2695	TATA-box	TATAA	Arabidopsis thaliana	core promoter element around -30 of transcription start
PNT5 2695	TATA-box	TATA	Arabidopsis thaliana	core promoter element around -30 of transcription start
PNT5 2695	TATA-box	TATAAAA	Pisum sativum	core promoter element around -30 of transcription start
PNT5 2695	TATA-box	TATAAA	Helianthus annuus	core promoter element around -30 of transcription start
PNT5 2695	TATA-box	TATAA	Arabidopsis thaliana	core promoter element around -30 of transcription start
PNT5 2695	TATA-box	TATA	Arabidopsis thaliana	core promoter element around -30 of transcription start
PNT5 2695	TATA-box	ATTATA	Brassica napus	core promoter element around -30 of transcription start
PNT5 2695	TATA-box	TATAA	Arabidopsis thaliana	core promoter element around -30 of transcription start
PNT5 2695	TATA-box	TATA	Arabidopsis thaliana	core promoter element around -30 of transcription start
PNT5 2695	TATA-box	taTATAAAtc	Arabidopsis thaliana	core promoter element around -30 of transcription start
PNT5 2695	TATA-box	ccTATAAAaa	Arabidopsis thaliana	core promoter element around -30 of transcription start

PNT5 2695	TATA-box	TATAAAA	Pisum sativum	core promoter element around -30 of transcription start
PNT5 2695	TATA-box	TATAAA	Helianthus annuus	core promoter element around -30 of transcription start
PNT5 2695	TATA-box	TATAA	Arabidopsis thaliana	core promoter element around -30 of transcription start
PNT5 2695	TATA-box	TATA	Arabidopsis thaliana	core promoter element around -30 of transcription start
PNT5 2695	TATA-box	TATAA	Arabidopsis thaliana	core promoter element around -30 of transcription start
PNT5 2695	TATA-box	TATA	Arabidopsis thaliana	core promoter element around -30 of transcription start
PNT5 2695	TATA-box	TACAAAA	Oryza sativa	core promoter element around -30 of transcription start
PNT5 2695	TATA-box	ATATAA	Brassica oleracea	core promoter element around -30 of transcription start
PNT5 2695	TATA-box	TATA	Arabidopsis thaliana	core promoter element around -30 of transcription start
PNT5 2695	TATA-box	TATA	Arabidopsis thaliana	core promoter element around -30 of transcription start
PNT5 2695	TATA-box	ATATAA	Brassica oleracea	core promoter element around -30 of transcription start
PNT5 2695	TATA-box	TATA	Arabidopsis thaliana	core promoter element around -30 of transcription start
PNT5 2695	TATA-box	TATAAA	Helianthus annuus	core promoter element around -30 of transcription start
PNT5 2695	TATA-box	TATAA	Arabidopsis thaliana	core promoter element around -30 of transcription start
PNT5 2695	TATA-box	TATA	Arabidopsis thaliana	core promoter element around -30 of transcription start
PNT5 2695	TATA-box	TATACA	Helianthus annuus	core promoter element around -30 of transcription start
PNT5 2695	TATA-box	TATA	Arabidopsis thaliana	core promoter element around -30 of transcription start
PNT5 2695	TATA-box	TATAAA	Helianthus annuus	core promoter element around -30 of transcription start
PNT5 2695	TATA-box	TATAA	Arabidopsis thaliana	core promoter element around -30 of transcription start
PNT5 2695	TATA-box	TATA	Arabidopsis thaliana	core promoter element around -30 of transcription start
PNT5 2695	MYC	CATTTG	Arabidopsis thaliana	
PNT5 2695	MYC	CATTTG	Arabidopsis thaliana	

PNT5			Petroselinum	
2695	Unnamed__4	CTCC	hortense	
PNT5			Petroselinum	
2695	Unnamed__4	CTCC	hortense	
PNT5			Petroselinum	
2695	Unnamed__4	CTCC	hortense	
PNT5			Petroselinum	
2695	Unnamed__4	CTCC	hortense	
PNT5			Petroselinum	
2695	Unnamed__4	CTCC	hortense	
PNT5			Petroselinum	
2695	Unnamed__4	CTCC	hortense	
PNT5			Petroselinum	
2695	Unnamed__4	CTCC	hortense	
PNT5				
2695	TCA	TCATCTTCAT	Pisum sativum	
PNT5			Arabidopsis	cis-acting element involved in the
2695	ABRE	ACGTG	thaliana	abscisic acid responsiveness
PNT5			Arabidopsis	cis-acting element involved in the
2695	ABRE	ACGTG	thaliana	abscisic acid responsiveness
PNT5			Nicotiana	
2695	WUN-motif	TTATTACAT	glutinosa	
PNT5			Nicotiana	
2695	WUN-motif	AAATTACT	glutinosa	
PNT5			Nicotiana	
2695	CAAT-box	CAAT	glutinosa	
PNT5				common cis-acting element in promoter
2695	CAAT-box	CAAAT	Pisum sativum	and enhancer regions
PNT5			Nicotiana	
2695	CAAT-box	CAAT	glutinosa	
PNT5			Nicotiana	
2695	CAAT-box	CAAT	glutinosa	
PNT5			Nicotiana	
2695	CAAT-box	CAAT	glutinosa	
PNT5				common cis-acting element in promoter
2695	CAAT-box	CAAAT	Pisum sativum	and enhancer regions
PNT5			Nicotiana	
2695	CAAT-box	CAAT	glutinosa	
PNT5			Nicotiana	
2695	CAAT-box	CAAT	glutinosa	
PNT5				common cis-acting element in promoter
2695	CAAT-box	CAAAT	Pisum sativum	and enhancer regions
PNT5			Nicotiana	
2695	CAAT-box	CAAT	glutinosa	

PNT5 2695	CAAT-box	CAAT	Nicotiana glutinosa	
PNT5 2695	CAAT-box	CAAT	Nicotiana glutinosa	
PNT5 2695	CAAT-box	CCAAT	Arabidopsis thaliana	common cis-acting element in promoter and enhancer regions
PNT5 2695	CAAT-box	CAAT	Nicotiana glutinosa	
PNT5 2695	CAAT-box	CAAAT	Pisum sativum	common cis-acting element in promoter and enhancer regions
PNT5 2695	CAAT-box	CAAAT	Pisum sativum	common cis-acting element in promoter and enhancer regions
PNT5 2695	CAAT-box	CAAT	Nicotiana glutinosa	
PNT5 2695	CAAT-box	CAAT	Nicotiana glutinosa	
PNT5 2695	CAAT-box	CAAT	Nicotiana glutinosa	
PNT5 2695	CAAT-box	CAAT	Nicotiana glutinosa	
PNT5 2695	CAAT-box	CAAAT	Pisum sativum	common cis-acting element in promoter and enhancer regions
PNT5 2695	CAAT-box	CAAT	Nicotiana glutinosa	
PNT5 2695	CAAT-box	CAAT	Nicotiana glutinosa	
PNT5 2695	CAAT-box	CAAT	Nicotiana glutinosa	
PNT5 2695	CAAT-box	CAAT	Nicotiana glutinosa	
PNT5 2695	CAAT-box	CAAAT	Pisum sativum	common cis-acting element in promoter and enhancer regions
PNT5 2695	Myb-binding site	CAACAG	Nicotiana tabacum	
PNT5 2695	AAGAA-motif	GAAAGAA	Avena sativa	
PNT5 2695	AAGAA-motif	GAAAGAA	Avena sativa	
PNT5 2695	AAGAA-motif	GAAAGAA	Avena sativa	
PNT5 2695	GATA-motif	AAGGATAAGG	Solanum tuberosum	part of a light responsive element
PNT5 2695	GATA-motif	GATAGGG	Pisum sativum	part of a light responsive element

PNT5 2695	ARE	AAACCA	Zea mays	cis-acting regulatory element essential for the anaerobic induction
PNT5 2695	ARE	AAACCA	Zea mays	cis-acting regulatory element essential for the anaerobic induction
PNT5 2695	G-Box	CACGTT	Pisum sativum	cis-acting regulatory element involved in light responsiveness
PNT5 2695	ABRE4	CACGTA	Zea mays	
PNT5 2695	GT1-motif	GGTTAA	Arabidopsis thaliana	light responsive element
PNT5 2695	GT1-motif	GGTTAA	Arabidopsis thaliana	light responsive element
PNT5 2695	GT1-motif	GGTTAA	Arabidopsis thaliana	light responsive element
PNT5 2695	AT~TATA-box	TATATAAA	Arabidopsis thaliana	
PNT5 2695	AT~TATA-box	TATATA	Arabidopsis thaliana	
PNT5 2695	AT~TATA-box	TATATA	Arabidopsis thaliana	
PNT5 2695	AT~TATA-box	TATATA	Arabidopsis thaliana	
PNT5 2695	Myb	TAACTG	Arabidopsis thaliana	
PNT5 2695	Myb	TAACTG	Arabidopsis thaliana	
PNT5 2695	ABRE3a	TACGTG	Zea mays	
PNT5 2695	Unnamed__6	taTAAATATct	Zea mays	
PNT5 2695	Unnamed__6	taTAAATATct	Zea mays	
PNT5 2695	G-box	TACGTG	Arabidopsis thaliana	cis-acting regulatory element involved in light responsiveness
PNT5 2695	TGA-element	AACGAC	Brassica oleracea	auxin-responsive element
PNT5 2695	MYB-like sequence	TAACCA	Arabidopsis thaliana	
PNT5 2695	MYB-like sequence	TAACCA	Arabidopsis thaliana	
PNT5 2695	MYB-like sequence	TAACCA	Arabidopsis thaliana	
PNT5 2695	MYB-like sequence	TAACCA	Arabidopsis thaliana	

PNT5 2695	TATA	TATAAAAT	Arabidopsis thaliana	
PNT5 2695	TATA	TATAAAAT	Arabidopsis thaliana	
PNT5 2695	TATA	TATAAAAT	Arabidopsis thaliana	
PNT5 2695		motif_sequence	organism	short_function
PNT5 2695		motif_sequence	organism	short_function
PNT5 2695		motif_sequence	organism	short_function
PNT5 2695		motif_sequence	organism	short_function
PNT5 2695		motif_sequence	organism	short_function
PNT5 2695		motif_sequence	organism	short_function
PNT5 2695		motif_sequence	organism	short_function
PNT5 2695		motif_sequence	organism	short_function
PNT5 2695		motif_sequence	organism	short_function
PNT5 2695	ERE	ATTTCATA	Nicotiana glutinos	
PNT5 2695	ERE	ATTTTAAA	Nicotiana glutinos	
PNT5 2695	ERE	ATTTCATA	Nicotiana glutinos	
PNT5 2695	Box 4	ATTAAT	Petroselinum crispum	part of a conserved DNA module involved in light responsiveness
PNT5 2695	Box 4	ATTAAT	Petroselinum crispum	part of a conserved DNA module involved in light responsiveness
PNT5 2695	Box 4	ATTAAT	Petroselinum crispum	part of a conserved DNA module involved in light responsiveness
PNT5 2695	Box 4	ATTAAT	Petroselinum crispum	part of a conserved DNA module involved in light responsiveness
PNT5 2695	Box 4	ATTAAT	Petroselinum crispum	part of a conserved DNA module involved in light responsiveness
PNT5 2695	Box 4	ATTAAT	Petroselinum crispum	part of a conserved DNA module involved in light responsiveness
PNT5 2695	MYB	TAACCA	Arabidopsis thaliana	

PNT52695	MYB	TAACCA	Arabidopsis thaliana	
PNT52695	MYB	CAACCA	Arabidopsis thaliana	
PNT52695	MYB	TAACCA	Arabidopsis thaliana	
PNT52695	MYB	CAACCA	Arabidopsis thaliana	
PNT52695	MYB	CAACCA	Arabidopsis thaliana	
PNT52695	MYB	CAACCA	Arabidopsis thaliana	
PNT52695	MYB	CAACAG	Arabidopsis thaliana	
PNT52695	MYB	TAACCA	Arabidopsis thaliana	
PNT58721	WRE3	CCACCT	Pisum sativum	
PNT58721	MYB-like sequence	TAACCA	Arabidopsis thaliana	
PNT58721	Y-box	TGTGGAGGAGCA	Lemna gibba	?
PNT58721	TGA-element	AACGAC	Brassica oleracea	auxin-responsive element
PNT58721	CARE	CAACTCAC	Oryza sativa	
PNT58721		motif_sequence	organism	short_function
PNT58721		motif_sequence	organism	short_function
PNT58721		motif_sequence	organism	short_function
PNT58721		motif_sequence	organism	short_function
PNT58721		motif_sequence	organism	short_function
PNT58721		motif_sequence	organism	short_function
PNT58721		motif_sequence	organism	short_function
PNT58721		motif_sequence	organism	short_function
PNT58721		motif_sequence	organism	short_function

PNT5 8721	MYB	TAACCA	Arabidopsis thaliana	
PNT5 8721	MYB	CAACAG	Arabidopsis thaliana	
PNT5 8721	AT~TATA-box	TATATA	Arabidopsis thaliana	
PNT5 8721	AT~TATA-box	TATATA	Arabidopsis thaliana	
PNT5 8721	AT~TATA-box	TATATA	Arabidopsis thaliana	
PNT5 8721	AT~TATA-box	TATATA	Arabidopsis thaliana	
PNT5 8721	CAT-box	GCCACT	Arabidopsis thaliana	cis-acting regulatory element related to meristem expression
PNT5 8721	GT1-motif	GGTTAAT	Avena sativa	light responsive element
PNT5 8721	GT1-motif	GGTTAA	Arabidopsis thaliana	light responsive element
PNT5 8721	P-box	CCTTTTG	Oryza sativa	gibberellin-responsive element
PNT5 8721	P-box	CCTTTTG	Oryza sativa	gibberellin-responsive element
PNT5 8721	chs-CMA2a	TCACTTGA	Petroselinum crispum	part of a light responsive element
PNT5 8721	chs-CMA2a	TCACTTGA	Petroselinum crispum	part of a light responsive element
PNT5 8721	TCA	TCATCTTCAT	Pisum sativum	
PNT5 8721	Unnamed__4	CTCC	Petroselinum hortense	
PNT5 8721	Unnamed__4	CTCC	Petroselinum hortense	
PNT5 8721	Unnamed__4	CTCC	Petroselinum hortense	
PNT5 8721	Unnamed__4	CTCC	Petroselinum hortense	
PNT5 8721	Unnamed__4	CTCC	Petroselinum hortense	
PNT5 8721	Unnamed__4	CTCC	Petroselinum hortense	
PNT5 8721	MYC	CATGTG	Arabidopsis thaliana	
PNT5 8721	MYC	CAATTG	Arabidopsis thaliana	

PNT5 8721	MYC	CATTTG	Arabidopsis thaliana	
PNT5 8721	MYC	CAATTG	Arabidopsis thaliana	
PNT5 8721	CAAT-box	CAAAT	Pisum sativum	common cis-acting element in promoter and enhancer regions
PNT5 8721	CAAT-box	CAAT	Nicotiana glutinosa	
PNT5 8721	CAAT-box	CAAT	Nicotiana glutinosa	
PNT5 8721	CAAT-box	CAAT	Nicotiana glutinosa	
PNT5 8721	CAAT-box	CAAT	Nicotiana glutinosa	
PNT5 8721	CAAT-box	CAAT	Nicotiana glutinosa	
PNT5 8721	CAAT-box	CAAT	Nicotiana glutinosa	
PNT5 8721	CAAT-box	CAAT	Nicotiana glutinosa	
PNT5 8721	CAAT-box	CAAT	Nicotiana glutinosa	
PNT5 8721	CAAT-box	CCAAT	Arabidopsis thaliana	common cis-acting element in promoter and enhancer regions
PNT5 8721	CAAT-box	CAAT	Nicotiana glutinosa	
PNT5 8721	CAAT-box	CAAT	Nicotiana glutinosa	
PNT5 8721	CAAT-box	CAAT	Nicotiana glutinosa	
PNT5 8721	CAAT-box	CAAAT	Pisum sativum	common cis-acting element in promoter and enhancer regions
PNT5 8721	CAAT-box	CCAAT	Arabidopsis thaliana	common cis-acting element in promoter and enhancer regions
PNT5 8721	CAAT-box	CAAAT	Pisum sativum	common cis-acting element in promoter and enhancer regions
PNT5 8721	CAAT-box	CAAT	Nicotiana glutinosa	
PNT5 8721	CAAT-box	CAAT	Nicotiana glutinosa	
PNT5 8721	CAAT-box	CAAT	Nicotiana glutinosa	
PNT5 8721	CAAT-box	CAAT	Nicotiana glutinosa	

PNT58721	CAAT-box	CAAAT	Pisum sativum	common cis-acting element in promoter and enhancer regions
PNT58721	CAAT-box	CAAAT	Pisum sativum	common cis-acting element in promoter and enhancer regions
PNT58721	CAAT-box	CAAT	Nicotiana glutinosa	
PNT58721	CAAT-box	CAAT	Nicotiana glutinosa	
PNT58721	CAAT-box	CAAT	Nicotiana glutinosa	
PNT58721	CAAT-box	CAAT	Nicotiana glutinosa	
PNT58721	CAAT-box	CAAT	Nicotiana glutinosa	
PNT58721	CAAT-box	CAAT	Nicotiana glutinosa	
PNT58721	CAAT-box	CAAAT	Pisum sativum	common cis-acting element in promoter and enhancer regions
PNT58721	CAAT-box	CAAAT	Pisum sativum	common cis-acting element in promoter and enhancer regions
PNT58721	CAAT-box	CAAT	Nicotiana glutinosa	
PNT58721	CAAT-box	CAAT	Nicotiana glutinosa	
PNT58721	CAAT-box	CAAT	Nicotiana glutinosa	
PNT58721	CAAT-box	CAAT	Nicotiana glutinosa	
PNT58721	Myb-binding site	CAACAG	Nicotiana tabacum	
PNT58721	WUN-motif	AAATTACT	Nicotiana glutinosa	
PNT58721	W box	TTGACC	Arabidopsis thaliana	
PNT58721	ARE	AAACCA	Zea mays	cis-acting regulatory element essential for the anaerobic induction
PNT58721	ARE	AAACCA	Zea mays	cis-acting regulatory element essential for the anaerobic induction
PNT58721	AE-box	AGAAACAA	Arabidopsis thaliana	part of a module for light response
PNT58721	ATCT-motif	AATCTAATCC	Pisum sativum	part of a conserved DNA module involved in light responsiveness
PNT58721	STRE	AGGGG	Arabidopsis thaliana	

PNT5 8721	TCA-element	CCATCTTTTT	Nicotiana tabacum	cis-acting element involved in salicylic acid responsiveness
PNT5 8721	Unnamed__1	CGTGG	Zea mays	
PNT5 8721	TATA-box	ATATAT	Brassica napus	core promoter element around -30 of transcription start
PNT5 8721	TATA-box	TATA	Arabidopsis thaliana	core promoter element around -30 of transcription start
PNT5 8721	TATA-box	TATAAA	Helianthus annuus	core promoter element around -30 of transcription start
PNT5 8721	TATA-box	TATAA	Arabidopsis thaliana	core promoter element around -30 of transcription start
PNT5 8721	TATA-box	TATA	Arabidopsis thaliana	core promoter element around -30 of transcription start
PNT5 8721	TATA-box	ATTATA	Brassica napus	core promoter element around -30 of transcription start
PNT5 8721	TATA-box	TATAA	Arabidopsis thaliana	core promoter element around -30 of transcription start
PNT5 8721	TATA-box	TATA	Arabidopsis thaliana	core promoter element around -30 of transcription start
PNT5 8721	TATA-box	TATA	Arabidopsis thaliana	core promoter element around -30 of transcription start
PNT5 8721	TATA-box	TATA	Arabidopsis thaliana	core promoter element around -30 of transcription start
PNT5 8721	TATA-box	ATTATA	Brassica napus	core promoter element around -30 of transcription start
PNT5 8721	TATA-box	TATATAA	Arabidopsis thaliana	core promoter element around -30 of transcription start
PNT5 8721	TATA-box	TATATA	Arabidopsis thaliana	core promoter element around -30 of transcription start
PNT5 8721	TATA-box	ATATAT	Brassica napus	core promoter element around -30 of transcription start
PNT5 8721	TATA-box	TATATTTATATT T	Avena sativa	core promoter element around -30 of transcription start
PNT5 8721	TATA-box	ATATAT	Brassica napus	core promoter element around -30 of transcription start
PNT5 8721	TATA-box	TATATA	Arabidopsis thaliana	core promoter element around -30 of transcription start
PNT5 8721	TATA-box	ATATAT	Brassica napus	core promoter element around -30 of transcription start
PNT5 8721	TATA-box	TATATA	Arabidopsis thaliana	core promoter element around -30 of transcription start
PNT5 8721	TATA-box	ATATAT	Brassica napus	core promoter element around -30 of transcription start

PNT58721	TATA-box	TATA	Arabidopsis thaliana	core promoter element around -30 of transcription start
PNT58721	TATA-box	ATTATA	Brassica napus	core promoter element around -30 of transcription start
PNT58721	TATA-box	TATAA	Arabidopsis thaliana	core promoter element around -30 of transcription start
PNT58721	TATA-box	TATA	Arabidopsis thaliana	core promoter element around -30 of transcription start
PNT58721	TATA-box	TATAA	Arabidopsis thaliana	core promoter element around -30 of transcription start
PNT58721	TATA-box	TATA	Arabidopsis thaliana	core promoter element around -30 of transcription start
PNT58721	TATA-box	ATATAA	Brassica oleracea	core promoter element around -30 of transcription start
PNT58721	TATA-box	TATA	Arabidopsis thaliana	core promoter element around -30 of transcription start
PNT58721	TATA-box	TATA	Arabidopsis thaliana	core promoter element around -30 of transcription start
PNT58721	TATA-box	TATAAGAA	Zea mays	core promoter element around -30 of transcription start
PNT58721	TATA-box	TATAA	Arabidopsis thaliana	core promoter element around -30 of transcription start
PNT58721	TATA-box	TATA	Arabidopsis thaliana	core promoter element around -30 of transcription start
PNT58721	TATA-box	TATAAAA	Pisum sativum	core promoter element around -30 of transcription start
PNT58721	TATA-box	TATAAA	Helianthus annuus	core promoter element around -30 of transcription start
PNT58721	TATA-box	TATAA	Arabidopsis thaliana	core promoter element around -30 of transcription start
PNT58721	TATA-box	TATA	Arabidopsis thaliana	core promoter element around -30 of transcription start
PNT58721	TATA-box	TACATAAA	Oryza sativa	core promoter element around -30 of transcription start

Table S2: *PtCAF1* gene duplication pairs (synteny relationship) in *PtCAF1* gene family.

gene ID	gene ID
Potri.004G200400	Potri.009G161500
Potri.006G205600	Potri.009G161500
Potri.009G161500	Potri.016G073000
Potri.004G200400	Potri.006G205600
Potri.004G200400	Potri.016G073000
Potri.003G181100	Potri.006G262500
Potri.003G181100	Potri.018G020900

Potri.001G046700	Potri.003G181100
Potri.006G187200	Potri.006G262500
Potri.006G262500	Potri.018G020900
Potri.001G046700	Potri.006G262500
Potri.006G187200	Potri.018G020900
Potri.006G205600	Potri.016G073000
Potri.001G046700	Potri.018G020900
AT1G80780	Potri.001G046700
AT1G80780	Potri.003G181100
AT5G10960	Potri.006G262500
AT5G10960	Potri.018G020900
Potri.006G187200	LOC_Os02g55300
Potri.006G262500	LOC_Os02g55300
Potri.018G020900	LOC_Os02g55300

Table S3: *CAF1* gene family in various species.

geneID	geneName	species
At1G06450.1	ATCAF1G	Arabidopsis thaliana
At1G15920.2	ATCAF1H	Arabidopsis thaliana
At1G27820.1	ATCAF1C	Arabidopsis thaliana
At1G27890.1	ATCAF1D	Arabidopsis thaliana
At1G55870.1	AHG2	Arabidopsis thaliana
At1G61470.1	ATCAF1E	Arabidopsis thaliana
At1G80780.3	ATCAF1J	Arabidopsis thaliana
At2G32070.1	ATCAF1K	Arabidopsis thaliana
At3G25430.1	RRD1	Arabidopsis thaliana
At3G44240.1	ATCAF1F	Arabidopsis thaliana
At3G44260.1	ATCAF1A	Arabidopsis thaliana
At5G10960.1	ATCAF1I	Arabidopsis thaliana
At5G22250.1	ATCAF1B	Arabidopsis thaliana
LOC_Os02g55300.1	OsCAF1H	Oryza sativa
LOC_Os03g53260.1	OsCAF1Q	Oryza sativa
LOC_Os04g58810.1	OsCAF1B (L)	Oryza sativa
LOC_Os06g04670.1	OsCAF1D	Oryza sativa
LOC_Os06g30770.1	OsCAF1L	Oryza sativa
LOC_Os06g30790.1	OsCAF1K	Oryza sativa
LOC_Os06g34520.1	OsCAF1J	Oryza sativa
LOC_Os07g07430.1	OsCAF1C	Oryza sativa
LOC_Os08g34170.1	OsCAF1A	Oryza sativa
LOC_Os09g24990.2	OsCAF1G	Oryza sativa
LOC_Os10g03530.1	OsCAF1F	Oryza sativa
LOC_Os10g03560.1	OsCAF1I	Oryza sativa
LOC_Os10g03880.1	OsCAF1E	Oryza sativa
LOC_Os10g28450.1	OsCAF1M	Oryza sativa

LOC_Os10g28520.1	OsCAF1O	Oryza sativa
LOC_Os10g28540.1	OsCAF1P	Oryza sativa
LOC_Os10g28580.1	OsCAF1R	Oryza sativa
LOC_Os10g28590.1	OsCAF1N	Oryza sativa
Potri.001G038500.1	PtCAF1A	Populus trichocarpa
Potri.001G039000.1	PtCAF1B	Populus trichocarpa
Potri.001G040400.1	PtCAF1C	Populus trichocarpa
Potri.001G046700.1	PtCAF1D	Populus trichocarpa
Potri.001G368400.1	PtCAF1E	Populus trichocarpa
Potri.003G181100.1	PtCAF1F	Populus trichocarpa
Potri.003G186300.1	PtCAF1G	Populus trichocarpa
Potri.004G048800.1	PtCAF1H	Populus trichocarpa
Potri.004G200400.1	PtCAF1I	Populus trichocarpa
Potri.006G187200.1	PtCAF1J	Populus trichocarpa
Potri.006G205600.1	PtCAF1K	Populus trichocarpa
Potri.006G262500.1	PtCAF1L	Populus trichocarpa
Potri.009G161500.1	PtCAF1M	Populus trichocarpa
Potri.014G018500.1	PtCAF1N	Populus trichocarpa
Potri.014G177400.1	PtCAF1O	Populus trichocarpa
Potri.016G073000.1	PtCAF1P	Populus trichocarpa
Potri.018G020900.1	PtCAF1Q	Populus trichocarpa
Potri.018G036100.1	PtCAF1R	Populus trichocarpa
Potri.018G038700.1	PtCAF1S	Populus trichocarpa
Eucgr.B03817.1	EucgrCAF1A	Eucalyptus grandis
Eucgr.C01665.1	EucgrCAF1C	Eucalyptus grandis
Eucgr.C01670.1	EucgrCAF1B	Eucalyptus grandis
Eucgr.C01976.1	EucgrCAF1E	Eucalyptus grandis
Eucgr.C01981.1	EucgrCAF1D	Eucalyptus grandis
Eucgr.C02596.1	EucgrCAF1F	Eucalyptus grandis
Eucgr.F02044.1	EucgrCAF1G	Eucalyptus grandis
Eucgr.F03953.1	EucgrCAF1H	Eucalyptus grandis
Eucgr.G00324.1	EucgrCAF1I	Eucalyptus grandis
Eucgr.H00715.1	EucgrCAF1J	Eucalyptus grandis
Eucgr.J00535.1	EucgrCAF1K	Eucalyptus grandis
Eucgr.J03104.1	EucgrCAF1L	Eucalyptus grandis
XP_010907244.1	ElaguCAF1A	Elaeis guineensis
XP_010911571.1	ElaguCAF1B	Elaeis guineensis
XP_010912355.2	ElaguCAF1C	Elaeis guineensis
XP_010920209.1	ElaguCAF1D	Elaeis guineensis
XP_010927524.1	ElaguCAF1E	Elaeis guineensis
XP_010929136.1	ElaguCAF1F	Elaeis guineensis
XP_010932239.1	ElaguCAF1G	Elaeis guineensis
XP_010932240.1	ElaguCAF1H	Elaeis guineensis

XP_010932706.1	ElaguCAF1I	Elaeis guineensis
XP_010942117.1	ElaguCAF1J	Elaeis guineensis
XP_010942841.1	ElaguCAF1K	Elaeis guineensis
XP_010943605.1	ElaguCAF1L	Elaeis guineensis
XP_019701436.1	ElaguCAF1M	Elaeis guineensis
XP_019701600.1	ElaguCAF1N	Elaeis guineensis
XP_029116591.1	ElaguCAF1O	Elaeis guineensis

Table S4: List of primers for qRT-PCR analysis in this study.

gene ID	gene name	forward primer	reverse primer
Potri.001G038500.1	PtCAF1A	GGATTGGAGAGGGTGGCTAT	CAGCCTTCACACAGTCCAAA
Potri.001G039000.1	PtCAF1B	ATGAAGAGCAGCGAACCAGT	GACAGCGAACTCTTGTCACG
Potri.001G040400.1	PtCAF1C	GTTGCCAAGTGATATGCGGT	TGGTGCCTTCTTCCCGTTAT
Potri.001G046700.1	PtCAF1D	TGGTGAGCTCTTGATGTCGT	TGCAAGCTTGTTCAACCCTC
Potri.001G368400.1	PtCAF1E	AGGTTGAGAGAATGGCGTGA	CCTAAGCTGACGGGAAGTGA
Potri.003G181100.1	PtCAF1F	TGGTGAGCTCTTGATGTCGT	TGCAAGCTTGTTCAACCCTC
Potri.003G186300.1	PtCAF1G	TGGAGAGGGTGGCTAAGTTG	TCCCTCACAGCCATTCAAGT
Potri.004G048800.1	PtCAF1H	ACTAGTTTTGGGGCATGGGA	GCCAAGAGAAGTAGCCCAGA
Potri.004G200400.1	PtCAF1I	TTTCCGACCACCAGTAGACC	CAATCGAGTCAGGAGCATGC
Potri.006G187200.1	PtCAF1J	GTCTTCACGGTGGGTTGAAC	ACCAGCATATCTCTCAGGGG
Potri.006G205600.1	PtCAF1K	TAGGACTCACGATCGCTGAC	ACTGAGTCAATCCCGAGCTC
Potri.006G262500.1	PtCAF1L	GTTGAACAAGCTTGCGGAGT	TCGACACCAAGACCGTACAA
Potri.009G161500.1	PtCAF1M	GGACCCCACCAATAACAGGA	ACTCCCAGATGAACCGGTTT
Potri.014G018500.1	PtCAF1N	GATTTTGTGGCCGTGTCCTT	GACAGTGATGGGGCAAACAG
Potri.014G177400.1	PtCAF1O	GCTGCGCCATTGTGATTTTC	TTCGGAAGCACAGTCAGAGT
Potri.016G073000.1	PtCAF1P	AAACTCCGATAAACAGCCGC	GCCGCTATGCCGATTATACG

Potri.018G020900.1	PtCAF1Q	GCTTGCAGAGTTGTTGGAGG	TCGACACCAAGACCGTACAA
Potri.018G036100.1	PtCAF1R	ACTCTTTGTGTGGGCTGCTT	CAGCTTGCATGAAACCAGAA
Potri.018G038700.1	PtCAF1S	TTGTCACGTGTTGTTGCCAA	CTCACCACCACGTAATCCCT
Potri.019G006700.1	PtActin	GACCTTCAATGTGCCTGCAA	ACCATCACCAGAATCCAGCA
Potri.006G130900.1	EF1α	TCCGTCTTCCACTTCAGGATGTCT	GTCACGACCATACCAGGCTTCAG
